# Characteristics of Neuropsychiatric Mobile Health Trials: Cross-Sectional Analysis of Studies Registered on ClinicalTrials.gov

**DOI:** 10.2196/16180

**Published:** 2020-08-04

**Authors:** Mia Tova Minen, Julia Frederica Reichel, Pallavi Pemmireddy, Elizabeth Loder, John Torous

**Affiliations:** 1 NYU Langone Health New York, NY United States; 2 Barnard College of Columbia University New York, NY United States; 3 Brigham and Women’s Hospital Boston, MA United States; 4 Beth Israel Deaconess Medical Center Brookline, MA United States

**Keywords:** smartphones, mobile phones, apps, mental health, regulation, stroke, migraine, major depressive disorder, Alzheimer disease, anxiety disorders, alcohol use disorders, opioid use disorders, epilepsy, schizophrenia

## Abstract

**Background:**

The development of mobile health (mHealth) technologies is progressing at a faster pace than that of the science to evaluate their validity and efficacy. Under the International Committee of Journal Medical Editors (ICMJE) guidelines, clinical trials that prospectively assign people to interventions should be registered with a database before the initiation of the study.

**Objective:**

The aim of this study was to better understand the smartphone mHealth trials for high-burden neuropsychiatric conditions registered on ClinicalTrials.gov through November 2018, including the number, types, and characteristics of the studies being conducted; the frequency and timing of any outcome changes; and the reporting of results.

**Methods:**

We conducted a systematic search of ClinicalTrials.gov for the top 10 most disabling neuropsychiatric conditions and prespecified terms related to mHealth. According to the 2016 World Health Organization Global Burden of Disease Study, the top 10 most disabling neuropsychiatric conditions are (1) stroke, (2) migraine, (3) major depressive disorder, (4) Alzheimer disease and other dementias, (5) anxiety disorders, (6) alcohol use disorders, (7) opioid use disorders, (8) epilepsy, (9) schizophrenia, and (10) other mental and substance use disorders. There were no date, location, or status restrictions.

**Results:**

Our search identified 135 studies. A total of 28.9% (39/135) of studies evaluated interventions for major depressive disorder, 14.1% (19/135) of studies evaluated interventions for alcohol use disorders, 12.6% (17/135) of studies evaluated interventions for stroke, 11.1% (15/135) of studies evaluated interventions for schizophrenia, 8.1% (11/135) of studies evaluated interventions for anxiety disorders, 8.1% (11/135) of studies evaluated interventions for other mental and substance use disorders, 7.4% (10/135) of studies evaluated interventions for opioid use disorders, 3.7% (5/135) of studies evaluated interventions for Alzheimer disease or other dementias, 3.0% (4/135) of studies evaluated interventions for epilepsy, and 3.0% (4/135) of studies evaluated interventions for migraine. The studies were first registered in 2008; more than half of the studies were registered from 2016 to 2018. A total of 18.5% (25/135) of trials had results reported in some publicly accessible location. Across all the studies, the mean estimated enrollment (reported by the study) was 1078, although the median was only 100. In addition, across all the studies, the actual reported enrollment was lower, with a mean of 249 and a median of 80. Only about a quarter of the studies (35/135, 25.9%) were funded by the National Institutes of Health.

**Conclusions:**

Despite the increasing use of health-based technologies, this analysis of ClinicalTrials.gov suggests that only a few apps for high-burden neuropsychiatric conditions are being clinically evaluated in trials.

## Introduction

The field of mobile health (mHealth), broadly defined as health care interventions that are delivered by mobile devices such as smartphones and tablets, is growing rapidly. Currently, there are over 325,000 mHealth apps [1], and the field of mHealth continues to attract new market entrants (28% of digital health practitioners have less than 2 years of industry experience) [1]. In addition, the global mHealth app market size is expected to hit 236 billion by 2026 [2]. mHealth apps claim to have varied purposes, from improving treatment adherence to increasing physical activity, to supplementing in-person counseling, and much more. The development of mHealth technologies is currently progressing at a much faster pace than that of the science to evaluate their validity and efficacy [3]. Thus, it is possible that ineffective or even harmful mHealth technologies might enter clinical practice without adequate evaluation [4]. For example, many apps make scientific claims on the app stores, but less than 2% of the apps can offer clinical evidence to back such claims [5]. The topic of digital health regulation by the Food and Drug Administration in the United States has been a news story since the fall of 2019 when it was discussed by a presidential candidate.

Currently, one path to increase scientific validity and transparency in the digital health space is through the mechanisms in place around publication. Since 2005, a condition of consideration for publication in the International Committee of Journal Medical Editors (ICJME) has been prospective registration of clinical trials. The ICJME defines a clinical trial as “any research project that prospectively assigns people or a group of people to an intervention, with or without concurrent comparison or control groups, to study the relationship between a health-related intervention and a health outcome.” Trial registration is supposed to occur “in a public trials registry at or before the time of first patient enrollment.” Registration requirements are intended to prevent well-documented problems that arise when the results of the trials are either unreported or are selectively reported [6]. A majority of the clinical trials are registered at ClinicalTrials.gov, a division of the US National Library of Medicine [7]. It is also possible to post trial results on ClinicalTrials.gov, although such posting is only required for certain studies. When reporting requirements apply, the results must generally be posted no later than 1 year after final data collection for the primary outcome [8].

Previous examinations of ClinicalTrials.gov have provided details on the types of studies being conducted in various fields and the various aspects of such studies, for example, location, number of study participants, interventions, and funders [9,10]. As is the case with clinical trials, in general, it is likely that many trials of mHealth interventions are not compliant with the requirements for trial registration. Selective reporting of results is of special concern in mHealth studies as they often collect vast amounts of data from surveys and sensors—often millions of data points from smartphone sensors and wearables. This creates many opportunities for both intentional and inadvertent systematic errors or biases in ongoing data collection, analysis, and interpretation. Prospective trial registration is an important protection against such mistakes, as well as a safeguard against intentional abuse.

However, the characteristics, number, and quality of registered trials of mHealth interventions for most therapeutic areas, including neuropsychiatric disorders, are unknown. According to the 2016 World Health Organization (WHO) Global Burden of Disease Study [11], the top 10 most disabling noninfectious neuropsychiatric conditions are, in the following order of ranking, (1) stroke, (2) migraine, (3) major depressive disorder, (4) Alzheimer disease and other dementias, (5) anxiety disorders, (6) opioid use disorders, (7) alcohol use disorders, (8) epilepsy, (9) schizophrenia, and (10) other mental and substance use disorders. For these conditions, we sought to evaluate the (1) number of mHealth trials of smartphone interventions registered on ClinicalTrials.gov; (2) study characteristics, for example, location of the study, type of interventions studied, number of study participants, length of study, and funder (National Institutes of Health [NIH] or other), among other characteristics; (3) frequency and timing of any outcome changes in the trial registry; and (4) the proportion of such studies that had reported results on ClinicalTrials.gov or in journal publications. Neuropsychiatric disorders comprise a broad range of medical conditions involving neurologic or psychiatric disturbances, including mental and behavioral disorders. Globally, they are the third leading cause of disability-adjusted life years (DALYs) [12,13]. Neuropsychiatric conditions are likely to be particularly amenable to mHealth interventions as their treatment may involve behavioral therapies and efforts to improve medication adherence, and there is a great need for scalable and accessible interventions [14-16].

## Methods

According to the 2016 WHO Global Burden of Disease Study, [11] the following comprised the top 10 most disabling neuropsychiatric conditions worldwide: (1) stroke, (2) migraine, (3) major depressive disorder, (4) Alzheimer disease and other dementias, (5) anxiety disorders, (6) alcohol use disorders, (7) opioid use disorders, (8) epilepsy, (9) schizophrenia, and (10) other mental and substance use disorders (Multimedia Appendix 1 [17-149]). These conditions were searched for in ClinicalTrials.gov, along with smartphone-based keyword terms, and data were abstracted accordingly.

Data were summarized descriptively. Of note, we examined the reporting of results using two different methods. In the first method, for studies registered 3 or more years prior, we examined how many of these studies reported results either via one or both of the following: results reported on ClinicalTrials.gov or results automatically indexed to ClinicalTrials.gov. In the second method, we examined the studies marked completed on ClinicalTrials.gov by October 2017 (1 year before the date of data abstraction) and then examined which of those studies reported results either on ClinicalTrials.gov and/or had results automatically indexed to ClinicalTrials.gov.

We also analyzed whether there were any associations among (1) the number of study participants and study completion status, (2) the number of study participants and study results reporting status (3), the length of study intervention and completion status, and (4) the length of study intervention and results reporting status. We utilized *t* tests and regression models to analyze the results. A statistical analysis was conducted in the R programming environment (version R 3.6.1; The R Foundation).

Per the self-documentation form from the New York University (NYU) School of Medicine (SOM), the research did not involve human subjects. Thus, consistent with the NYU SOM institutional review board (IRB) policy and federal regulations governing human subject research, an IRB review was not required.

## Results

A total of 135 studies on ClinicalTrials.gov met the search criteria. As shown in Multimedia Appendix 2 [17-64,66-82,84-114,116-133,135-141,143-153], the number of studies registered for each neuropsychiatric condition that used a smartphone for an intervention was as follows: major depressive disorder (n=39), alcohol use disorders (n=19), stroke (n=17), schizophrenia (n=15), anxiety disorders (n=11), other mental and substance use disorders (n=11), opioid use disorders (n=10), Alzheimer disease and other dementias (n=5), epilepsy (n=4), and migraine (n=4). Multimedia Appendix 1 shows the key findings for the various apps, including the myriad of purposes they served. They ranged from promoting rehabilitation and diet to medication adherence, symptom tracking, cognitive behavioral therapy, and more.

A breakdown of the studies is provided in Table 1. The altered outcomes have been described in Table 2.

**Table 1:** Breakdown of study status, results reporting, location of studies and National Institutes of Health funding.

Characteristics^a,b^		All conditions	Stroke	Migraine	Major depressive disorders	Alzheimer disease and other dementias	Anxiety disorders	Alcohol use disorders	Opioid use disorders	Epilepsy	Schizophrenia	Other mental and substance use disorders
Number of apps, n		135	17	4	39	5	11	19	10	4	15	11
**Status of study, n**												
	Not yet recruiting	19	0	2	5	3	0	1	4	0	2	2
	Recruiting	40	8	2	9	1	2	7	3	2	3	3
	Enrolling by invitation	7	1	0	0	1	2	1	0	1	0	1
	Active, not recruiting	7	1	0	4	0	0	1	0	0	1	0
	Completed	59	7	0	21	0	8	7	3	1	9	4
	Unknown	3	0	0	0	0	0	2	0	0	0	1
**Number of participants estimated**												
	Mean (SD)	1078.64 (9088.35)	222.06 (470.64)	131 (52.57)	199.74 (347.83)	196 (175.23)	629 (1640.23)	439.59 (866.02)	144.8 (205.22)	25066.25 (49955.84)	102 (92.31)	234.10 (241.73)
	Lowest; highest, n	8; 100,000	8; 2000	90; 200	15; 2000	52,448	8; 5000	30; 3600	9; 600	65; 100,000	36; 260	40; 800
	Median (IQR)	100 (150)	80 (160)	117 (68)	103 (123)	142 (147)	70 (110)	105 (210)	64.5 (105.5)	100 (24983.75)	42.5 (113)	100 (303.5)
**Number of participants actual**												
	Mean (SD)	242.19 (586.02)	301.55 (657.80)	N/A^c^	208.38 (426.21)	20 (N/A)	51.4 (41.52)	683.71 (1356.83)	67 (79.20)	95 (N/A)	92.3 (75.69)	322.33 (123.44)
	Lowest; highest, n	10; 3702	20; 2274	N/A	11; 2010	20; 20	10; 105	15; 3702	11; 123	95; 95	27; 255	180; 400
	Median (IQR)	80 (137.25)	100 (145)	N/A	84.5 (105)	20 (0)	40 (66)	58 (395.5)	67 (56)	95 (0)	59.5 (91.25)	387 (110)
**Number of days of intervention**												
	Mean (SD)	149.26 (199.76)	155.88 (137.44)	173.75 (138.29)	104.44 (92.29)	202.2 (241.06)	198.91 (540.84)	125.90 (114.42)	158.6 (227.36)	112.25 (47.282)	189.93 (166.73)	205.18 (145.18)
	Lowest; highest, n	0.0083; 1825	1; 456	56; 365	1; 395	28; 548	0.00; 1825	7; 365	7; 730	84; 183	30; 548	30; 548
	Median (IQR)	84 (127)	91 (187)	137 (146.25)	84 (66)	42 (337)	14 (76)	84 (78.5)	56 (121.5)	91 (24.75)	168 (95.5)	183 (80.5)
**Regional distribution, n**												
	Africa	1	1	0	0	0	0	0	0	0	0	0
	Asia and Pacific	8	3	0	0	1	0	3	0	0	1	0
	Central+South America	5	0	0	4	0	1	0	0	0	0	0
	Europe	28	6	0	7	1	2	5	0	1	4	2
	Middle East	2	0	0	0	0	1	0	0	0	1	0
	North America	96	7	4	30	3	7	11	10	4	11	9
Study marked as National Institutes of Health funded, n		35	3	0	14	1	3	3	5	0	3	3

^a^Publication automatically indexed to the study by ClinicalTrials.gov identifier (NCT number) containing information pertaining to study without results.

^b^Publication automatically indexed to the study by ClinicalTrials.gov identifier (NCT number) containing information pertaining to study with results.

^c^N/A: not applicable.

**Table 2:** Altered outcomes.

Disorder and study title^a^		Altered outcomes
**Stroke**		
	Effects of Patient-centered Stroke Educating System: A Randomized Controlled Trial [152]	Original Primary Outcome Measures (submitted: October 28, 2015): Stroke Health Education Knowledge (time frame: 2 weeks) Changed August 16, 2018: Stroke Health Education Knowledge Questionnaire (time frame: 4 weeks)
	Empowerment and Mobile Technology in the Control of Cardiovascular Risk Factors in Patients with Ischemic Stroke (CARDIOSTROKE) [24]	Original Primary Outcome Measures (submitted: October 17, 2018) was Atrial Fibrillation (3 weeks) as detected in a 3-week ECG^b^ monitoring period and Systolic/diastolic blood pressure (time frame: 12 months) measured as difference in systolic/diastolic blood pressure. Current Primary Outcome Measures (submitted: October 18, 2018) are Number of Participants with New Atrial Fibrillation and Change in Blood Pressure. Current Secondary Outcome Measures (submitted: October 18, 2018)-Number of participants was added before new cardiovascular events within 12 months and new cardiovascular events within 36 months.
**Migraine**		
	RELAXaHEAD for Headache Patients [33]	Changed December 29, 2017: Proportion of patients who enrolled in the study/were recruited for the study was eliminated as a primary outcome measure. Satisfaction using Likert scale questions on RELAXaHEAD usability, content, functionality was added as a primary outcome measure.
**Major depressive disorder**		
	mHealth for Antenatal Mental Health [112]	Submitted September 9, 2016: Adherence to sampling protocol (time frame: assessed after 6 months) was added as a primary outcome measure.
	Text-Message-Based Depression for High-Risk Youth in the ED [41]	Submitted: January 5, 2015: Δ in Depressive Symptoms was the primary outcome measure. Submitted November 7, 2017- Δ in Peer Violence Involvement was added as a primary outcome measures. Submitted January 5, 2015-original secondary outcome measures was Δ in Peer Violence Involvement. Submitted January 5, 2015: Acceptability/Feasibility: Follow Up Rate, Acceptability/Feasibility: Engagement of Intervention Group and Acceptability/Feasibility: Participant Questionnaire were the secondary outcome measures.
	Behavioural Activation-Based Treatment Administered Through Smartphone [114]	Original Primary Outcome Measures (submitted: October 31, 2011) was the Montgomery Asberg Depression Rating Scale-Self Rated (MADRS). Current Primary Outcome Measures (submitted: March 22, 2013) is the PHQ-9^c^ and Beck Depression Inventory (BDI). Original Secondary Outcome Measures (submitted: March 22, 2013) was the QOLI^d^, AAQ^e^, BAI^f^ and TIC-P^g^. Current Secondary Outcome Measures (submitted March 22, 2013) eliminated the AAQ and BDI^h^ as secondary outcome measures.
	Mobile Technology to Engage and Link Patients and Providers in Antidepressant Treatment (MedLink) [116]	Original Primary Outcome Measures (submitted: October 20, 2015) was Adherence to Antidepressant Medication measured as the number of days medication was taken when a dose was expected. Current Primary Outcome Measures (submitted: February 14, 2018) is Adherence to Antidepressant Medication measured through % of days adherent on Wisepill pillbox as well as self-reported adherence. Original Secondary Outcome Measures (submitted: October 20, 2015) was changes in depression measured through self-report PHQ-9 and usability measured through Likert scale ratings. Current secondary outcome measures (submitted: February 14, 2018) is the PHQ-9 and Quick Inventory of Depressive Symptomology Clinician Rating (QIDS-C).
	Mobile Technology to Engage and Link Patients and Providers in Antidepressant Treatment [117]	Original Primary Outcome Measures (submitted: July 24, 2013) was Adherence to Medication as measured by when the provided pill bottle is opened to remove a dose of medication and Δ in Depression Over Time through the PHQ-9. Current Primary Outcome Measures (submitted: January 12, 2015) is measured as Adherence to Antidepressant Medication measured as the frequency of medication usage from baseline to end of treatment. Original Secondary Outcome Measures (submitted: July 24, 2013) was Presence of Side Effects and Δ s Over Time measured by the Patient Rated Inventory of Side Effects (PRISE) and Frequency, Intensity and Burden of Side Effects Rating (FIBSER). Current Secondary Outcome Measures (submitted: January, 12 2015) is changes in depression measured as the severity of depressive symptoms from baseline to end of treatment.
	Lifestyle Intervention for Young Adults with Serious Mental Illness [57]	Submitted: June 27, 2016 Δ in serum lipids was added as a current secondary outcome measures.
	Treating Depression on a Day-to-day Basis: Development of a Tool for Physicians Based on a Smartphone Application [122]	Original Primary Outcome Measures (submitted: September 18, 2018) was comparison between the two groups of the number of participants with a decrease in HDRSS^i^ scores of at least 50% at 8 weeks. Current Primary Outcome Measures (submitted: September 20, 2018) is a greater clinical response in the active group (smartphone application) comparatively to the comparator group (clinical response was defined as a decline in HDRS-17^j^ score greater than 50%).
	Using Mental Health Telemetry to Predict Relapse and Re-hospitalization in Mood Disorders (PATH-MOD) [54]	Submitted: November 21, 2014 Quality-of-Life in Bipolar Disorder was added as a Primary Outcome Measures.
	IntelliCare: Artificial Intelligence in a Mobile Intervention for Depression and Anxiety (AIM) [125]	Original primary outcome measures (submitted: June 25, 2014) were changes in depression severity, adherence to mobile application intervention and changes in anxiety. Current Primary Outcome Measures (submitted: February 14, 2018) are the PHQ-9 and GAD-7^k^. Original Secondary Outcome Measures was participant satisfaction. Current Secondary Outcome Measures is the Mean Number of Treatment App Use Sessions by Study Week.
	Enhancing Delivery of Problem Solving Therapy Using SmartPhone Technology [128]	Original Primary Outcome Measures (submitted: June 28, 2013) was Depression, Anxiety and Stress, an instrument that measures clinical indices of depression and anxiety as well as acute stress. Current Primary Outcome Measures (submitted: July 27, 2016) is the Depression Anxiety and Stress Scale (DASS).
	Technology Assisted Programs that Promote Mental Health for Teenagers (ProjectTECH) [48]	Original Primary Outcome Measures (submitted: July 30, 2013) were Depression (Time Frame: Throughout participation, lasting up to 26 weeks) as measured by the CES-D^l^ and MINIKid and Usability of the Program as measured by the Usefulness, Satisfaction and Ease of Use Questionnaire survey
	Study of Technology-assisted Treatment of Adolescent Depression (iTAD) [49]	Original Secondary Outcome Measures (submitted: April 19, 2012) was preliminary indicators of program efficacy (Time Frame: 12 weeks) measured by the 1. Depression Knowledge Test 2. Skill Self Efficacy Questionnaire 3. The Therapeutic Alliance Scale for Adolescents 4. The Cognitive Therapy Scale 5. Acceptance Questionnaire
	Mobile Phone Sensing and Outreach as Adjuncts to Internet Based Behavior Intervention for Depression [129]	Original Primary Outcome Measures (submitted: April 19, 2010) was Depression, as assessed by Quick Inventory of Depressive Symptoms, PHQ-9 and the Mini International Neuropsychiatric Interview Major Depressive Disorders Module (Time Frame: Measured as baseline, 4 and 8 weeks) Original Secondary Outcome Measures (submitted: April 19, 2010) was Utilization-Adherence markers for the mobile phone (eg-number of responses to prompts for information) the website (Time frame: Measured from baseline to 8 weeks), Positive Affect, Anxiety (GAD-7) and Health-Related Quality of Life.
	Effectiveness of a Technology Assisted Behavioral Intervention in Assisting People with Major Depressive Disorder [50]	Original Primary Outcome Measures (submitted: July 18, 2008) was Depression, as assessed by HRSD^m^, PHQ-9 and SCID and Attrition (in arms with i-CBT^n^) Original Secondary Outcome Measures was i-CBT utilization (eg, number of logins, average visit length, total time spent on the site, number of exercises completed), Health-Related Quality of Life (SF-36V^o^) and Patient Satisfaction (Satisfaction Index-Mental Health)
	Online Peer Networked Collaborative Learning for Managing Depressive Symptoms (MoodTech) [51]	Original Primary Outcome Measures (submitted: July 20, 2016) was Depression (Time Frame: Baseline to end of treatment) measured as the Δ in self-reported depressive symptom severity from baseline to end of treatment and adherence to the program measured as the number of times the program is accessed from start to last use from baseline to end of treatment
**Anxiety**		
	Connection to Care: Pilot Study of a Mobile Health Tool for Patients with Depression and Anxiety [131]	Original Primary Outcome Measures (submitted: July 13, 2015) were patient acceptability as determined by qualitative interview, care manager acceptability as determined by qualitative interview, patient report of usefulness as determined by qualitative interview and care manager report of usefulness as determined by qualitative interview. Current primary outcome measures (submitted: October 25, 2017) are app acceptability as measured by number of patient app users who rate app easy to use and time spent reasonable, app acceptability as measured by number of care manager dashboard users who rate dashboard easy to use and time spent reasonable, app usefulness as measured by number of patient app users who rate app easy to use and time spent reasonable and app usefulness as measured by number of care manager dashboard users who rate dashboard as useful.
	Improving Medical Care With Electronic Interventions Based on Automated Text and Phone Messages [132]	Current Secondary Outcome Measures (submitted: February 10, 2017)-Breastfeeding duration was added as a current secondary outcome measure.
	Effect of Premedication Type on Preoperative Anxiety in Children [77]	Current Primary Outcome Measures (submitted: August 13, 2018)-Mask acceptance (At anesthesia induction) was eliminated as a primary outcome measure and added as a secondary outcome measure.
	Using Smartphones to Enhance the Treatment of Childhood Anxiety [80]	Original Primary Outcome Measures (submitted: October 3, 2014) was the Clinical Global Impression Improvement (CGI-I) defined as treatment response at post treatment. Current Primary Outcome Measures (submitted: January 5, 2016) is the PARS^p^ Treatment response. Original Secondary Outcome Measures was the PARS and Screen for Childhood Anxiety Related Emotional Disorders (SCARED). Current Secondary Outcome Measures is the Absence of diagnosis on K-SADS^q^
	Youth Mayo Clinic Anxiety Coach Pilot Study [81]	Current Primary Outcome Measures (submitted: July 30, 2014)-qualitative interview assessing subject safety and treatment adherence (Time frame: within 5 working days of treatment completion) was eliminated as a primary outcome measure
	ACT-smart: Smartphone-supplemented iCBT for Social Phobia and/or Panic Disorder [82]	Current Primary Outcome Measures and Current Secondary Outcome Measures (submitted: November 27, 2013) All outcomes that listed month 24 after the treatment period in the time frame were changed to month 36 after the treatment period.
**Alcohol use disorder**		
	mWELLCARE: An Integrated mHealth System for the Prevention and Care of Chronic Disease (mWELLCARE [134]	Changed September 12, 2016: no longer tracking 10-year risk of CHD^r^ and added tracking for alcohol use, fasting blood sugar, total cholesterol, CVD^s^ risk, and cost
	Project Guard: Reducing Alcohol Misuse/Abuse in the National Guard [88]	Updated August 8, 2016: No changes
	Skills-Training for Reducing Risky Alcohol Use in App Form [137]	Updated October 23, 2018: no longer looking for reduction in alcohol consumption
	Usefulness of Supportive Text Messages in the Treatment of Depressed Alcoholics [96]	Updated December 17, 2011: Becks Depression inventory Score was added, and global assessment of function score was added
**Epilepsy**		
	Embrace: Seizure Characterization [39]	Updated June 22, 2018: Original measures not given
**Schizophrenia**		
	Development of a Mobile System for Self-Management of Schizophrenia (SOS) [60]	Updated June 2, 2016: No longer tracking medication adherence.
	A New Paradigm for Illness Monitoring and Relapse Prevention in Schizophrenia [63]	Updated June 2, 2016: Now using BRPS to assess psychotic symptom severity instead of PANSS^t^
**Other mental and substance use disorders**		
	Preventing HIV/STI in Urban Adolescents via an mHealth Primary Care Intervention [148]	Updated February 12, 2018: No longer tracking Δ in adolescent STI^u^ testing.
	CopeSmart: Using Mobile Technology to Promote Positive Mental Health In Young People [149]	Updated October 10, 2014: Changed to use Emotional Self-Awareness Scale.

^a^Publication automatically indexed to the study by ClinicalTrials.gov identifier (NCT number) without results.

^b^ECG: electrocardiogram.

^c^PHQ-9: Patient Health Questionnaire-9.

^d^QOLI: Quality of Life Inventory.

^e^AAQ: Swiss Agency of Accreditation and quality assurance.

^f^BAI: Beck Anxiety Inventory.

^g^TIC-P: Trimbos and iMTA questionnaire on Costs associated with Psychiatric illness.

^h^BDI: Beck Depression Inventory.

^i^HDRSS: HDRS Hamilton Depressive Rating Scale.

^j^HDRS-17: HDRS Hamilton Depressive Rating Scale.

^k^GAD-7: General Anxiety Disorder-7.

^l^CES-D: Center for Epidemiological Studies-Depression.

^m^HRSD: Hamilton Rating Scale for Depression.

^n^i-CBT: internet-based Cognitive Behavioral Therapy.

^o^SF-36V: Satisfaction Index-Mental Health.

^p^PARS: Pediatric Anxiety Rating Scale.

^q^K-SADS: DSM 5 Diagnosis of Separation Anxiety, Social Anxiety, and Generalized Anxiety Disorder on the K-SADS interview.

^r^CHD: coronary heart disease.

^s^CVD: cardiovascular disease.

^t^PANSS: Positive and Negative Syndrome Scale.

^u^STI: sexually transmitted infection.

The second table shows that the studies were first registered in 2008, with more than half of the studies registered between 2016 and 2018. Across all 135 studies, the mean estimated enrollment was 1078, although the median was only 100. Across all 135 studies, the actual reported enrollment was lower, with a mean of 249 and median of 80. Only about a quarter (35/135, 25.9%) of the 135 studies were NIH funded.

Of the 135 studies included, only 9 (6.7%) studies reported their results. A total of 16.9% (23/135) of studies had publications automatically indexed to the ClinicalTrials.gov identifier (Multimedia Appendix 1). Moreover, 18.5% (25/135) of trials had results reported in some publicly accessible location (either the results section of ClinicalTrials.gov or in publications indexed in databases, which were automatically associated with the ClinicalTrials.gov identifier; Multimedia Appendix 1).

Of those studies that were registered more than 3 years ago (a time frame the authors deemed reasonable given (1) the generally short study interventions, (2) the rapidly changing mHealth landscape, and (3) the 1-year time frame that ClinicalTrials.gov gives to post results after study completion), 14.5% (8/55) of the studies had results posted on ClinicalTrials.gov. The conditions and the number of studies for which the results were published are stroke (1/11), major depressive disorder (4/15), anxiety disorders (2/7), and schizophrenia (1/6). Of the 135 studies, 33 (24.4%) studies that altered their outcomes after the original outcome measures were posted (Multimedia Appendix 2). Of the 135 studies, there were 45 (33.3%) studies that were marked completed by October 2017, 1 year before the search date. Of the 45 studies, 20 (44%) had results reported in some publicly accessible location (either the results section of ClinicalTrials.gov or in publications indexed in databases, which were automatically associated with the ClinicalTrials.gov identifier). Moreover, 15% (7/45) of studies reported their results on ClinicalTrials.gov, and 30% (14/45) of studies had publications automatically indexed to the ClinicalTrials.gov identifier.

There was a statistically significant relationship, as determined by a two-sided *t* test, with studies reporting results having a shorter mean duration (121.7 days) compared with studies never reporting results (153.8 days; *P*<.001). There was also a statistically significant relationship with studies reporting results having lower actual enrollment (142.3 people) compared with studies never reporting results (295.6 people; *P*=.01). There was no statistically significant relationship between the estimated enrollment at the time of study registration and never reporting results. There was also no statistically significant relationship between the status of a study and its estimated or actual enrollment or length of intervention.

## Discussion

### Principal Findings

In this comprehensive analysis of registered mHealth studies of interventions for disabling neuropsychiatric conditions, there were 6 key findings. First, there has been a large increase in the number of clinical trial registrations in the past 2 years; almost half (44.5%) of the trial registrations were registered in the past 2 years. Thus, despite the increasing additions of health-based smartphone apps [1], this snapshot of ClinicalTrials.gov suggests that only a few such apps for high-burden neuropsychiatric conditions are being clinically evaluated in trials. Second, the studies were generally located in the United States, but a few of the studies were funded by the NIH. Third, the study characteristics were such that they would not generally be considered as high-quality evidence and for use in guideline recommendations because of small sample sizes and heterogeneous interventions. Fourth, the mean study duration has not changed with time, suggesting that long-term outcomes are still not the focus of research. Despite the myriad of ways in which results can be reported, a few trials had results reported either as entered on ClinicalTrials.gov or as study results automatically indexed to ClinicalTrials.gov. As stated earlier, only 6.7% (9/135) of such trials that have been registered on ClinicalTrials.gov have reported results. Overall, 18.5% (25/135) of trials had results reported in some capacity (either under the results section of ClinicalTrials.gov or through publications indexed in databases, which were automatically associated with the ClinicalTrials.gov identifier). Fifth, initially specified outcomes were changed after trials commenced in a quarter of all trials. Finally, study duration and sample size of those enrolled are associated with the reporting results of studies.

More trials are being registered on ClinicalTrials.gov, given the requirements for prospective registration as a condition of publication. A previous study examined clinical trial registration for 3 groups of disorders (ie, cardiovascular disorders, mental health disorder, and oncologic disorders) that comprise the largest number of DALYs lost in the United States. The authors found that the number of trials submitted for registration between October 2004 and September 2007 and then October 2007 and September 2010 increased by about 140% from 28,881 to 40,970 [9]. A study assessing trial registration revealed that a few trials still reported on the trial registries [154]. However, more researchers are learning about this requirement, hence the expected increase in trial registrations.

Despite the increase in trial registrations, only 135 trials met the criteria for this study. Thus, a few apps for high-burden neuropsychiatric conditions are being clinically evaluated in the trials reported on ClinicalTrials.gov. A recent study of top-funded digital health companies examined the number of research studies collectively undertaken and found that of the top 10 disease categories examined, 7 were neuropsychiatric. However, none of these industry studies reported on the clinical effectiveness of the digital health tools for these high-burden conditions. Thus, although there is high interest in digital health toward neuropsychiatric conditions, there is little registered evidence that such apps on the market work [155].

A majority of the studies were based in the United States, followed by Europe. This is not because ClinicalTrials.gov is based in the United States, as ClinicalTrials.gov accounts for more than 80% of all the clinical studies in the WHO portal [9]. There may be fewer studies in Europe because of the newly implemented General Data Protection Regulation in 2018, which is a European Union (EU) law for the protection of data and privacy for all individuals within the EU and the European Economic Area. It also concerns the export of personal data outside the EU.

A few (26%) of the trials were NIH funded. This is not surprising as much of the research in digital health has occurred in the private sector. In 2016, 296 private digital health companies received venture funding that totaled to more than US $4.2 billion and approached US $6 billion in 2017 [155]. The 20 top-funded, private US-based digital health companies were studied to analyze their products and services, peer-reviewed evidence, and the potential for impact on patients with high-burden conditions. Less than one-third (27.9%) of the studies targeted patients with a high-burden condition. Only 16 (15%) studies assessed the clinical effectiveness of the product or service, and only 8 (8%) studies assessed the clinical effectiveness of the product or service in a high-burden or high-risk factor population. Only a small number of studies published data, and interestingly, journals without impact factors were the most common (31%) source of publications [155].

There was tremendous heterogeneity in the purpose of the use of the apps not only across conditions but also within a given condition. The most common purpose across all conditions was symptom tracking and medication adherence. There were also some studies based on skills learning, for example, cognitive behavioral therapy or progressive muscle relaxation, and an app was used to help with the delivery of these skills. A small number of studies were designed to detect physical symptoms or signs, such as atrial fibrillation and seizure, or enhance communication with clinicians using apps connected to sensors and/or devices. This latter intervention was to provide support to patients, in some cases with the intent to prevent relapse or indicate the degree of symptoms to potentially lead to a change in medication management if warranted.

In terms of study design, the studies were generally small (samples sizes<100). This study’s results were comparable with the study examining the ClinicalTrials.gov registration of interventional studies for cardiovascular disease, mental health, and oncology, which found that 62% of the trials had an anticipated enrollment mean of less than 100 [9]. This study’s findings were also comparable with a study of trials conducted by the top 20 funded digital health companies, which found that 51% of the studies had less than 100 participants [155]. Thus, these studies will not be able to help in creating major guidelines with high-quality evidence related to these disabling neuropsychiatric conditions.

Although this study was not designed to assess why there is low registration of trials, the potential reasons may include lack of awareness and different prioritization in the app development industry. “Given the diversity of stakeholders involved in mHealth research, competing outcomes, priorities, funding, and publication requirements may potentially mean some studies are less likely to be registered” [156].

This study’s results are in line with previous work showing that few studies reported published results on ClinicalTrials.gov. In a study examining trial registration compliance in publications related to headache, only 26% of all the studies that should have been registered were indeed registered [154]. A recent study of 556 trial registrations on ClinicalTrials.gov showed that out of all the trials in the study, 150 (27%) trials remained unpublished 5 years after the study completion dates [157]. There are a number of potential reasons for the low publication rates. First, the lack of reporting of negative results is a well-known phenomenon in academic medicine [6,158,159]. For example, some trials of triptans [160] and gabapentin [154,161] were never published. Second, in mHealth, there are likely specific challenges to publication, including high attrition rates, usability issues, and lack of sufficient previous formative research [162]. Third, previous research has shown that clinical trials with large sample sizes were more likely to be published [154,158,162,163]. Many of the mHealth clinical trial registrations in this study’s sample sizes were considerably small.

As noted previously, the study outcomes were changed. In a systematic review in 2011 assessing the transparency of outcome reporting and trial registration of randomized controlled trials and top psychosomatic and behavioral health journals, of the 63 articles meeting the study criteria, only 25 (39.7%) articles had adequately declared primary or secondary outcomes [164]. Thus, this study’s results are in line with previous research in the field of mental health. This is an especially prevalent concern in digital health, where researchers could easily change their outcomes and conduct selective analyses [165].

Finally, this study’s findings suggest that study status is not associated with either estimated or actual sample size or length of the intervention. Study status may not be updated in real time; thus, it may not reflect the true status of the study. This study’s findings that studies never reporting any results have a longer duration than those reporting results are logical in the sense that shorter studies may be easier to complete. Similarly, this study’s findings that studies never reporting any results have a higher mean number of participants compared with studies that did report results make sense, as larger studies are more likely to be difficult to complete. In sum, these findings that shorter and smaller studies are associated with reporting results compared with longer and larger studies are intuitive and reflect that digital health studies have the same challenges that all clinical studies face in terms of reaching reporting status.

### Limitations

One of the limitations of this study is that we may have failed to identify some mHealth studies due to the sampling methodology. The primary search terms that we used were “mHealth,” “smartphone,” “electronic diary,” and “mobile technology,” while specifying subcategories of the different neuropsychiatric conditions (ie, migraine, migraine with aura, migraine without aura, and migraine disorders). Other search terms such as “digital,” “ecological momentary assessment,” “experience sampling,” or “log” may have captured additional studies. We only searched ClinicalTrials.gov, and other trial registration websites exist. Second, many of the studies are recent, and thus authors may not have completed their studies and posted results yet. Third, the potential usefulness of different behavioral interventions administered through smartphone apps is not listed on ClinicalTrials.gov and is likely unknown; it is generally the point of the studies to assess whether these interventions may be effective. Finally, the information on ClinicalTrials.gov will always be incomplete for two main reasons: (1) Individual studies may not be registered in the database. Second, information entered on ClinicalTrials.gov may be incomplete, for example, certain data elements may have had a different format or structure or may have been optional when the study information was initially entered. (2) There are few incentives to motivate responsible parties to update their studies [150] registered on ClinicalTrials.gov. One study found that 17% to 20% of the studies on ClinicalTrials.gov were observational and only 7% had posted results [150,151]. Inferential statistics were limited based on reports from individual studies.

### Future Directions

The study information on ClinicalTrials.gov is helpful for understanding the landscape of smartphone-based studies for neuropsychiatric conditions. However, the information listed does not offer enough detail to fully understand the nature of smartphone apps and sensor data collection. Future solutions may include the posting of web-based demonstrations of apps being studied or links to the version of the app used in trials. In addition, it is clear that many of the health-based apps are being developed by the private sector. Efforts need to be made to encourage commercial companies to register their studies on ClinicalTrials.gov and to adhere to the trial registration guidelines, for example, report trial results in a timely manner, as described by ClinicalTrials.gov. For health-based apps that make claims of efficacy, there needs to be stringent oversight of the registered clinical trials.

### Conclusions

Despite the increasing use of health-based smartphone apps by the general public, only a few such apps are rigorously evaluated in clinical settings. Similar to other research on the studies registered on ClinicalTrials.gov, studies of the top neuropsychiatric conditions involving mHealth, registered on ClinicalTrials.gov, tended to be small, and there was a large amount of heterogeneity in the methods (types of interventions), duration, and reporting methods [9]. Moreover, very few registered studies (6.7%) reported their results, raising the question of whether the burgeoning creation of mHealth-based interventions is efficacious, despite these apps being widely downloaded and used. There were few studies for the most disabling neuropsychiatric conditions that typically use electronic diaries for the self-management of migraine and epilepsy. Future work should focus on studying the efficacy of these mHealth interventions for neuropsychiatric conditions if they are to be used by patients with these disabling conditions. Such studies should be registered on ClinicalTrials.gov to ensure transparency and so that the public can also learn about the research being conducted using these interventions.

## Appendix

Multimedia Appendix 1 Study methodology, key findings, and data collected.

Multimedia Appendix 2 Mobile health studies, study criteria, and altered outcomes.

## Abbreviations

DALYs: disability-adjusted life years
EU: European Union
ICJME: International Committee of Journal Medical Editors
IRB: Institutional Review Board
mHealth: mobile health
NIH: National Institutes of Health
NYU: New York University
SOM: School of Medicine
WHO: World Health Organization
