# Supplementary material for: Characteristics of Neuropsychiatric Mobile Health Trials: Cross-Sectional Analysis of Studies Registered on ClinicalTrials.gov
Source: JMIR Mhealth Uhealth. 2020 Aug 4;8(8):e16180. doi: 10.2196/16180 (PMC7473471; doi:10.2196/16180)
Supplement: Multimedia Appendix 1 [file mhealth_v8i8e16180_app1.doc]

**Supplementary Table 1: Methodology for Determining the Relevant ClinicalTrials.gov Studies**

| Determining The Top Ten Neuropsychiatric Conditions | We identified the top ten most disabling neuropsychiatric conditions worldwide according to the 2016 World Health Organization (WHO) Global Burden of Disease Study, which was the most recent year for which full data were available. We used the Global Burden of Disease results tool to generate the list by selecting DALYs as the measure of disability. Stroke, and neurological disorders, mental disorders, drug use disorders, depressive disorders, eating disorders, and autistic spectrum disorders were selected as the neuropsychiatric categories for all ages and both sexes. This generated a list of 28 conditions and the 2016 DALYs attributable to each. In descending order, the top ten conditions were: 1) Stroke; 2) Migraine; 3) Major Depressive Disorder; 4) Alzheimer’s Disease and other Dementias; 5) Anxiety Disorders; 6) Alcohol Use Disorders; 7) Opioid Use Disorders; 8) Epilepsy; 9) Schizophrenia; and 10) Other Mental and Substance Use Disorders. |
| --- | --- |
| Identification of Relevant mHealth trials on ClinicalTrials.gov | The relevant study variables was determined by a team of three experts in the field: 1. A NIH mHealth Scholar who is a board certified neurologist with dual subspecialty training and certification in Neuropsychiatry-Behavioral Neurology and Headache Medicine; 2. A digital psychiatry expert who is board certified in Psychiatry; and 3. A research methodologist who is an internist with subspecialty certification in Headache Medicine. They confirmed that they were using appropriate variables by conducting a literature search of studies evaluating trials registered on ClinicalTrials.gov. We searched for relevant current or completed mHealth trials for each of the ten conditions on ClinicalTrials.gov from October 3rd, 2018 to November 27th , 2018. For each condition we conducted multiple searches using various terms and synonyms for the disorder in question, combined with the terms mHealth or smartphone. For example, when searching for mHealth trials in migraine, we searched for trials of migraine, migraine with aura, migraine without aura, and migraine disorders. The full search criteria are presented as follows:  -Stroke: Condition selected as mHealth stroke, smartphone stroke, electronic diary stroke and mobile technology stroke.  -Migraine: Condition selected as mHealth migraine, smartphone migraine, electronic diary migraine and mobile technology migraine. It was completed with condition selected as mHealth migraine with aura, smartphone migraine with aura, electronic diary migraine with aura and mobile technology migraine with aura. It was completed again with mHealth migraine disorders, smartphone migraine disorders, electronic diary migraine disorders and mobile technology migraine disorders.  -Major depressive disorder: Condition selected as mHealth major depressive disorder, smartphone major depressive disorder, electronic diary major depressive disorder and mobile technology major depressive disorder. It was completed again with mHealth depression, smartphone depression, electronic diary depression and mobile technology depression. It was then completed again with mHealth clinical depression, smartphone clinical depression, electronic diary clinical depression and mobile technology clinical depression.  -Alzheimer’s disease and other dementias: The search was completed first with mHealth Alzheimer’s disease, smartphone Alzheimer’s disease, electronic diary Alzheimer’s disease and mobile technology Alzheimer’s disease and then mHealth dementia, smartphone dementia, electronic diary dementia and mobile technology Alzheimer’s disease.  -Anxiety disorders: The search was completed with mHealth anxiety disorders, smartphone anxiety disorders, electronic diary anxiety disorders and mobile technology anxiety disorders. It was then completed with mHealth anxiety, smartphone anxiety, electronic diary anxiety and mobile technology anxiety.  -Alcohol use disorders: The search was completed with mHealth alcohol use disorder, smartphone alcohol use disorder, electronic diary alcohol use disorder and mobile technology alcohol use disorder. It was then completed with mHealth alcohol, smartphone alcohol, electronic diary alcohol and mobile technology alcohol.  -Opioid use disorders: The search was completed with mHealth opioid use disorders, smartphone opioid use disorders, electronic diary opioid use disorders and mobile technology opioid use disorders. It was then completed with the criteria of mHealth opioid, smartphone opioid, electronic diary opioid and mobile technology opioid.  Epilepsy: The search was completed with mHealth epilepsy, smartphone epilepsy, electronic diary epilepsy and mobile technology epilepsy. Schizophrenia: The search was completed with mHealth schizophrenia, smartphone schizophrenia, electronic diary schizophrenia and mobile technology schizophrenia. It was then completed with mHealth schizoaffective disorder, smartphone schizoaffective disorder, electronic diary schizoaffective disorder and mobile technology schizoaffective disorder. It was completed again with mHealth psychosis, smartphone psychosis, electronic diary psychosis and mobile technology psychosis.  -Mental and substance abuse disorder: The search was completed with mHealth mental and substance abuse disorder, smartphone mental and substance abuse disorder, electronic diary mental and substance abuse disorder and mobile technology mental and substance abuse disorder. It was then completed with mHealth mental and substance abuse, smartphone mental and substance abuse, electronic diary mental and substance abuse and mobile technology mental and substance abuse.  All components of the disorder were searched in the field of condition or disease. For all searches, the country field was not specified.  We did not limit the search by any date, location, or status restrictions (not yet recruiting, recruiting, enrolling by invitation, active, not recruiting, suspended, terminated, completed, withdrawn, unknown). In the initial search, one study team member conducted the search and abstracted data from ClinicalTrials.gov on half of the neuropsychiatric conditions and the other study team member conducted the search and abstracted data on the other half of the conditions. The search strategy was then expanded, and the two study team members then swapped conditions and served as a second screener of trials they had not initially identified, in order to ensure the accuracy of the search and the completeness of the data abstraction. Of note, during the search, studies were eliminated if they were suspended, terminated or withdrawn. The data utilized in the study was taken exclusively from ClinicalTrials.gov. |

**Supplementary Table 2: Key Findings for the** Various Neuropsychiatric Conditions Studied

| *For vascular and neurodegenerative disorders* | The intended purposes of the stroke interventions were as follows: Five of the studies involved rehabilitation through physical activity and nutrition programs. ^1-5^ Three of the studies were to screen for atrial fibrillation, a physical symptom and sign of stroke. ^6-8^ Two of the studies were to monitor blood pressure. ^9,10^ All stroke interventions were six months or less (Table 2).  The five Alzheimer’s disease and other dementias interventions had varied purposes.  The first intervention was intended to help manage varied psychological and interventional symptoms of dementia. ^11^  The second was intended to delay the process of cognitive impairment and reduce the conversion rates to Alzheimer's disease in patients diagnosed with type 2 diabetes mellitus with mild cognitive impairment. ^12^  The third involved reminders for tasks that needed to be completed. ^13^ The fourth was language training in the form of a game. ^14^ The fifth studied the use of an app to promote cognitive health in older adults. ^15^ The length of the interventions for Alzheimer’s disease and other dementias ranged from 4 weeks to 12 months (Table 2). |
| --- | --- |
| *For chronic conditions with paroxysmal attacks such as migraine and epilepsy* | Three of the migraine interventions involved pain coping skills. ^16-18^ Two interventions ^17,18^ had symptom tracking and medication adherence capability. The fourth migraine study involved an intervention assessing the effect of smartphone-based communication with clinicians on medication prescribing behavior for migraine patients. ^18^ All migraine interventions were six months or less. ^16-19^  There were four epilepsy studies, and there were three different intended purposes. One intervention was a diary tracker, or daily log, to track seizures, medications, and stress and medication adherence tracker with a built-in camera pill scanner. ^20^ Another study had a stress management behavioral intervention (progressive muscle relaxation), based on skills learning. ^21^ The third and fourth studies had devices to identify seizures. ^22,23^ Lengths of the interventions were duration of a hospital visit-6 months. |
| *For mood, anxiety, and other mental health conditions* | For major depressive disorder, twelve of the interventions utilized cognitive behavioral therapy. ^24-35^ Four of the interventions targeted adolescents with a diagnosis of major depressive disorder. ^25,27,33,36^ Two of the interventions utilized mental health telemetry to collect mood journal data. ^37,38^} Three of the interventions were targeted at specific populations e.g Hispanic-Latinos. ^26,39,40^  Two of the interventions were built on group therapy. ^41,42^ All interventions were six months or less (Table 2).  For schizophrenia, five of the studies used a smartphone application to foster self-management of the condition. ^43-47^ One study had an active sensor stream to identify relapse signs in real time to mitigate the severity of relapse and enhance communication between clinician and patient. ^48^ Two of the studies employed techniques of cognitive behavioral therapy. ^49,50^ Two studies focused on behavioral intervention via smartphone and goal setting to facilitate development of social skills and social motivation. ^51,52^ The active comparator for most studies was treatment as usual. The interventions were daily to a duration of three months (Table 2)  For the other mental and substance use disorders, two of the studies used a smartphone application to develop a support system for the patient via texting, assessments, and feedback from providers and communities. ^53,54^ Two studies also facilitated communication with providers through availability of health records and secure mobile platforms. ^55,56^ Two studies also delivered treatment such as behavioral treatment or cognitive stimulation through a smartphone application. ^57,58^ One study focused on improving case management for those who are homeless through the use of a smartphone management program. ^59^ One study used a smartphone application as a mobile journal and replacement for a paper alternative. ^60^ The studies were mostly randomized and open label with one exception of single group assignment. ^55^ The interventions for other mental and substance use disorders ranged from daily to six months (Table 2).  For anxiety disorders, four of the interventions related to preoperative anxiety. ^61-64^ Three of the interventions targeted anxiety in children. ^61,62,65^ Three of the interventions administered cognitive behavioral therapy through a smartphone application. ^65-67^ One of the interventions centered on mindfulness meditation and relaxation. ^64,68^ The interventions for anxiety disorders ranged from daily to up to 5 years (Table 2), |
| *For abuse/misuse disorders such as alcohol and opioids* | The intended purposes of the alcohol related interventions varied as follows: 1. cognitive behavioral therapy/supportive messages; 2. a breathalyzer/self-monitor; 3. cognitive rehabilitation; 4. Text messaging to reduce alcohol use; 5. Social network for alcohol cessation; 6. Educational intervention with components of value-based feedback. Most interventions (21/29) were for three months or less. ^69-89^  Three opioid interventions were directed at self-management and reducing opioid usage. One was an interactive voice learning motivational intervention support system. ^84^ Another was a self-directed educational intervention about addiction and ways to help ^88^ and the third was a self-management tool. ^85^ Another type of opioid intervention monitored analgesia consumption and disposal. ^86^ The interventions were generally short, with one being seven days ^86^ and another being one month ^85^. |

**Supplementary Table 3: Data Abstracted from ClinicalTrials.gov**

| **STROKE** | | | | | | | | | | |
| --- | --- | --- | --- | --- | --- | --- | --- | --- | --- | --- |
| **Title, Sponsors and Collaborators, Recruitment Status, Location, Investigator, ClinicalTrials.gov Identifier** | **First Posted, Results First Posted, Last Update Posted,** | **Phase, Study Type, Allocation, Masking, Estimated Enrollment, Actual Enrollment** | **Intervention** | **Eligibility Criteria: Inclusion/ Exclusion** | **Primary and Secondary Outcomes** | **Reporting Groups: Description** | **Outcome Measures:** | **Measures of Adherence: Planned & Posted** | **Reporting of Clinical Trials Outcome** | **Altered Outcomes** |
| **Clinical Effect Size of an Educational Intervention in the Home and Compliance on People Who Suffer From Stroke** ^90^  University of Malaga  Recruiting  Malaga, Spain  Antonio I Cuesta Vargas, PhD  NCT01980641 | November 11, 2013  N/A  May 16, 2017 | N/A  Interventional (Clinical Trial)  Randomized  Single (Investigator)  80  40 | Educational intervention that assesses the effect of teaching through active participation in learning via a smartphone application.  No Intervention: Control Group  Assessment of each participant’s home + performance on the ADL.  Experimental Group:  Assessment of each participant’s home and performance on ADL. Therapist provides list of advice related to HTAS items which are negatively evaluated. Additionally, educational advice provided by therapist to improve adherence to treatment.  Experimental Group: Application smartphone-based group- Participants receive reminders of the advice given by the therapist through a smartphone application.  No intervention: Non-application smartphone-based group  No reminder on smartphone application. | Acute Stroke (<3 months since discharge to their homes  Score from 20-50 on Barthel Index  Score from 35-75 on Functional Independence Measure  Severe cognitive impairment  Not able to walk 3 meters w/o physical assistance  Inability to stand more than 30 seconds w/o physical support  Communication or comprehension problems  Surgical procedure in lower limbs 12 months before recruitment  Secondary neurological pathology  Severe cardiovascular, respiratory, orthopedic or metabolic problems | Barthel Index (Baseline to 2, 4, 12 and 22 weeks)  Canadian Neurological Scale (Δ from Baseline to 2, 4, 12 and 22 weeks), Stroke Impact Scale, Mini Mental State Examination, Functional Independence Measure, Trunk Control Test, Modified Rankin Handicap Score, Escala de Apoyo Social Percibido, Escala de Calidad de Vida para el Ictus, Functional Reach Test, Romberg Test, Time Up and go, Strength testing w/ portable dynamometer. Timed-stands test | N/A | N/A | N/A | *1 Abstract Submitted | N/A |
| **The Adherence and Knowledge Exchange Heart and Stroke Medicines Guide (TAKEmeds)** ^91^  Duke Kunshan University  Completed  Haikou, Hainan, China, 570100  JD Schwalm, MD Population Health Research Institute, Hamilton Health Sciences Corporation, Canada  [NCT02597205](https://clinicaltrials.gov/show/NCT02597205) | November 5, 2015  N/A  August 27, 2018 | N/A  Interventional (Clinical Trial)  N/A  None (Open Label)  N/A  190 participants | Increase use of evidence-based, secondary-prevention medications + promote healthy lifestyles among myocardial infarction (MI) patients using provider-facing mobile app + patients-facing text messages.  Experimental: mobile app  Multi-faceted intervention for physicians and patients. Physician-facing app and patient-facing messages.  Behavioral: physician-facing app and patient-facing messages  Physician-facing app designed to help physicians w/ recruiting and managing patients and w/ evidence based medications prescription. Messages sent to patients to improve medical adherence and modify lifestyle. | 18+  Physicians who 1) manage MI patients 2) physical + mentally able to manage MI conditions  Patients who 1) suffered from myocardial infarction 2) physical + mentally able to manage MI conditions  Physicians who do not own a smartphone, do not manage MI patients, current participating in another study  Patients not able to use smartphone, severely ill, current participating in another study | Medical Adherence (Time Frame: 3 months)  Score change measured by 4-item Morisky Green Levine Scale  Change in diet (Time Frame: 3 months)  Measured by the BRFSS: Fruit and Vegetable Module  Change in exercise (Time Frame: 3 months)  Measured by the International Physical Activity Questionnaire  Change in smoking habit (Time: 3 months)  Measured by Tobacco Questions for Surveys, World Health Organization | N/A | N/A | N/A | N/A | N/A |
| **Impact of M-health based Intervention on Adherence to Healthy Physical Activity After Stroke** ^1^  Consorci Sanitari de Terassa  Recruiting  Teressasa, Barcelona, Spain  Montserrat Grau-Pellicer,  Consorci Sanitari de Terassa  NCT03507894 | April 25, 2018  N/A  April 26, 2018 | Not applicable  Observational  Not applicable  Not applicable  58 participants  Not applicable | 8-week multimodal exercise rehabilitation program (MERP)  Aim of MERP=improve walking speed, walking ability, activities of daily living and quality of life among people who have suffered a stroke.  Experimental: m-health stroke rehabilitation  8-week multimodal exercise rehabilitation (MERP) of aerobic exercise, task oriented activities, balance and stretching exercises + mobile app technology  Control: Behavioral: Multimodal Exercise Rehabilitation Program  Rehabilitation program of aerobic exercise, task oriented activities, balance and stretching exercises + mobile app technology. | Diagnosis of ischemic or hemorrhagic stroke  Age ≥18  Functional Ambulation Classification (FAC) ≥3  Barthel Index ≥45  Cognitive impairment (Mini Mental State Examination ≤24)  Unstable cardiovascular disease (acute heart failure, recent myocardial infarction, unstable angina and uncontrolled arrhythmias)  Alcohol or other toxic substances abuse  Decompensated psychiatric disorders prevented from following a group session | Adherence (Time Frame: 8 weeks)  Monitored with an app that will record activity time of walking time and distance.  Gait Speed (Time Frame: 8 weeks)  Measured with the Ten Meter Walking Test (10MWT). Participants given 2 meter warm up distance for walking before 10m distance and 2 m beyond the 10 m. Time that takes to walk 10 m at comfortable pace + maximum speed will be registered in m/sec. | N/A | N/A | N/A | N/A | N/A |
| **Hispanic Secondary Stroke Prevention Initiative (HISSPI)** ^9^  University of Miami  National Institute on Minority Health and Health Disparities (NIMHD)  Recruiting  Miami, FL, USA 33136  Olveen Carrasquillo, MD, MPH  NCT02251834 | September 29, 2014  N/A  May 16, 2018 | Not applicable  Interventional (clinical trial)  Randomized  Single (Investigator)  300 participants  N/A | Study using Community Health Workers (CHW) and mobile technologies using cell phones to reduce the risks for a recurrent stroke among Latino stroke patients. Effectiveness of a combined multilevel intervention consisting of Community Health Workers (CHW) and mobile based phone technologies in lowering of systolic blood pressure (SBP), most important risk factor for recurrent stroke.  Experimental: Community Health Worker (CHW):  Home visits, phone based contacts, group level activities, mobile technology. 4 home visits and 10 phone calls during months 3-12. Group visits 2x/month. Highly individualized and vary by patient.  Usual Care: Usual care and health education brochures every 4 months  Patients randomized to control group will receive usual care. | 18+  History of ischemic or intra-cerebral hemorrhagic stroke within past 5 years  Be Hispanic/Latino on self-report  mRs≤ 3  Reside in Miami-Dade County  Any life threatening morbidity including active cancer diagnosis  Enrollment in other non-acute stroke, cardiovascular, diabetes study  Patients w/ arm circumference ≥47 cm | Blood Pressure (Time Frame: 1 year)  Primary hypothesis is at 12 months, patients in intervention arm will have SBP on average, 8mmHg lower than those in the control group.  LDL (Time Frame: 1 year)  LDL-C level of 70 mg/dL associated w/ 28% stroke risk reduction and stroke patients with a 50% reduction in LDL-C had a 35% reduction in combined risk of nonfatal and fatal stroke.  Self-reported adherence to statins and anti-platelet medications (Time Frame: 1 year)  Morisky medication adherence scale  A1C (Time frame: 1 year)  Among patients with diabetes, measure A1C | N/A | N/A | N/A | *1 Abstract Submitted | N/A |
| **Increasing Physical Activity in Stroke Survivors Using STARFISH** ^2^  University of Glasgow  Chest, Heart and Stroke Association Scotland  NHS Greater Glasgow and Clyde  National Health Service Ayrshire and Arran  National Health Service Lanarkshire  Recruiting  Glasgow, Scotland  Dr. Lorna Paul, University of Glasgow  NCT02494245 | July 10, 2015  N/A  June 1, 2017 | N/A  Interventional (Clinical Trial)  (clinical trial)  Randomized  Single (Outcomes Assessor)  128 participants  N/A | Compare physical activity in stroke survivors who complete a 4-month physical activity intervention using STARFISH application to control group receiving 4 months of TAU.  Intervention Group: 128 participants in a 4-month physical activity intervention using STARFISH app w/ aim of increasing physical activity by 3000 steps/day. Participants work in groups of 4 but each participant will have own individualized step count target based on baseline step count. Participant reaches step count target on at least 5 days of the week, target increased by 5% for following week, up to max increase of 3000 steps above baseline.  Control Group: Given a booklet with general advice on physical activity. | Single unilateral stroke  Discharged from active rehabilitation  Able to walk independently, w/ or w/o using an aid or orthosis  History of serious cardiac disease in the previous 6 months  Uncontrolled blood pressure  Significant neurological or musculoskeletal conditions in addition to stroke  Currently participating in another rehabilitation or pharmacological clinical trial | Δin the # of mean steps per day (Baseline, 4 months, 6 months) -Measured with an ActivPAL activity monitor from PAL Technologies in Glasgow, Scotland  Δin sedentary time (Baseline, 4 months, 6 months), Δ in the six minute walk test, Δ in 10 meter walking test, Δ in the Nottingham Extended, activities of Daily Living Scale, Δ in Fatigue Severity Scale , Δ in Stroke Specific Quality of Life Scale, Δ in the Hospital, anxiety and Depression, Δ in blood pressure, Δ in weight, Δ in plasma lipid profile, Δ in heart rate, Δ in walking time, Δ in walking intensity, liver function, Δ in C-reactive protein level, Δin HBA1c | N/A | N/A | N/A | +1 Paper  Submitted | N/A |
| **Effects of Patient -centered Stroke Educating System: A Randomized Control Group**  Taipei Medical University  Completed  Taipei, Taiwan  Wen-Hsuan Hou, PhD  NCT02591511 | October 29, 2015  N/A  September 14, 2018 | Not applicable  Interventional (Clinical Trial)  (clinical trial)  Randomized  Single (Investigator)  80  63 | Establish Patient-Center Computerized Educating System for Stroke (PACESS) for patients with lack of knowledge about stroke.  Experimental: health-education app, PACCESS  Smartphone + pad=stroke related health education app intervention group  Active Comparator: Traditional health education manual.  Stroke-related health education manual control group. | First time diagnosis of stroke and cerebral infarction  Designated caregiver who participates in the surveys  >6 years of education  Download smartphone application on smartphone  Cognitive impairment  Having major mental health illnesses  Inability to read/answer questionnaires | Stroke Health Education Knowledge Questionnaire (4 weeks)-Single choice questions based on stroke health education manual. 12 risk factors. Each risk factor is comprised of 3 questions for a total of 36 questions. Higher score represents better outcome. | Experimental Group  Active Comparator Group | N=30 in intervention with avg score on Stroke Health Education Knowledge Questionnaire 29.07 SD+5.27, N=33 in comparator arm with avg score 28.00 SD+5.46.  (No statistical analysis provided) | N/A | N/A | Original Primary Outcome Measures (submitted: October 28, 2015): Stroke Health Education Knowledge (Time Frame: 2 weeks) Changed August 16, 2018: Stroke Health Education Knowledge Questionnaire (Time Frame: 4 weeks) |
| **The Stroke and Exercise Program** ^3^  The Miriam Hospital  Recruiting  Rhode Island, USA  Dale S Bond, Ph.D  [NCT02701998](https://clinicaltrials.gov/show/NCT02701998) | March 8, 2016  N/A  March 22, 2017 | N/A  Interventional (Clinical Trial) (clinical trial)  Randomized  None (open label)  70 participants  N/A | Mobile health (mHealth) enhanced physical activity (PA) intervention to increase daily bout-related and total moderate-intensity PA + reduce (SB) in non-physically impaired patients w/ ischemic stroke or TIA.  Experimental: mHealth-Enhance Physical Activity Intervention (PAI)  Tech PAI participants increase average baseline daily walking exercise by 30 mins, increase average baseline daily steps by 4000 at week 12, get up and walk for at least 2 mins after 60 mins of sitting. Activity tracker (Tech-PAI) that provides real-time monitoring of steps and issues text-based prompt after 60 mins of sitting to get up and move. Weekly email feedback message from staff on goal progress + view internet-based video lessons provide behavioral strategies to increase MPA + steps and decrease SB in first 6 weeks of intervention period  Active Comparator: Physical Activity Intervention  PAI participants same goals as Tech-PAI intervention participants. Receive pedometer + print materials to assist in recording and increasing daily steps and bout-related MPA. But no activity tracker, video-based skills training lessons or weekly feedback. | Admitted to hospital with diagnosis of stroke  mRS of 0 or 1  No major signs or symptoms indicating cardiovascular, pulmonary or metabolic disease  Able to successfully complete a submaximal graded exercise test at baseline  Participating in other research study or structured intervention  Any condition that would like reduce adherence to study protocol | Feasibility and acceptability of a mHealth enhanced PA intervention (End of 12-week intervention)-Measured using a questionnaire.  Δ in daily bout-related and total moderate physical activity (Baseline, Midpoint of 12-week intervention, end of 12-week intervention)-Objectively assessed via a multi-sensor monitor.  Δ in daily percentage of waking hours spent in sedentary time (Baseline, Midpoint of 12-week intervention, end of 12-week intervention)-Objectively assessed via a multi-sensor monitor.  Δ in performance-based measure of cardiorespiratory functioning (Baseline, Midpoint of 12-week intervention, End of 12-week intervention), Δ in performance-based measure of physical functioning, Δ in performance-based measure of cognitive function, Δ in stroke-related impact, Δ in health-related quality of life, Δ in C-reactive protein | N/A | N/A | N/A | N/A | N/A |
| **mHealth Screening to Prevent Strokes (mSToPS)** ^6^  Scripps Translational Science Institute  Janssen Science Affairs, LLC  Aetna, Inc  Active, not recruiting  La Jolla, California, USA  Steven R Steinhubl  NCT02506244 | July 23, 2015  N/A  July 6, 2018 | N/A  Interventional (Clinical Trial) (clinical trial)  Randomized  None (open label)  2274 participants  N/A | Screening for asymptomatic atrial fibrillation. Mobile health technology enabled home monitoring program to document previously undiagnosed atrial fibrillation to find outcomes benefit associated with early detection. Intermittent monitoring for individuals without prior history of atrial fibrillation but at risk based on clinical risk factors.  Experimental: Immediate Monitoring  Receive ZIO XT patch sensor. Wear it for the first and last 2-week period of the 4-month monitoring period of time 0 to 4 months.  Active Comparator: Delayed Monitoring  Usual care for 4 months after which they will receive the ZIO XT patch sensor and wear it for the first and last 2-week period of study months 4 through 8. | Participants: Study population derived from the Aetna or Medicare populations.  Males or females >75 or male age> 55, or female age >65 and prior stroke or heart failure or diagnosis of diabetes and hypertension or mitral valve disease or left ventricular hypertrophy or COPD requiring home O2 or sleep apnea or history of pulmonary embolism or history of myocardial infarction or diagnosis of obesity  Current or prior diagnosis of atrial fibrillation, atrial flutter or atrial tachycardia  Receiving chronic anticoagulation therapy  Hospice care/limited life expectancy  End stage renal disease  moderate or greater dementia  Implantable pacemaker and/or defibrillator  History of skin allergies to adhesive patches | Incidence of newly diagnosed AF (  End of 4-month monitoring period)-Defined by at least 30 seconds of AF at the end of the 4-month monitoring period vs. the delayed monitoring cohort (primary) and observational control (secondary).  Prevalence of atrial fibrillation (1 year)  Time to first event of the combined endpoint of stroke, systemic emboli, or MI in patients diagnosed with AF in monitored vs. control cohorts (3 years)  Difference in total healthcare costs in AF cohorts of monitored and controls (3 years). | N/A | N/A | N/A | N/A | N/A |
| **My Stroke Team (MYST): Stroke App Pilot Study** ^92^  McMaster University  Recruiting  Hamilton, Ontario, Canada  Maureen Markle Reid, RN, PhD  NCT02230280 | September 3, 2014  N/A  May 18, 2017 | N/A  Interventional (Clinical Trial)  N/A  None (open label)  30 participants  N/A | Improve the overall quality of stroke care at home. Explore the feasibility and acceptability of this mobile tool, determine impact on usability for home care providers, stroke survivors and their family caregivers, determine impact on the costs of use of health services.  Arm: The Intervention Cohort  Community transition + rehabilitation intervention that includes a mobile health solution. Participants offered regular in-home visits over the 6-month study period in addition to usual outpatient rehabilitation services. Health care providers will use a mobile health application (MYST) to share real time information, access community guidelines and best practice guidelines to improve stroke care at home. | >55 y/o  Newly referred for outpatient rehabilitation w/ confirmed diagnosis of stroke (first ever or recurrent) in prior12 months, not planning to move away from the community in next 6 months, have at least 2 predetermined comorbid conditions.  Unable to read and understand English and do not have access to a translator | Δ in health-related quality of life (HRQoL) for both stroke survivors and caregivers as measured by the SF-12 (Baseline and the end of the intervention (6 months after baseline)-Administered to stroke survivors and caregivers to measure health-related quality of life.  Feasibility of the mobile application as part of the intervention (the end of the intervention (6 months))-Participants viewpoint on the feasibility of the mobile application through focus groups (HCPs) and interviews (stroke survivors and family caregivers)  Δ in the degree of physical functioning related to stroke (Baseline and the end of the intervention (6 months from baseline))  Δ in the prevalence and severity of depression symptoms in stroke survivors and family caregivers  Δ in the degree of strain of family caregiver  Δ in prevalence and severity of anxiety Δ in the self-efficacy of stroke survivors  Δ in the community integration of stroke patients  Δ in the costs of use of health services by stroke survivors and family caregivers  Δ in team functioning (at three months into the intervention study and upon completion of the study (16 months))  Δ in level of integration between home care providers | N/A | N/A | N/A | N/A | N/A |
| **Stroke Inpatient Rehabilitation Reinforcement of ACTivity** ^4^  University of California, Los Angeles  National Taiwan University Hospital  Washington University School of Medicine  University of Vigo  Morinomiya Hospital, Osaka, Japan  Mayo Clinic  IRCCS San Camillo, Venezia, Italy  Fairlawn Hospital, Worcester, MA, USA  Chonnam National University Hospital  Ain Shams University  MedStar National Rehabilitation Network  St. Luke's Hospital, Pennsylvania  Father Muller Medical College  Burke Rehabilitation Hospital  Burwood Hospital, Christchurch, New Zealand  Gazi University  University of Ibadan  Rehabilitation Hospital, Barcelona, Spain  IRCCS San Raffaele  Completed  Massachusetts, USA, Minnesota, USA, Missouri, USA, New York, USA, Pennsylvania, USA, Egypt, India, Ireland, Italy, Japan, Republic of Korea, New Zealand, Nigeria, Spain, Taiwan  Turkey  Bruce H. Dobkin, MD, University of California, Los Angeles  NCT01246882 | November 23, 2010  N/A  May 10, 2017 | N/A  Interventional (clinical trial)  Randomized  Single (Outcomes Assessor)  N/A  140 participants | Determine the effects of daily feedback about physical activity (# of bouts of walking, duration of bouts, total walking distance, average and fasted walking speed) and walking average speed vs. feedback about walking speed only on walking related outcomes in inpatient rehabilitation for stroke.  Daily walking + other exercise monitored by bilateral triaxial accelerometers on the ankles. Activity-recognition algorithms analyze inpatient sensor data and summarize participants at each site.  3 sets of triaxial accelerometers mailed to each site’s coordinator. Worn when they woke up and removed when went to sleep.  Experimental: Augmented activity feedback  Feedback 3x/week about 10-m walking speed + amount and types of physical activity measured using wireless bilateral ankle sensors that measure bouts of walking and cycling speed, duration and distance.  Behavioral: Augmented activity feedback  Feedback about walking speed + amount of physical activity 3x/week from data from wireless sensors on each ankle.  Active Comparator: Speed-only feedback  Feedback 3x/week about over ground walking speed >10m.  Behavioral: Speed-only feedback  Feedback about walking speed 3x/week. | Admission for acute inpatient rehabilitation of first stroke or 2^nd^ stroke after full recovery from prior TIA/Stroke  Time from onset of stroke to admission for rehabilitation <35 days  Stroke from any cause that includes unilateral hemiparesis.  Independent in mobility prior to admission by the Barthel Index  Able to walk w/ no more than physical assistance of 2 persons for at least 5 steps. Subjects can use any type of assistive device and brace needed.  Current medical disease that will limit physical therapy at time of randomization or limited walking prior to stroke.  Aphasia w/ inability to follow 2 step directions during therapeutic instructions or answers yes/no to questions w/ <75% accuracy related to personal health and symptoms | Gait Speed (Time Frame: Discharge)  Distance walked in 3 minutes (Time Frame: Discharge) | N/A | N/A | N/A | +1 Paper Submitted | N/A |
| **iADAPTS to Support Strategy Training After Stroke** ^93^  University of Pittsburgh  Recruiting  Pittsburgh, Pennsylvania, United States, 15260  Elizabeth R. Skidmore, PhD, OTR/L  NCT03253601 | August 18, 2017  N/A  June 25, 2018 | N/A  Interventional (Clinical Trial) (clinical trial)  Randomized  Single (outcomes assessor)  30 participants  N/A | iADAPTS mobile application to support adherence to Strategy Training, a rehabilitation intervention/meta-cognitive instruction that promotes goal setting, planning and self-monitoring after stroke.  Experimental: Strategy Training: iADAPTS Application- iADAPTS mobile health application to supplement in-person and remote intervention sessions.  Active Comparator: Strategy Training: Workbook  Use a printed workbook in addition to in-person and remote by telephone intervention sessions. | 18-105 years  Diagnosis of stroke  Prior exposure to Strategy Training  Vision impairment that impairs ability to read  Poor self-awareness  Current major depressive, bipolar or psychotic disorder  Current alcohol or drug abuse  Severe aphasia  Current diagnosis of dementia, neurogenetic disease or cancer  Anticipated discharge to a skilled nursing facility | Adherence: Observed Client Behavior (Baseline to 3 months)- # of completed activity trials in the iADAPTS application or Workbook.  Adherence: Client-reported progress on goals (Baseline and 3 months)-Measured using the Canadian Occupational Performance Measure.  Adherence: Client perspectives on treatment environment (Baseline and 3 months) | N/A | N/A | N/A | N/A | N/A |
| **Focus Group Study of Lifelong Food and Nutrition Assistance (LIFANA) in Stroke Patients and Caregivers**  ^5^  Cereneo Institute for Interdisciplinary Research (cefir)  Enrolling by invitation  Vitznau, LU, Switzerland, 6354  Krizia Ferrini, Nutrition Specialist  [NCT03635476](https://clinicaltrials.gov/show/NCT03635476) | August 17, 2018  N/A  August 17, 2018 | N/A  Observational  N/A  N/A  8 participants  N/A | Develop and evaluate the LIFANA Nutrition Solution to support healthy nutrition. Include general preferences + automated and personalized nutritional recommendations based on advice by professional nutritionists and allergists. Focus group of 8 participants lasts 2 hours.  Other: Qualitative interview study  Group part of research program aimed at developing and evaluating the LIFANA Nutrition Solution, a meal planning recommender system, which suggests meals based on the user’s personal profile. Automated and personalized nutritional recommendations based on advice provided by professional nutritionists and allergists. | Stroke patients undergoing rehabilitation  Caregivers of stroke patients  Severe aphasia  Severe cognitive deficits | User Needs (Time Frame: 1 day/session)  Explore the perspectives of patients through appropriate adjustments of focus group questions on user needs for managing nutritional status. | N/A | N/A | N/A | N/A | N/A |
| **VR-3D Movie-based education** ^7^  Medical University of Warsaw  Completed  Warsaw, Poland, 02-097  Pawel Balsam, MD, PhD,  NCT03104231 | April 7, 2017  N/A  June 8, 2017 | Interventional (clinical trial)  Single Group Assignment  N/A  None (Open Label)  N/A  100 participants | Virtual reality (VR) three-dimensional (3D) movie. Usage of oral anticoagulation (OAC) to prevent incidence of thromboembolic events. Aim of the OCULUS study to examine whether the 3D movie-based knowledge transfer is effective in teaching patients about the consequences of AF and pharmacological possibilities in reducing risk of stroke.  Experimental: study group  All patients will be shown three-dimensional (3D) movie.  Other: three-dimensional (3D) movie  Brief VR-3d will be shown using the oculus glasses and a smartphone. Then, patients will be asked several questions from the prepared questionnaire. | 18+ y/o  Previously diagnosed dementia | Assessment of the effectiveness of the 3D-movie based knowledge transfer in teaching patients about the consequences of AF (Time Frame: 1 year)  Outcome assessed by questionnaire designed by authors of study, enclosing questions about sex, age, education, current job, AF history and knowledge about consequences of AF and possibility of stroke prevention due to the OAC therapy | N/A | N/A | N/A | +1 Paper Submitted | N/A |
| **Improving Medication Adherence Through SMS (Short Messaging Service) in Adult Stroke Patients: A Randomized Controlled Behavior Intervention Trial**  ^94^  Aga Khan University  Completed  Karachi, Pakistan, 74800  Ayeesha K Kamal, MBBS  [NCT01986023](https://clinicaltrials.gov/show/NCT01986023) | November 18, 2013  N/A  August 19, 2014 | N/A  Interventional (Clinical Trial) (clinical trial)  Randomized  Double (Investigator, Outcomes assessor)  N/A  200 participants | Non-pharmacologic behavioral study to encourage adherence to medications in stroke survivors by tailored and specific SMS reminders to remind stroke survivors to take medication as prescribed and on time.  Experimental: SMS Short Message Service Arm plus Prescription  Drug reminder SMS customized to their stroke prescription. Participants answer back in Yes/No format. Behavior change SMS will be sent twice weekly. Encouraged to take medication using a taxonomy of behavioral change intervention techniques.  No Intervention: Standard Prescriptions and Counselling  Undergo standard treatment and education regarding medication as per standard of care. Standard written prescription and no SMS. | >18 years  Diagnosed w/ stroke at least one month ago on neuroimaging (CT or MRI)  Taking 1+ drug for risk factor control  Personal mobile phone and able to operate SMS  Modified Rankin scores >3  Do not intend to travel outside the country in the next 2 months  Chronic renal failure  Any known malignancy  Enrolled in another study | Medication Adherence Score (2 months)-Measurement of their medication adherence scores after 2 months of follow up on the Morisky Medication Adherence Questionnaire.  Patient Satisfaction with Intervention (2 months)  Acceptability of m-Health Intervention (2 months) | N/A | N/A | N/A | +1 Paper Submitted  * 1 Paper Submitted | N/A |
| **Phone-based Intervention Under Nurse Guidance After Stroke**  ^10^  Medical University of South Carolina  Kwame Nkrumah University of Science and Technology  Completed  Kumasi, Ghana  N/A  NCT02568137 | October 5, 2015  N/A  October 2, 2017 | N/A  Interventional (Clinical Trial)  Randomized  Single (Outcomes assessor)  N/A  60 participants | Mobile phone texting and home blood pressure monitoring directed by trained nurses, to improve patient adherence to proven medical therapies for treating hypertension.  Experimental: Behavioral  Nurse-directed mobile health technology using smartphones to promote adherence to antihypertensive medication.  No intervention: TAU | >18 years; male or female  Index stroke symptoms began no greater than 1 month before enrollment  Uncontrolled hypertension based upon last inpatient or outpatient encounter clinic within previous 12 months  Severe cognitive impairment/dementia  Severe global disability  Renal dialysis; awaiting renal transplant or transplant recipient  Cancer diagnosis or treatment in last 2 years  Planned pregnancy  Vulnerable populations | Recruitment rates (9 months)  Patient Satisfaction Scales (9 months)  Clinic based Blood Pressure (9 months)  Retention rates (9 months)  Med Possession Ratio (9 months)  Morisky Med Adherence Scale (9 months)  Provider Satisfaction Scales (9 months) | N/A | N/A | N/A | +2 Papers Submitted  *1 Paper Submitted | N/A |
| **TeleRehab for Stroke Patients Using Mobile Technology**  ^95^  Ottawa Hospital Research Institute  Completed  Ottawa, Ontario, Canada  Karen Mallet, M.H.Sc  [NCT02615132](https://clinicaltrials.gov/show/NCT02615132) | November 26, 2015  N/A  September 25, 2017 | N/A  Interventional (clinical trial)  Randomized  None (Open Label)  N/A  20 participants | Post-stroke communication deficits (PSCD)=common symptom of patients having sustained a stroke. Deficits include difficulty to produce or understand language, motor speech disorders and cognitive communication disorders. Investigators’ objective is to test the value of providing a mobile platform based Speech Language Therapy (SLT) program to patients discharged from an acute care hospital with stroke and PCSD and awaiting outpatient rehab services vs. standard of care treatment. Investigators will offer iPad-based SLT/standard of treatment to a convenience sample of 20 patients with post stroke communication deficits.  Experimental: Treatment Group  Study SLP will instruct the patient to use iPad apps as intervention for at least 1 hour/day, until they are admitted to outpatient services or for a maximum of 8 weeks. Throughout the telemedicine treatment phase, patients progress will be monitored remotely by a study SLP through Apps/Skype/Facetime/Telephone consultation on a weekly basis.  No Intervention: Control  Patients will be sent home with standard of care | 18+ y/o  Patients w/ diagnosis of stroke being discharged from the Neurology unit and/or the Neurology Acute Care Unit in the Civic Campus of TOH  Overall mild to moderate communication deficits and/or  score ≥ 1 on the best language and/or dysarthria parameters of the National Institute of Health Stroke Score (NIHSS)  Stroke patients being discharged to their home/primary residence awaiting outpatient speech and language therapy services and/or patients being discharged to their home/primary residence who would benefit from SLP therapy services but are unable to receive these secondary to various accessibility challenges  Patients who have access to Wifi connection at their home/primary residence  Patients with pre-existing speech, language disorders or cognitive disorders  Patients w/ severe debilitating diseases that will not be able to perform the required tasks of the study  Patients with severe comprehension deficits  Patients not having access to Wifi connection at home/primary residence  Patients who will be accessing the services of a private SLP while awaiting outpatient rehab | Feasibility (Recruitment and Adherence Rates) (Time Frame: 18 months)  1.Total number of patients enrolled  2.Re-recruitment rate (number of patients enrolled/total number of patients admitted with stroke within the same time period)  3. Proportional benefit (proportion of the discharged PSCD population that can benefit from this intervention)  4. Adherence rate=total number of patients who completed the full course of intervention/total number of patients enrolled. Measure will determine whether discharged PSCD patients can actually tolerate and complete the therapy.  Potential Improvement (Time Frame: 18 months)  Measuring any potential improvements in communication by comparing pre and post-intervention SLP assessments. Latter performed by a blinded study SLP. | N/A | N/A | N/A | N/A | N/A |
| **Empowerment and Mobile Technology in the Control of Cardiovascular Risk Factors in Patients with Ischemic Stroke (CARDIOSTROKE)**  ^8^  Jukka Putaala, Hospital District of Helsinki and Uusimaa  Recruiting  Helsinski, Finland 00290  Tuomo Nieminen, Professor  NCT03710902 | October 18, 2018  N/A  October 19, 2018 | N/A  Interventional (clinical trial)  Randomized  Single (Outcomes Assessor)  405 participants  N/A | CARDIOSTROKE is a randomized trial comparing mobile device assisted control of hypertension w/ screening of occult AF to standard care in patients with recent ischemic stroke or transient ischemic attack.  Experimental: Intervention  Diagnostic Test: ECG monitoring for 3 weeks to detect occult AF  Other: Self-Monitoring of BP and self-titration of antihypertensive medication  1 week monthly self-monitoring of BP and self-titration of hypertensive medication according a prespecified protocol, assisted with a mobile device application.  No Intervention: Control  Standard diagnostic work up, follow up and treatment of hypertension. | Ischemic stroke or transient ischemic stroke (ABCD2 score ≥3)  Aged ≥40 years  Known high risk of cardio embolism  Known indication for anticoagulation  Noncompliance to study interventions  Serious condition hampering study conduct | Number of Participants with New Atrial Fibrillation (Time Frame: 12 months)-new diagnosis if atrial fibrillation (>30s)  Change in Blood Pressure (Time Frame: 12 months)-mean change in systolic/diastolic blood pressure.  Number of Participants with New Cardiovascular Events within 12 Months (Time Frame: 12 months)-any of stroke, myocardial infarction, revascularization or cardiovascular death  Number of Participants with New Cardiovascular Events within 36 months (Time Frame: 36 months)  Health Care Costs (Time Frame: 36 months)-total direct healthcare costs | N/A | N/A | N/A | N/A | Original Primary Outcome Measures (submitted: October 17, 2018) was Atrial Fibrillation (3 weeks) as detected in a 3 week ECG monitoring period and Systolic/diastolic blood pressure (Time Frame: 12 months) measured as difference in systolic/diastolic blood pressure.  Current Primary Outcome Measures (submitted: October 18, 2018) are Number of Participants with New Atrial Fibrillation and Change in Blood Pressure.  Current Secondary Outcome Measures (submitted: October 18, 2018)-Number of participants was added before new cardiovascular events within 12 months and new cardiovascular events within 36 months. |

| **MIGRAINE** | | | | | | | | | |
| --- | --- | --- | --- | --- | --- | --- | --- | --- | --- |
| **Title, Sponsors and Collaborators, Recruitment Status, Location, Investigator, ClinicalTrials.gov Identifier** | **First Posted, Results First Posted, Last Update Posted,** | **Phase, Study Type, Allocation, Masking, Estimated Enrollment, Actual Enrollment** | **Intervention** | **Eligibility Criteria: Inclusion/ Exclusion** | **Primary and Secondary Outcomes** | **Reporting Groups: Description** | **Measures of Adherence: Planned & Posted** | **Reporting of Clinical Trials Outcome** | **Altered Outcomes** |
| **Developing a Mobile Health Pain-Coping Skills Training Program for the Treatment of Chronic Migraine: AIM 4** ^16^  Wake Forest University Health Sciences  Duke University  Hackensack Meridian Health  Not yet recruiting  North Carolina, USA  Donald B Penzien, PhD  [NCT03465826](https://clinicaltrials.gov/show/NCT03465826) | March 14, 2018  N/A  March 14, 2018 | N/A  Interventional (Clinical Trial) (clinical trial)  Randomized  Double (Investigator, Outcomes Assessor)  144 participants  N/A | PainCOACH is an 8-week internet-based pain coping skills training (PCST) program for chronic migraine. Has key therapeutic components of in clinic protocols + 8 modules of interactive training in cognitive behavioral pain coping skills. A virtual coach provides verbal and visual instruction, feedback and encouragement.  Experimental: PainCOACH Pain Coping Skills Training  4 weeks of daily headache monitoring, baseline questionnaires followed by 8 weeks of the PainCOACH migraine mHealth pain coping skills training program. After 8 weeks, participants complete post-treatment assessments and complete follow-up assessments at 3 and 6 months.  Active Comparator: TAU  Headache diaries for 4 weeks, followed by baseline questionnaires + 8 weeks daily headache monitoring. Post-assessment + follow up assessment at 3 and 6 months. | >18 y/o  Chronic migraine (>15 headaches/month)  Headache disorder other than migraine  Δ in preventative medications within 3 weeks prior to enrollment  Pain disorder other than migraine  Pregnant or planning pregnancy  Medical or psychiatric comorbidities that interfere with participation | Feasibility and Engagement (Through study completion, average of 9 months)-Single outcome measure measured using the PAINCOACH I questionnaire. Evaluates patients’ experiences and satisfaction with PainCOACH during post treatment assessment interval. Includes both qualitative and quantitative survey items 1-5.  Δ in Migraine Disability [2 months, 5 months, and 8 months]  Δ in HIT-6  Δ in pain intensity  Δ in headache management self-efficacy  Δ in headache locus of control scale  Δ in pain catastrophizing | N/A | N/A | N/A | N/A |
| **A Study of the Effect of a Disease-Specific Migraine Smart Phone Application (App) on Participant Care**  ^19^  Eli Lilly and Company  Atrium Health  Recruiting  Fort Mill, South Carolina, United States, 29715  Scott L Furney  NCT03559088 | June 15, 2018  N/A  September 21, 2018 | N/A  Interventional (Clinical Trial) (clinical trial)  Non-Randomized  None (open label)  200 participants  N/A | Goal is to determine if Smart phone application to foster communication between study participants and their doctor impacts care.  Experimental: Migraine Participants Using Application (App)  Participants w/ migraine history or recent prescription for common medication use migraine app linked to electronic health record (EHR) w/ results reported in EHR.  No intervention: Controls not using the app  Without migraine app linked to their EHR. | 18-70 years  Have a diagnosis of migraine since October 2015 or record of being prescribed a triptan prior to study  Have not been prescribed preventative migraine medication within past 12 months  Have not seen neurologist in the past year  Upcoming visit with Atrium Health PCP  Do not own smartphone  Currently participating in clinical trial involving investigational drug  Used the app before  Pregnant at time of study entry | # of Participants Receiving a Preventive Migraine Prescription (Next primary care appointment following use of app (estimated as 5 weeks))  # of months of app use (Baseline through one year)  # of Participants Receiving Acute and Preventive Migraine Treatments, Opioids, Barbiturates  # of Acute Care Visits  # of Primary Care Visits  # of Emergency Room Visits  # of Urgent Care Visits  # of Referrals to a Neurologist  # of New Diagnoses of Migraine or Chronic Migraine | N/A | N/A | N/A | N/A |
| **RELAXaHEAD for Headache Patients** ^17^  New York University School of Medicine,  National Multiple Sclerosis Society  Recruiting  New York, New York, USA  Mia Minen, MD, MPH  NCT03183791 | June 12, 2017  N/A  January 25, 2018 | N/A  Interventional (Clinical Trial) (clinical trial)  Randomized  Double (Investigator, Outcomes Assessor)  90 participants  N/A | Assess the utility of smartphone-based progressive muscle relaxation (PMR) for treatment of headaches. RELAXaHEAD app has electronic PMR and functionality to track medications.  Experimental: RELAX group  the RELAXaHEAD app  Rationale for PMR + review the app focusing on user knowledge, usability and engagement. 5-minute PMR session and discuss optimal time to practice PMR at home + enter headache log daily on the app.  Active Comparator: Monitored Usual Care (MUC) group  PMR component blocked on the app. Patients track headache frequency, intensity and acute medication use on the app. | 18-80 years  Meets criteria based on International Classification of Headache Disorders 3 beta  Migraine Disability Assessment (MIDAS) >5 or meets chronic PTH criteria based on International Classification of Headache Disorders 3 beta and has 4+ headaches days/month, 3-12 months post initial injury  Have had Cognitive Behavioral Therapy, Biofeedback or other Relaxation Therapy in the past year  Cognitive deficit or other physical problem that could interfere with behavioral therapy  Alcohol or substance abuse  Opioid or barbiturate use 10+ days/month  PHQ9 score of severe depression | Percentage of patients who did PMR ≥4/7 days of the week (6 months)  # of days spent doing PMR≥5 minutes/day as determined with backend analytics in the RELAXaHEAD app (6 months)  Minutes/day spent doing PMR (6 months)  Satisfaction using Likert scale questions on RELAXaHEAD usability, content and functionality (6 months)  MIDAS between scores at 3 months and baseline (3 months) | N/A | N/A | N/A | Changed December 29, 2017: Proportion of patients who enrolled in the study/were recruited for the study was eliminated as a primary outcome measure. Satisfaction using Likert scale questions on RELAXaHEAD usability, content, functionality was added as a primary outcome measure. |
| **Improving Health Outcomes of Migraine Patients Who Present to the Emergency Department** ^18^  New York University School of Medicine  Not yet recruiting  New York, New York, USA  Mia Minen, MD, MPH  [NCT02945839](https://clinicaltrials.gov/show/NCT02945839) | October 26, 2016  N/A  May 31, 2018 | N/A  Interventional (Clinical Trial) (clinical trial)  Randomized  Double (Participant, Investigator)  90 participants  N/A | Aspects of this study include, initiation of preventive medication in timely manner for individuals who present to the ED, introduction of PM + PMR in the ED, introduction of smartphone application based behavioral therapy in the ED to reduce headache disability, frequency + intensity.  Active Comparator: Acute Treatment +ED + initiated preventive medication + PMR  Discharged on acute migraine therapy along with progressive muscle relaxation therapy.  Active Comparator: Enhanced Usual Care (EUC)  Migraine treatment decisions based on ED attending. RELAXaHEAD without PMR to track headache frequency, intensity and acute medication on the app. | 18-64 years  Meet migraine criteria or 4+ headaches/month  MIDAS score > 5  Have had Cognitive Behavioral Therapy, Biofeedback or other Relaxation Therapy in the past year  Cognitive deficit or other physical problem that could interfere with behavioral therapy  Alcohol or substance abuse  Opioid or barbiturate use 10+ days/month  PHQ9 score of severe depression | MIDAS at 3 months and baseline (12 Weeks) Decrease of 3 points in the MIDAS score corresponds to a 1-day reduction in headache related disability/month.  Perceived Stress Scale (PSS) (12 weeks)  # of days/week treated with acute medications # of drug administrations/week for acute medications | N/A | N/A | N/A | N/A |

| **MAJOR DEPRESSIVE DISORDER** | | | | | | | | | | |
| --- | --- | --- | --- | --- | --- | --- | --- | --- | --- | --- |
| **Title, Sponsors and Collaborators, Recruitment Status, Location, Investigator, ClinicalTrials.gov Identifier** | **First Posted, Results First Posted, Last Update Posted,** | **Phase, Study Type, Allocation, Masking, Estimated Enrollment, Actual Enrollment** | **Intervention** | **Eligibility Criteria: Inclusion/ Exclusion** | **Primary and Secondary Outcomes** | **Reporting Groups: Description** | **Outcome Measures** | **Measures of Adherence: Planned and Posted** | **Reporting of Clinical Trials Outcome** | **Altered Outcomes** |
| **Developing Accessible mHealth Programs for Depression Management in Bolivia**  ^96^  University of Michigan  Completed  La Paz, Bolivia  John D. Piette, PhD,  [NCT02765542](https://clinicaltrials.gov/show/NCT02765542) | May 6, 2016  N/A  May 6, 2016 | N/A  Interventional (clinical trial)  N/A  None (Open Label)  N/A  32 participants | Evaluate the feasibility and potential impact of an automated phone system in monitoring and improving self-care and health outcomes among patients with depression in Bolivia. Weekly 10-15 automated phone call for disease assessment and self-care support for up to 12 weeks. On phone call, asked questions about self-care relevant to diagnosis, symptoms of depression and medication adherence. Based on self-report, receive targeted suggestions for how to improve self-management. If patient reports health or self-care program during call, follow up call will take place with designated clinician.  Experimental: Automated phone calls  Automated disease assessment and self-care support phone calls for up to 12 weeks.  Other: Automated disease assessment and self-care support phone calls  Automated disease assessment and self-care support phone calls for up to 12 weeks. | 21-80 years  PHQ-8 of 10 or higher  Diagnosis indicating 6-month life expectancy  Prior inpatient psychiatric treatment  Patients w/ probable bipolar disorder or cognitive impairment | **Δ** from baseline on depressive symptoms at 12 weeks (PHQ-8 scores) (Time Frame: Baseline and 12-week post intervention follow up)  **Δ** from baseline on self-care behaviors at 12 weeks (self-care behavior questionnaire) (Time Frame: Baseline and 12-week post intervention follow up)  Evaluate program feasibility (telephone call completion rates) (Time Frame: 12-week post intervention follow up)  Patient satisfaction questionnaire  Patient qualitative feedback questionnaire | N/A | N/A | N/A | N/A | N/A |
| **AniMovil mHealth Support for Depression Management in Low-Income Country (AniMovil)**  ^24^  University of Michigan  Universidad de Los Andes, Bogota, Colombia  Recruiting  Bogota, Colombia  John Piette, PhD  [NCT03615118](https://clinicaltrials.gov/show/NCT03615118) | August 3, 2018  N/A  August 27, 2018 | N/A  Interventional (clinical trial)  Randomized  None (Open Label)  114 participants  N/A | Interactive voice response and test messaging to support effective depression care. Weekly automated (IVR) Calls and daily test messages throughout 12-week intervention. Patients w/ more severe depression will receive up to 12 weekly community health workers (CHWs) delivered telephone CBT sessions. CHWs use patients IVR contacts to enhance psychoeducation.  Experimental: Intervention  Anemoi intervention-Sentirse Mejor manual for information about CBT and skill practice, weekly IVR depression symptom assessments + psychoeducational messages, daily SMS mood monitoring and CBT reinforcement messages and CHW telephone CBT sessions if elevated PHQ-9 scores during the study. Daily SMS messages to rank mood on scale from 1 to 9. Automated phone system for monitoring and improving self-care and health outcomes.  Active Comparator: Enhanced Usual Care  Receive usual care, including the Sentirse Mejor + daily SMS messages asking participants to report their mood on a scale from 1 to 9. Patients who report mood scores of 1 3 2 for at least 3 days per week and 3 consecutive weeks will be called by community health worker and referred to national program office for depression support services. | 21-60 years  Score of 10+ on the Spanish-validated version of the PHQ-9  Less than 6-month life expectancy  History of psychiatric hospitalization or bipolar disorder  Substance use disorder or cognitive impairment | Severity of depression symptoms as measured by the PHQ-9 (Time Frame:  **Δ** in PHQ scores at Baseline and 3 month follow up)  Health related quality of life as measured by the Short Form Survey (SF-12) (Time Frame: **Δ** in score at baseline and 3 month follow up)  Sheehan Disability Score (SDS)  Daily mood ratings (Time Frame: **Δ** in daily mood scores between days 1-90) | N/A | N/A | N/A | N/A | N/A |
| **mHealth for Antenatal Mental Health**  ^97^  Imperial College London  Completed  United Kingdom  Jose S Marcano Belisario  Josip Car  Cecily Morrison  John O’Donoghue  [NCT02516982](https://clinicaltrials.gov/show/NCT02516982) | August 6, 2015  N/A  September 6, 2018 | N/A  Observational  N/A  N/A  N/A  880 participants | Determine feasibility of using mobile technology for implementing the recommendations of the antenatal and postnatal mental health: clinical management and service guidance NICE guideline for recognizing depression during pregnancy using iPad tables in the waiting areas of general practices and using a bespoke app running on pregnant women’s own smartphones to monitor mood and symptoms of depression throughout pregnancy.  Screening-scrolling layout  Survey on demographic information, Whooley questions, Edinburgh postnatal depression. Presented on a single screen; participant will have to scroll vertically to answer all questions. Whooley questions are case finding instrument for depression in primary care. 2 question instrument screens for depressed mood and anhedonia that have present in the past month. The Edinburgh Postnatal Depression Scale is a 10 item self-administered survey that screens for symptoms such as feelings of guilt, sleep disturbance, reduced energy levels, anhedonia and suicidal ideation in the past 7 days. Each question is scored on a 4 point scale.  Screening-Paging layout  Demographic information survey, Whooley questions, Edinburgh Postnatal Depression Scale. Only 1 question presented at any given time. Navigate multiple pages to answer all questions.  Retrospective plus momentary assessment  Download app and complete sampling protocol for 6 consecutive days, once a month for 6 months. During 6 assessments day, participant will complete the Edinburgh Postnatal Depression scale, 5 momentary questions assessing mood, sleep, energy, enjoyment and worry and 2 contextual questions asking for location and activity at time they completed the questions.  Retrospective assessment  Download app and complete sampling protocol consisting of 1 day a month for 6 months. Assessment days will consist of single administration of the Edinburgh Postnatal Depression Scale. | 18+  Pregnant women attending antenatal clinics  Diagnosis of any common mental health disorder  Receiving treatment for any common mental health disorder | Positive predictive value of the Whooley questions [ Time Frame: One-off outcome assessment conducted up to 24 hours after obtaining written, informed consent ]-Calculate number of pregnant women who answered Yes to any of the Whooley questions AND who scored 10 points or higher on the Edinburgh Postnatal Depression Scale (EPDS), as a proportion of the total number of pregnant women who answered Yes to any of the Whooley questions regardless of their EPDS scores. Negative predictive value of the Whooley questions [ Time Frame: One-off outcome assessment conducted up to 24 hours after obtaining written, informed consent  False Omission Rate of the Whooley questions [ Time Frame: One-off outcome assessment conducted up to 24 hours after obtaining written, informed consent ] Adherence to sampling protocol [ Time Frame: Assessed after 6 months ]- Proportion of expected assessments completed as a proportion of the total number of expected assessments.  Mean overall scores on the Edinburgh Postnatal Depression Scale [ Time Frame: One-off outcome assessment conducted up to 24 hours after obtaining written, informed consent ] Breakoff rates-Proportion of participants who interrupt the survey completion process before reaching the end of the survey questionnaires. Document reason for breakoff. Time needed to complete survey questionnaires Proportion of complete records  . | N/A | N/A | N/A | N/A | Submitted September 9, 2016: Adherence to sampling protocol (Time Frame: Assessed after 6 months) was added as a primary outcome measure. |
| **Scaling Up Science-based Mental Health Interventions in Latin America** ^98^  Dartmouth-Hitchcock Medical Center  Pontificia Universidad Javeriana  National Institute of Mental Health (NIMH)  Recruiting  Lisa A Marsch, PhD, Dartmouth College  Carlos Gomez-Restrepo, MD, Pontifica Universidad Javeriana  [NCT03392883](https://clinicaltrials.gov/show/NCT03392883) | January 8, 2018  N/A  March 26, 2018 | N/A  Interventional (Clinical Trial)  None (Open Label)  N/A  2000 participants  N/A | Utilize mobile behavioral health technology for mental health w/ primary focus on depression and secondary focus on problematic alcohol and other substance abuse, launch new workforce training and service delivery models, launch and evolve an integrated data management system for systematic data tracking and outcomes assessment, launch and grow a learning collaborative of organizations integrating mental health into primary care.  Experimental: Digital Health Assisted Mental Healthcare  Laddr, a mobile behavioral health technology. Offers science based self-regulation monitoring and health behavior change tools. Structured to focus on a user’s management of depression and impact on functioning and quality of life. Secondarily focus on problematic alcohol use and relationship to depression management. | 18+  Patient at one of collaborating PC sites  Screen positive for depression on PHQ-9 and/or screen positive for problematic alcohol use of Alcohol Use Disorder Identification Test  Diagnosis w/ co-occurring severe mental illness  Alcohol withdrawal symptoms that require higher level of care  Express suicidal intention | The Integrated Measure of Implementation Context and Outcomes as a **Δ** Over Time in Low and Middle Income Countries Consumer Instrument [ Time Frame: 6 months before implementation launch at a given site (starting with baseline in late 2017); at the time of implementation launch; and every 6 months thereafter at each site until study completion (July 2021). ]  The Integrated Measure of Implementation Context and Outcomes as a **Δ** Over Time in Low and Middle Income Countries Provider Instrument  The Integrated Measure of Implementation Context and Outcomes as a **Δ** Over Time in Low and Middle Income Countries Organizational Staff Instrument  The Integrated Measure of Implementation Context and Outcomes as a **Δ** Over Time in Low and Middle Income Countries Sustainability Instrument for Providers  The Integrated Measure of Implementation Context and Outcomes as a **Δ** Over Time in Low and Middle Income Countries Sustainability Instrument for Organizations  Patient Health Questionnaire (PHQ-8) [ Time Frame: These patient measures will be assessed at baseline and every 3 months for a period of 12 months (for a total of 5 assessment timepoints per patient). ]  Alcohol Timeline Follow-back (Alcohol TLFB)  12-item World Health Organization Disability Assessment Schedule 2.0 (WHODAS)  EuroQOL Five Dimensions Questionnaire (EQ-5D)  General Anxiety Disorder screener (GAD-7)  EuroQol Visual Analoge Scale (EQ-VAS) | N/A | N/A | N/A | N/A | N/A |
| **Text-Message-Based Depression for High-Risk Youth in the ED**  ^25^  Rhode Island Hospital  National Institute of Mental Health (NIMH)  Completed  Providence, RI, USA 02903  Megan Ranney, MD, MPH  [NCT02332239](https://clinicaltrials.gov/show/NCT02332239) | January 6, 2015  December 7, 2017  December 7, 2017 | N/A  Interventional (Clinical Trial)  Randomized  Single (Outcomes Assessor)  N/A  116 participants | iDOVE, a brief emergency department introductory session and longitudinal automated text message depression prevention program for high risk teens. CBT and motivational interviewing (MI) depression and violence prevention interventions to introduce basic cognitive and behavioral strategies. 8 weeks of tailored CBT-informed daily text messages sent to enhance skills and remind participants of self- determined goals.  Experimental: iDOVE Intervention (ED+text)  In-ED brief session, introducing basic principles of CBT and structure of text messaging intervention. 8 week longitudinal tailored CBT-based text message program.  Placebo Comparator: Control (EUC)  In-ED brief session, discussing home safety and nutrition. 8 week longitudinal home safety and nutrition text message program. | 13-17 years  Presenting to ED for routine care  Reporting past year physical violence and current mild to moderate depressive symptoms  Accompanied by parent who can give consent  Chief complaint of suicidal ideation, psychosis or child abuse  In police custody  Severe depressive symptoms | **Δ** in Depressive Symptoms (Time Frame: Enrollment, 8 weeks post enrollment, 16 weeks post enrollment)-Beck’s Depression Inventory (BDI-2)  **Δ** in Peer Violence Involvement-Physical Assault subscale of the Conflict Tactics Scale (CTS-2)  Acceptability/Feasibility: Follow Up Rate (Time Frame: Enrollment, 8 weeks post enrollment, 16 weeks post enrollment) Acceptability/Feasibility: Engagement of Intervention Group (Enrollment to 16 weeks post enrollment) Acceptability/Feasibility: Patient Satisfaction (Time Frame: 8 weeks post enrollment)-CSQ-8 | Refer to intervention | N/A |  | Results posted on clinical trials | Submitted: January 5, 2015- **Δ**  in Depressive Symptoms was the primary outcome measure. Submitted November 7, 2017- **Δ**  in Peer Violence Involvement was added as a primary outcome measures.  Submitted January 5, 2015-original secondary outcome measures was  **Δ**  in Peer Violence Involvement.  Submitted January 5, 2015: Acceptability/Feasibility: Follow Up Rate, Acceptability/Feasibility: Engagement of Intervention Group and Acceptability/Feasibility: Participant Questionnaire were the secondary outcome measures. |
| **An Adaptive Intervention for Depression Among Latinos Living With HIV**  ^26^  University of California, San Francisco  Not yet recruiting  San Francisco, California, USA  John Sauceda, Assistant Professor  [NCT03668379](https://clinicaltrials.gov/show/NCT03668379) | September 12, 2018  N/A  September 12, 2018 | N/A  Interventional (clinical trial)  Randomized  Double (Care Provider, Outcomes Assessor)  45 participants  N/A | Adaptive treatment strategy (ATS) that has a mobile health (mHealth) tool (text messaging) to improve adherence to treatment for depression. Treatments tested are behavioral activation therapy (BAT) and CBT.  Active Comparator: BAT  BAT is a behavioral theory in treatment for depression. A total of 5 1-hour sessions delivered every 2 weeks. During session 1, focus on introduction to BAT. Sessions 2 and 3 review initial session. Session 4 and 5 will review progress, challenges and maintenance strategies.  Behavioral: Augment w/ mHealth-non responders to first stage treatment of BAT alone may be re randomized to receive text message support + BAT intervention.  Behavioral: Maintenance-BAT or BAT +mHealth continue in maintenance mode. Not receive any additional interventions during 2^nd^ stage treatment.  Behavioral: Switch to CBT and mHealth-non-responders to 1^st^ stage treatment of either BAT or BAT and mHealth may be randomized to switch interventions to intensified CBT. CBT 8 sessions long.  Experimental: Behavioral Activation Therapy and mHealth  Initial stage treatment, experimental arm will deliver BAT program identical to active comparator arm +mHealth component in the form of one way and two way SMS text messages. Direct personalized text messages delivered 2x/week. 1 way messages sent as appointment and BAT adherence reminders. 2 way messages 1x/week create mobile drop in clinic where messages can be sent and received. | 18+  HIV positive  Self identifies as Latino/Hispanic  Fluent in English or Spanish  Receives HIV care at study site clinic  PHQ-9 >9  Agrees to discuss depression, treatment preferences and mHealth  Exclusion criteria negation of inclusion criteria | Composite measure of feasibility for the adaptive treatment strategy (ATS) (Time Frame: 4 months)-one composite measure at the end of the second stage treatment. Total number of participants screened, eligible and enrolled; at least 80% of all BAT and CBT sessions completed; 80% retention of participants across all outcome groups Composite measure of acceptability for the adaptive treatment strategy-greater than 90% adherence to the BAT and CBT session schedule, responsiveness to text messages a) >80% of all 2 way text messages replied back during set of blocked hours b) >90% of participants reporting direct benefit from 1 way text messages; Acceptability responses coded from post-intervention exit interview transcripts, limited number of barriers to participation reported participants in exit interview, responses to a brief survey assessing clinic staff acceptability of the intervention  Self-reported Adherence to Antiretroviral Therapy (ART) (Time Frame: 12 months)-visual analog scale, a 10 cm line on which participants indicate % of doses of all HIV medications taken in the past 30 days Viral Load-amount of HIV virus in the blood PHQ-9 Engagement in HIV care-10 item unit dimensional patient centered scale that assesses provider, clinic and patient level characteristics associating with being engaged in HIV care | N/A | N/A | N/A | N/A | N/A |
| **Intervention to Prevent Peer Violence and Depressive Symptoms Among At-Risk Adolescents (iDOVE2)** ^27^  Rhode Island Hospital  Eunice Kennedy Shriver National Institute of Child Health and Human Development (NICHD)  Recruiting  Providence, RI, USA, 02903  Megan L Ranney, MD, MPH  [NCT03626103](https://clinicaltrials.gov/show/NCT03626103) | August 10, 2018  N/A  August 10, 2018 | N/A  Interventional (Clinical Trial)  Randomized  Single (Outcomes Assessor)  800 participants  N/A | Test the efficacy of iDOVE2, a brief emergency department introductory session and longitudinal automated text message depression prevention program for high risk teens and to determine the best combination of intervention for preventing peer violence and depressive symptoms among at risk youth.  Experimental: + Brief ED Intervention (BI), _+ Text  Brief ED Intervention component (20 minute MI and CBT based in ED session) and the text messaging intervention component (8 weeks of an automated, 2 way text message curriculum starting after ED visit which reinforces cognitive reappraisal, emotional regulation and self-efficacy skills).  Experimental: + Brief ED Intervention (BI), no Text  Brief ED intervention component only  Experimental: No Brief ED Intervention (BI), + Text  Text message intervention component only + brochure containing only and community resources for violence and depression prevention instead of the Brief ED Intervention component  No Intervention: No Brief ED Intervention (BI), no Text  Only receive a brochure containing online a community resources for violence and depression prevention, instead of the Brief ED Intervention component. | 13-17 years  Presenting to ED for routine care  Reporting past year physical violence as identified on a brief screen administered in the ED  Reporting past 2 week mild to moderate symptoms PHQ-9 score 5-19  Accompanied by parent who can give consent  Chief complaint of suicidal ideation, psychosis or child abuse  In police custody  In need of emergency psychiatric care | Conflict Tactics Scale-2, physical subset (CTS-2) (Time Frame: Enrollment, 2 months post enrollment, 4 months post enrollment, 8 months post enrollment)- **Δ** from enrollment physical peer violence range 0-56 Centers for Epidemiologic Studies Depression Scale Revised (CESD-R)- **Δ** from enrollment depressive symptoms. Score summed based on symptom group, range 0-80.  **Δ** in ED Visits for Assault-Related Injury (Time Frame: 12 months before enrollment and 12 months after enrollment) Conflict in Adolescent Dating Relationships Inventory, physical subject CADRI (Time Frame: Enrollment, 2 months post enrollment, 4 months post enrollment, 8 months post enrollment)-changes from enrollment other forms of peer violence (dating relationships) Illinois Bully Scale (IBS) and Student School Survey) | N/A | N/A | N/A | N/A | N/A |
| **Cognitive Behavioral Therapy Treatment of Depression With Smartphone Support**  ^28^  Linkoeping University  Completed  Linköping, Östergötland, Sweden  Gerhard Andersson, Professor  [NCT01819025](https://clinicaltrials.gov/show/NCT01819025) | March 27, 2013  N/A  February 7, 2014 | N/A  Interventional (clinical trial)  Randomized  None (Open Label)  N/A  88 participants | Investigate whether face-to-face CBT with a smartphone application, focused on providing support in homework and an increase in behavioral activation is effective in treating mild to moderate depression.  Experimental: 4 face-to-face and smartphone-app  4 face to face therapy sessions and smartphone app as a complement and support to the 4 sessions.  Active Comparator: TAU  10 sessions of face to face therapy, full behavioral activation | 18+  Depressive symptoms according to DSM-IV  Good knowledge of the Swedish language  Recent during last 6 weeks change in psychiatric medication  Presently in any other psychological treatment  Severe depression  Suicidal ideation | PHQ-9- **Δ** from baseline [Time Frame: Two weeks pre-treatment, two weeks post treatment, six months and 12 months post treatment]  Beck Depression Inventory (BDI)- **Δ** from baseline  **Δ** of Life Inventory (QOLI)-change from baseline [Time Frame: Two weeks pretreatment, two weeks post treatment, six months and 12 months post treatment]  Acceptance and Action Questionnaire (AAQ)- **Δ** from baseline  Beck Anxiety Inventory (BAI)- **Δ** from baseline  Trimbos and iMTA questionnaire on Costs associated with psychiatric illness (TIC-P)- **Δ** from baseline (Time Frame: Two weeks pre-treatment and at 6 months post treatment) | N/A | N/A | N/A | +1 Paper Submitted | N/A |
| **Behavioural Activation-Based Treatment Administered Through Smartphone**  ^99^  Linkoeping University  Completed  Linköping, Östergötland, Sweden  Gerhard Andersson, Professor  [NCT01463020](https://clinicaltrials.gov/show/NCT01463020) | November 1, 2011  N/A  June 19, 2013 | N/A  Interventional (Clinical Trial)  Randomized  None (Open Label)  N/A  81 participants | Test the effects of a smartphone delivered behavioral activation treatment. Moderator analysis of low and high severity of depressive symptoms will be made.  Experimental: Smartphone delivered BA  8 week behavioral activation therapy delivered through smartphone  Active Comparator: Smartphone delivered mindfulness  8 week mindfulness intervention delivered through smartphone | 18-65 years  Depressive symptoms according to DSM-IV  Good knowledge of the Swedish language  Recent during last 6 weeks **Δ** in psychiatric medication  Presently in any other psychological treatment  Severe depression  Suicidal ideation | PHQ-9- **Δ** from baseline [Time Frame: Two weeks pre-treatment, two weeks post treatment, six months and 12 months post treatment]  Beck Depression Inventory (BDI)- **Δ** from baseline  **Δ** of Life Inventory (QOLI)- **Δ** from baseline [Time Frame: Two weeks pre-treatment, two weeks post treatment, six months and 12 months post treatment]  Acceptance and Action Questionnaire (AAQ)- **Δ** from baseline  Beck Anxiety Inventory (BAI)- **Δ** from baseline  Trimbos and iMTA questionnaire on Costs associated with psychiatric illness (TIC-P)- **Δ** from baseline (Time Frame: Two weeks pre-treatment and at 6 months post treatment) | N/A | N/A | N/A | +1 Paper Submitted  *1 Paper Submitted | Original Primary Outcome Measures (submitted: October 31, 2011) was the Montgomery Asberg Depression Rating Scale-Self Rated (MADRS). Current Primary Outcome Measures (submitted: March 22, 2013) is the PHQ-9 and Beck Depression Inventory (BDI).  Original Secondary Outcome Measures (submitted: March 22, 2013) was the QOLI, AAQ, BAI and TIC-P. Current Secondary Outcome Measures (submitted March 22, 2013) eliminated the AAQ and BDI as secondary outcome measures. |
| **Cognitive-behavioral Intervention via a Smartphone App for Depressive Symptoms in Caregivers (App Depression)**  ^29^  Ministerio de Economía y Competitividad, Spain  Galicia: Ministry of Work and Welfare (Xunta de Galicia)  Active, not recruiting  Santiago de Compostela, A Coruna, Spain 15782  Fernando L. Vázquez González, Ph.D.  [NCT03110991](https://clinicaltrials.gov/show/NCT03110991) | April 12, 2017  N/A  October 25, 2017 | N/A  Interventional (Clinical Trials)  Randomized  Triple (Participant, Care Provider, Investigator)  174 participants  N/A | Evaluate the effectiveness of a cognitive-behavioral intervention in the prevention of depression, administered through a smartphone application, both without and without telephone contact through multi conferencing.  Experimental: Cognitive-behavioral intervention via App  Participants of 2 experimental groups will receive cognitive behavioral intervention for depression prevention via smartphone .  Experimental: Cognitive-behavioral intervention via App + multiconference  This experimental group will receive phone group conference calls during four 30 minute sessions.  No Intervention: Usual care  No intervention or material but will have unrestricted access to any routine medical or psychological care they might want to seek depressive symptoms. Use of such treatments will be recorded. | 18+  Serves as an informal caregiver for dependent family member  Dependence recognized the Xunta de Galicia  CES-D ≥ 16  Not suffering from a depressive episode  Having received psychological or pharmacological treatment in previous 2 months Present other conditions may act as cofounders  Presenting serious psychological or medical disorders that require immediate intervention or prevent study implementation  Serious or terminal prognosis next 14 months | **Δ** from baseline Major depressive episode to post-treatment (6 weeks), and follow-ups at 1, 3, 6 and 12 months [Time Frame: Pre- and post-intervention (6 weeks) with follow-ups at 1, 3, 6, and 12 months]-assessed with the Structured Clinical Interview for the DSM-5  **Δ** from baseline depressive symptomatology to post-treatment (6 weeks), and follow-ups at 1, 3, 6 and 12 months [ Time Frame: Pre- and post-intervention (6 weeks) with follow-ups at 1, 3, 6, and 12 months ]-Center for Epidemiological Studies Depression Scale (CES-D), a 20 item scale self-administered and assesses depressive symptoms | N/A | N/A | N/A | * 1 Paper Submitted*1 Abstract Submitted | N/A |
| **Reducing Depressive Symptomatology With a Smartphone App** ^30^  Babes-Bolyai University  Norwegian University of Science and Technology  CheckWare AS  Active not recruiting  Cluj-Napoca, Cluj, Romania, 400084  Cezar Giosan, PhD,  [NCT03060200](https://clinicaltrials.gov/show/NCT03060200) | February 23, 2017  N/A  July 12, 2018 | N/A  Interventional (Clinical Trials)  Randomized  Single (Investigator)  220 participants  N/A | Test newly developed app firmly grounded in CBT theory of depression to determine is app is clinically useful in decreasing moderate depressive symptoms when compared with an active placebo. Also interested in app’s potential to contribute to the reduction of general negative affect, increasing positive affect and boosting satisfaction with life or can modify depressogenic cognitions.  Experimental: Active Intervention  Self-administered online CBT plus therapist check in. Moderately depressed participants will be testing depression app, self-administered plus therapist check in, online CBT intervention for 6 weeks. App is comprised of courses on psychoeducational background of the program. Exercises use the information presented in the courses and follow structure of regular therapy session/ therapeutic homework. Behavioral activation consists of goals and activities.  No Intervention: Waiting list  Wait list for 6 weeks after which access to the depression app will be given.  Placebo Comparator: Placebo  Sham self-administered online CBT plus therapist check in. Intervention will include the same sections and features as the original app, except for the complete exercises and behavioral activations sections. Psychoeducation section will include different content, elaborating on common sense information on psychological well-being. | 18-60 years  Romanian speaking  PHQ-9 score between 10 and 16  Undergoing treatment medication and/or psychotherapy  Substance abuse problems  Psychotic problems  Organic brain disorders  Self-injury or harming others  Suicidal ideation | Center for Epidemiologic Studies Depression Scale-Revised (CESD-R) (**Δ** from baseline) [ Time Frame: Baseline, 1 week after baseline, 2 weeks after baseline, 3 weeks after baseline, 4 weeks after baseline, 5 weeks after baseline, post-intervention (6 weeks after baseline), Follow-up (3 months post-intervention) ]  The Positive and Negative Affect Scale (PANAS) (**Δ** from baseline) [ Time Frame: Baseline, mid-intervention (3 weeks after baseline), post-intervention (6 weeks after baseline), Follow-up (3 months after post-intervention) ]  The Behavioural Activation for Depression Scale - Short Form (BADS-SF)( **Δ** from baseline)  Satisfaction with Life (SWL) (**Δ** from baseline) | N/A | N/A | N/A | *1 Paper Submitted | N/A |
| **Mobile Sensing and Support for Depression**  ^100^  University of Zurich  Switzerland: ETH  Switzerland: Makora  Completed  Switzerland  S Weidt, MD,  [NCT02776839](https://clinicaltrials.gov/show/NCT02776839) | May 18, 2016  N/A  June 2, 2017 | N/A  Interventional (Clinical Trial)  N/A  None (Open Label)  N/A  126 participants | Explore the potential of context sensitive intervention to provide in site support for people with depressive symptoms and explore the detection of daily life behavior based on smartphone sensor information to identify subjects with clinically meaningful depression level.  Mobile Sensing: Designed to develop the app. Algorithms the app should learn to detect depression symptoms. | 18+  Self-declaration of depressive symptoms  German speaking  Psychotic symptoms  Bipolar symptoms  Drug or alcohol dependency  Dementia | PHQ-9 for depression (Time Frame: Baseline)-subjective depressive symptoms to compare detected symptoms measured by the app.  Client Satisfaction Questionnaire (ZUF-8)-measures client satisfaction with the app (Time Frame: at post at least 6 weeks after baseline, maximum 6 months) | Results submitted-Not posted on ClinicalTrials.gov | Results submitted-Not posted on ClinicalTrials.gov | N/A | Results submitted-Not posted on ClinicalTrials.gov | N/A |
| **Mobile Technology to Engage and Link Patients and Providers in Antidepressant Treatment (MedLink)** ^101^  Northwestern University  Completed  Chicago, Illinois, USA  David C Mohr, PhD,  [NCT02583230](https://clinicaltrials.gov/show/NCT02583230) | October 22, 2015  October 29, 2018  October 29, 2018 | N/A  Interventional (clinical trial)  N/A  None (Open Label)  N/A  11 participants | Develop and pilot a mobile smartphone delivered intervention that will improve antidepressant medication care by providing medication adherence monitoring and support to the patient, feedback on patient adherence and response to treatment to primary care team and information to both patients and providers on guideline congruent care personalized to the patient’s response to antidepressant medication.  Experimental: MedLink  8 weeks, patient who is newly prescribed antidepressant medication will receive a mobile phone app and a GSM enable pill bottle in order to provide and receive feedback regarding medication adherence. Patient adherence to antidepressant medication will be accomplished by 1) monitoring adherence and providing feedback to patient 2) monitoring side effects and treatment response and providing in the moment feedback and support 3) activating patient to take appropriate action based upon monitoring data 4) providing standardized education and positive reinforcement to the patient | 18+  Has been prescribed an antidepressant medication by PCP but has not yet initiated treatment  Has depression determined by PCP  Currently taking an antidepressant medication or has taken 1 in the previous 3 months  Diagnosed w/ psychotic disorder, bipolar disorder, dissociative disorder, current substance dependence or other diagnosis for which participation is either inappropriate or dangerous  Is severely suicidal (ideation, plan and intent) | 1.Adherence to Antidepressant Medication (Time Frame: 8 weeks)-number of days medication was taken when a dose was expected. Measured through % of days adherent on Wisepill pillbox as well self-reported adherence  2.PHQ-9 (Time Frame: Baseline, Week 4, Week 8) 3.Quick Inventory of Depressive Symptomatology Clinician Rating (QIDS-C) | Refer to intervention | 1.11 participants analyzed. Adherence to antidepressant medication was 82% of days adherent. 2. 11 participants analyzed Baseline=12.0 (4.0) Week 4=5.3 (4.6) Week 8=3.5 (6.5)  3. 11 participants analyzed baseline=111.6 (3.7) Week 4=7.6 (5.6)  Week 8=4.6 (4.0) | N/A | N/A | Original Primary Outcome Measures (submitted: October 20, 2015) was Adherence to Antidepressant Medication measured as the number of days medication was taken when a dose was expected. Current Primary Outcome Measures (submitted: February 14, 2018) is Adherence to Antidepressant Medication measured through % of days adherent on Wisepill pillbox as well as self-reported adherence.  Original Secondary Outcome Measures (submitted: October 20, 2015) was changes in depression measured through self-report PHQ-9 and usability measured through Likert scale ratings. Current secondary outcome measures (submitted: February 14, 2018) is the PHQ-9 and Quick Inventory of Depressive Symptomology Clinician Rating (QIDS-C). |
| [**Mobile Technology to Engage and Link Patients and Providers in Antidepressant Treatment**](https://clinicaltrials.gov/ct2/show/NCT01909973?cond=smartphone+depression&rank=19)^102^  Northwestern University  Completed  Chicago, Illinois, USA 60611  David C Mohr, PhD,  [NCT01909973](https://clinicaltrials.gov/show/NCT01909973) | July 29, 2013  N/A  August 4, 2017 | N/A  Interventional (clinical trial)  Randomized  Single (Outcomes Assessor)  N/A  15 participants | Develop and pilot a mobile smartphone delivered intervention that will improve antidepressant medication care by providing medication adherence monitoring and support to the patient, feedback on patient adherence and response to treatment to primary care team and information to both patients and providers on guideline congruent care personalized to the patient’s response to antidepressant medication.  Experimental: MedLink System  For 12 weeks, the patient who is newly prescribed antidepressant medication will receive mobile phone and GSM enable pill bottle in order to provide and receive feedback regarding medication adherence.  No Intervention: TAU  Continue to receive treatment as usual from their primary care doctor. Free mobile phone for the 12 weeks of the intervention | 18+  Has been prescribed an antidepressant medication by PCP but has not yet initiated treatment  Has depression determined by PCP  Currently taking an antidepressant medication or has taken 1 in the previous 3 months  Diagnosed w/ psychotic disorder, bipolar disorder, dissociative disorder, current substance dependence or other diagnosis for which participation is either inappropriate or dangerous  Is severely suicidal (ideation, plan and intent) | Adherence to Antidepressant Medication (Time Frame: Baseline to up to 12 weeks)-Frequency of medication from baseline to end of treatment  **Δ** s in depression (Time Frame: Baseline to up to 12 weeks)-severity of depressive symptoms from baseline to end of treatment. | Results Submitted - Not Posted on ClinicalTrials.gov | Results Submitted - Not Posted on ClinicalTrials.gov | N/A | Results Submitted - Not Posted on ClinicalTrials.gov | Original Primary Outcome Measures (submitted: July 24, 2013) was Adherence to Medication as measured by when the provided pill bottle is opened to remove a dose of medication and  **Δ**  in Depression Over Time through the PHQ-9. Current Primary Outcome Measures (submitted: January 12, 2015) is measured as Adherence to Antidepressant Medication measured as the frequency of medication usage from baseline to end of treatment.  Original Secondary Outcome Measures (submitted: July 24, 2013) was Presence of Side Effects and  **Δ** s Over Time measured by the Patient Rated Inventory of Side Effects (PRISE) and Frequency, Intensity and Burden of Side Effects Rating (FIBSER). Current Secondary Outcome Measures (submitted: January, 12 2015) is changes in depression measured as the severity of depressive symptoms from baseline to end of treatment. |
| **Lifestyle Intervention for Young Adults with Serious Mental Illness**  ^41^  Dartmouth-Hitchcock Medical Center  National Institute of Mental Health (NIMH)  Recruiting  Massachusetts, USA 02141  New Hampshire, USA 03101  Kelly Aschbrenner, PhD, Scientist  [NCT02815813](https://clinicaltrials.gov/show/NCT02815813) | June 28, 2016  N/A  March 13, 2018 | N/A  Interventional (Clinical Trial)  Randomized  Parallel Assignment  144 participants  N/A | 4 year project evaluating effectiveness of group based lifestyle intervention (PeerFIT) supported by mobile health technology and social compared vs. Basic Education in fitness and nutrition supported by a wearable Activity Tracking device (BEAT) in achieving clinically significant improvements in weight loss and cardiorespiratory fitness in young adults with serious mental illness.  Experimental: PeerFIT  12 month PeerFIT intervention consists of 6 month intensive phase including once weekly 60 minute group weight management + exercise session led by a lifestyle coach, once weekly 1 hour physical activity session delivered in community settings and Facebook and mHealth technology to increase motivation + self-monitoring and peer social support for health behavior **Δ**. Participants then transition to 6 month lower intensity phase in which the 90 minute weight management sessions are discontinued. Open groups with a minimum of 4 participants and maximum of 18 participants at any given time.  Behavioral :BEAT  Monthly individual lifestyle sessions delivered by a lifestyle coach including education, guidance and support for self-monitoring behaviors during first 6 months of the study + text message reminders and encouragement for self-monitoring weight loss behaviors during the entire 12 month study period. Receive materials w/ tips and strategies for healthy eating and increasing physical activity. Also given wearable activity tracker. First session with the lifestyle coach in person and thereafter by phone. | Ages 18-35  Chart diagnosis of schizophrenia, schizoaffective disorder, bipolar disorder or major depression  Overweight or obese BMI  ≥25  Enrolled for treatment at agency 3 months prior to study recruitment  Have not started taking Clozapine or Olanzapine antipsychotic medications over prior 2 months (dose **Δ** s are allowed)  Willingness to be randomized to either of the two conditions  Able and willing to attend the weekly 60-minute weight management session, participate in the weekly 1-hour physical activity sessions, and use the Facebook and mHealth components of the PeerFIT program   - Medical contraindication to weight loss Medical conditions that do not receive medical clearance by a primary care provider - Major surgery planned or likely to occur within the next 6 months - Prior or planned bariatric surgery - Use of prescription weight loss medication within the past 6 months - 5% or greater weight loss in 3 months prior to baseline - Currently enrolled in another weight reduction program - Pregnant or planning a pregnancy, or breastfeeding during study period - Cognitive impairment sufficient to interfere with participant's ability to provide informed consent, complete study questionnaires, or participate in a group intervention as indicated by a Mini Mental Status Examination score <24 - Active substance use determined to be incompatible with participation in the intervention identified by screening questionnaire that assesses for excessive use according to intake limits by gender   Use of anabolic steroids with the drug taken at least "most days of the week for the previous month" | **Δ** in weight (Time Frame: Baseline, 6 months and 12 months)  **Δ** in 6-minute walk test (Time Frame: Baseline, 6 months and 12 months)  After baseline blood pressure has been obtained, participants are asked to walk measured distance as far as they are able in 6 minutes.  **Δ** in weight loss self-efficacy assessed using the Weight Efficacy Lifestyle (WEL) questionnaire (Time Frame: Baseline, 6 months and 12 months)-20 items designed to measure self-confidence to control weight by resisting overeating in certain tempting situations. Total score used in analyses. Items are scored on a 10-point Likert scale and total score calculated as sum of individual item responses  **Δ** in self-efficacy for exercise behaviors assessed using the Self-efficacy for Exercise Behaviors (SEB) scale (Time Frame: Baseline, 6 months and 12 months)-12-item scale consists of common barriers that might affect participation in exercise. Use the scale 1-5 to describe confidence could exercise in face of these barriers  **Δ** in peer support for health behavior **Δ** assessed using the 24-item Social Provisions Scale (SPS) (Time Frame: Baseline, 6 months and 12 months)-Level of perceived support. The SPS assesses 6 types of support on a 4-point Likert scale.  **Δ** in serum lipids (Time Frame: Baseline, 6 months and 12 months)-Lipids measured using the CardioChek PA Analyzer, a hand held dual testing system that produces values for total cholesterol, LDL, HDL and triglycerides using a multi-panel test strip and a single drop of blood. | N/A | N/A | N/A | N/A | Submitted: June 27, 2016  **Δ**  in serum lipids was added as a current secondary outcome measures. |
| **Comparing Mobile Health (mHealth) and Clinic-Based Self-Management Interventions for Serious Mental Illness**  ^42^  Dartmouth-Hitchcock Medical Center  Patient-Centered Outcomes Research Institute  Completed  Chicago, Illinois, United States, 60613  Dror Ben-Zeev, PhD  NCT02421965 | April 21, 2015  N/A  August 8, 2018 | N/A  Interventional (Clinical Trial)  Randomized  Single (Outcomes Assessor)  N/A  174 participants | The goal is to compare 2 illness self-management interventions for SMI: a clinic-based protocol (Wellness Action Recovery Planning or WRAP) and mHealth smartphone intervention (FOCUS). Participants randomized to receive 12 weeks of WRAP or FOCUS.  Experimental: FOCUS (Smartphone Application)  Smartphone application system to improve illness self-management and facilitate recovery through system initiated and patient initiated real-time assessment.  Active Comparator: WRAP (Wellness Recovery Action Planning)  Clinic-based intervention to improve self-management and facilitate recovery in individuals with serious mental illness. Group sessions with trained facilitators using lectures, group discussion and exercises. | 18+, chart diagnosis of schizophrenia, schizoaffective disorder, bipolar disorder or major depressive disorder  ≤3 on one of three items which comprise Domination by Symptoms factor from Recovery Assessment Scale  Hearing, motor or vision impairment  Received FOCUS or WRAP intervention in the past 3 years | Patient willingness to enroll/initiate the intervention (WRAP/FOCUS) (1st day of attendance of WRAP session or usage of FOCUS application)-Proportion on individuals who commence intervention after allocation measured by electronic tracking records/FOCUS software.  Patient engagement (3 months)-Weeks of WRAP session attended/ weeks FOCUS was used during the 12-week intervention period.  Patient Satisfaction (3 months)-5 item questionnaire on a 7-point scale  Δ in the severity of symptoms (Baseline, 3 months (post-treatment), 6 months (follow up))-Symptom checklist 9-scale, Beck Depression Inventory-2 (BDI-2) scale and Psychotic Symptom Rating Scale (PSYRATS)  Recovery (Baseline, 3 months (post-treatment), 6 months (follow up)-Recovery Assessment Scale (RAS)  Δ in quality of life (Baseline, 3 months (post-treatment), 6 months (follow up)-6-item Quality of Life scale on a 7-point response scale | N/A | N/A | N/A | N/A | N/A |
| **Wellness Monitoring for Major Depressive Disorder (CBN-Well)**  ^103^  Sidney Kennedy  Sidney Kennedy, University Health Network, Toronto  Active, not recruiting  Alberta, Canada  British Columbia, Canada  Ontario, Canada  Sidney H. Kennedy, MD  [NCT02934334](https://clinicaltrials.gov/show/NCT02934334) | October 14, 2016  N/A  April 19, 2018 | N/A  Observational  N/A  N/A  N/A  100 participants | Prospective, longitudinal, observational study aimed at identifying biomarkers of relapse in MDD. Results may help define clinical approach to relapse management. Participants currently responding to an oral antidepressant treatment regimen and/or therapeutic intervention monitored over minimum period of 13 months. Utilizes remote monitoring technology for data gathering.  MDD: Major Depressive Disorder  Other: Observational | Rate of MDD Patients with Near Term Relapse (Time Frame: Baseline up to the one year enrollment period for the last-subject-in)  Relapse defined as 1. MADRS total square ≥ 22 on at least 2 consecutive visits scheduled or unscheduled 2. Hospitalization for worsening of depression 3. Suicidal ideation w/ intent or suicidal behavior | Meet DSM-V criteria for Major Depressive Episode (MDE) in MDD as determined by MINI  MADRS total square of equal to or less than 14  Currently be responding to an antidepressant medication or combination of treatments for MDD  Complete self-reported assessments via a study-specific smartphone (logPad)  Willing to wear GTPX Link, a wrist worn device for duration of study  Axis I diagnosis, other than MDD, that is considered primary diagnosis  Bipolar I or Bipolar II diagnosis lifetime, MDD with psychotic features lifetime, schizophrenia or schizoaffective disorder  Presence of a significant Axis II diagnosis (borderline, antisocial)  High suicidal risk  History of drug or alcohol use with severity of at least moderate or severe according to DSM criteria within 6 months before screening  Presence of significant neurological disorders, head trauma, or other unstable medical conditions  Received an investigational drug or used an invasive investigational medical device within 90 days before screening + currently enrolled in investigational study | N/A | N/A | N/A | N/A | N/A |
| **Patient Management of Depression Through Technology: A Study of Digitally Enabled Engagement**  ^104^  Advocate Health Care  Takeda  Recruiting  Oak Lawn, Illinois, United States, 60453  David Kemp, MD, MS,  NCT03242213 | August 8, 2017  N/A  June 26, 2018 | N/A  Interventional (Clinical Trial) (Clinical Trial)  Randomized  None (Open Label)    40 participants  N/A | Mobile health application for patient self-management of depression to improve patient-provider engagement for patients diagnosed with major depressive disorder.  No Intervention: Usual Care  Active Comparator: Mobile App  Mobile health app. Patients track emotional wellbeing and depression, set up medication reminders, track adherence to medications, record side effects experienced and take surveys/tests to measure cognitive symptoms and depression. | 18-70 years  Diagnosis of major depressive disorder  PHQ-9>5 at baseline  0-14 days start on monotherapy depression medication  Outpatient care provided by participating Advocate Medical Groups  Diagnosis with major psychiatric disorder  Contraindication to use of depression medications  Patients with treatment resistant depression  Imminent risk for hospitalization due to severe depression  History of hospitalization due to major depressive disorder past 3 months  Significant risk of suicide  History of response only to combination or augmentation therapy in current depressive episode | (18 weeks):Patient Activation- Patient Activation Measure  Patient-Provider Engagement- PPES-7  (18 weeks): Depression symptoms  Cognitive Dysfunction  Medication Δ  Quality of life  Health care utilization | N/A | N/A | N/A | N/A | N/A |
| **Effectiveness of a Mobile Texting Intervention for People With Serious Mental Illness**  ^105^  University of Washington  Recruiting  Illinois, United States, 60290  Dror Ben-Zeev, PhD  [NCT03062267](https://clinicaltrials.gov/show/NCT03062267) | August 8, 2017  N/A  June 26, 2018 | N/A  Interventional (Clinical Trial) (Clinical Trial)  Randomized    Single (Outcome Assessor)    52 participants  N/A | MHealth mobile interventionist texting program on illness management for people with serious mental illness (SMI).  No Intervention: Treatment as Usual for 3 months  Experimental: Mobile Interventionist  Participants will exchange text messages with a mobile interventionist throughout the day for 3 months. Mobile interventionist is trained clinician who provides supportive messaging through text message. | 18-99 years  Chart diagnosis of schizophrenia, schizoaffective disorder, bipolar disorder or major depressive disorder  ≤3 on 1 of 3 Patient Activation items  Receiving community-based treatment  Hearing, vision or motor impairment  English reading level below 4th grade | Psychiatric symptoms (Δ from baseline to 3 months and 6 months)-Positive and Negative Syndrome Scale (PANSS) + Beck Depression Inventory  Recovery (Δ from baseline to 3 months and 6 months)  # of hospitalizations per participant | N/A | N/A | N/A | N/A | N/A |
| **Psychotherapeutic Text Messaging for Depression Pilot Study**  ^106^  University of Michigan  Paul Pfeiffer, University of Michigan  Completed  Ann Arbor, Michigan, United States, 48109  Paul Pfeiffer  University of Michigan  [NCT02872454](https://clinicaltrials.gov/show/NCT02872454) | August 19, 2016  N/A  August 19, 2016 | N/A  Interventional (Clinical Trial) (Clinical Trial)  N/A  None (Open Label)  N/A  190 participants | Demonstrate the feasibility of recruiting/enrolling participants from general population of US adults + deliver a text messaging intervention for depression. Determine whether there are differences in perceived helpfulness of messages from different psychotherapeutic treatment modalities and causes these differences.  Experimental: Text Messaging  Each week, participants will receive daily text messages from 1 of 3 randomly assigned psychotherapeutic modalities-cognitive restructuring, behavioral activation, and techniques based on Acceptance and Commitment Therapy (ACT) for a total of 12 weeks. | 18+ and US resident  PHQ-9≤10 | Intervention Feasibility (6 and 12 weeks post-baseline)-Deliver the intervention as a measure of feasibility. Measured by at least 50% of patients complete the screening questionnaire choosing to enroll in the study, at least 70% of participants completing follow-up measures at 12 weeks, and the study meeting its enrollment goal of 250 patients in 12 months.  Perceived Helpfulness of Messages  Δ (6 and 12 weeks post-baseline)  Δ in Depression Symptoms | N/A | N/A | N/A | N/A | N/A |
| **Treating Depression on a Day-to-day Basis: Development of a Tool for Physicians Based on a Smartphone Application**  ^107^  Centre Hospitalier Charles Perrens, Bordeaux  Not yet recruiting  Bordeaux, France, 33076  David Misdrahi, MD  [NCT03678194](https://clinicaltrials.gov/show/NCT03678194) | September 19, 2018  N/A  September 24, 2018 | N/A  Interventional (Clinical Trial) (Clinical Trial)  Randomized  Single (Outcomes Assessor)  200 participants  N/A | Use mHealth smartphone application to better understand the day-to-day symptomology in depression, medication adherence and treatment efficacy to maximize patient care.    Experimental: Smartphone Application  Receive mobile support system and conventional treatment for 6 weeks. The smartphone application will evaluate symptomology, medication and adherence daily.  No intervention: Standard services  Conventional treatment only. Standard services for depression. | 18-65 years  DSM-IV criteria of depression assessed by Structured Clinical Interview  Started antidepressant treatment less than 5 days before inclusion  Treated in an outpatient setting  Current mental psychiatric impairment or disease that required psychotropic medicine or inpatient treatment on a psychiatric ward  History of psychosis and major depressive disorder with psychotic features  Cognitive deficit | A greater clinical response in the active group (smartphone application) comparatively to the comparator group (Baseline (pre-treatment) and 8 weeks post baseline)-HDRS Hamilton Depressive Rating Scale. Δ in HDRS scores of at least 50% at 8 weeks.  Improvement in scores for therapeutic alliance ((pre-treatment) and 8 weeks post baseline)  Improvement in scores for medication adherence (8 weeks after enrollment)  Improvement in scores for Quality of Life | N/A | N/A | N/A | N/A | Original Primary Outcome Measures (submitted: September 18, 2018) was comparison between the two groups of the number of participants with a decrease in HDRSS scores of at least 50% at 8 weeks. Current Primary Outcome Measures (submitted: September 20, 2018) is a greater clinical response in the active group (smartphone application) comparatively to the comparator group (clinical response was defined as a decline in HDRS-17 score greater than 50%). |
| **Mental Health Telemetry for Self-Management in Major Depressive Disorder (MHTV)**  ^37^  Sunnybrook Health Sciences Centre  Canadian Institute of Health Research (CIHR)  Completed  Toronto, Ontario, Canada  David M. Kreindler, MD  [NCT01999010](https://clinicaltrials.gov/show/NCT01999010) | December 3, 2018  N/A  January 5, 2017 | N/A  Interventional (Clinical Trial) (Clinical Trial)    Non-Randomized    None (Open Label)    N/A  27 participants | Mental health telemetry (MHT) uses cell phones to collect mood journal data in individuals with major depressive disorder. Electronic journal data can be converted into graphical records, allowing for recognition and evaluation of changes in mental health status.    No Intervention: TAU (“A” stage)  No MHT introduced  Experimental: Choice (“A’” Stage)  Patients ending the MHT arm given choice to continue with MHT for two months or resume TAU for remaining two months.  Experimental: MHT (“B” stage)  Software for Mental Health Telemetry (MHT). Record symptom intensity, hospital/ER visits, etc.  Visualize MHT data + MHT entries once daily at pre-determined time. | 14+  DSM–IV diagnosis of MDD  Own web-enabled smartphone  Self-disclosed illiteracy  Blindness  Inability to be successfully trained in MHT | Δ in self-management ratings (2 months and 4 months)-three sub-scale scores (maintenance, management and confidence) of the Sunnybrook Self-Management Scale-Depression (3S-D) instrument at the end of the TAU phase (2 months) vs. End of active treatment phase (4 months)  Δ in Quality-of-life (QoL) ratings (2 months and 4 months) | N/A | N/A | N/A | N/A | N/A |
| **Using Mental Health Telemetry to Predict Relapse and Re-hospitalization in Mood Disorders (PATH-MOD)** ^38^  Sunnybrook Health Sciences Centre  Completed  Toronto, Ontario, Canada  David M. Kreindler, MD  [NCT01882608](https://clinicaltrials.gov/show/NCT01882608) | June 20, 2013  N/A  April 18, 2016 | N/A  Interventional (Clinical Trial) (clinical trial)    Randomized    None (Open Label)    N/A    33 participants | Mental health telemetry allows patients to use cell phones to track the severity of their mood symptoms over time and enables clinicians to view symptom ratings irl. Assess if MHT can reduce re-hospitalization rates in previously hospitalized patients with mood disorders.  No Intervention: Treatment-as-usual  No active intervention or follow-up. Readmission events monitored over 6-month interval via chart review and updates from clinician.  Experimental: Mental health telemetry  Provide daily symptoms self-report using MHT. Readmission events monitored over 6-month interval via chart review and updates from clinician. | 14+  Diagnosis of DSM-IV mood disorder  Long-term follow-up at Sunnybrook  2 lifetime hospitalizations for mood disorders at time of recruitment  Own web-enabled cell phone  Willing to start or continue w/ mood journaling using either MHT or other platform  Be successfully trained to use MHT  Clinician in Department of Psychiatry at Sunnybrook providing majority of healthcare, participating in the study | Rehospitalization rates (6 months)  Quality-of-life (6 months)-Quality-of-life in Bipolar Disorder (QoL.BD) self-report scale, completed at entry and bi-monthly  Relapse rate (6 months)  Participation rate (At Baseline)  Uptake rate (3 months)  Reporting rate (6 months) | N/A | N/A | N/A | N/A | Submitted: November 21, 2014  Quality-of-Life in Bipolar Disorder was added as a Primary Outcome Measures. |
| **Effectiveness of a mHealth Intervention for the Treatment of Depression in People With Diabetes or Hypertension in Peru (LATIN-MHPeru)** ^40^  Universidad Peruana Cayeteano Heredia  University of Sao Paulo General Hospital  National Institute of Mental Health (NIMH)  Northwestern University  Active, not recruiting  Peru  Jaime Miranda, Dr.  [NCT03026426](https://clinicaltrials.gov/show/NCT03026426)   |  | | --- | | January 20, 2017  N/A  October 12, 2018 | N/A  Interventional (Clinical Trial)  Randomized  Single (Investigator)  N/A  432 participants | 6-week low-intensity psychological intervention (CONEMO – CONtrol EMOcional) delivered by a smartphone application to people with depressive symptoms and comorbid diabetes and/or hypertension recruited in primary health care centers and public hospitals in Lima, Peru.  Experimental: CONEMO  Receive a smartphone with CONEMO, an application with 18 sessions that are delivered 3x/week for 6 weeks. High risk of suicide and/or PHQ-9 score  ≥20 referred to the system for follow up. Participants w/ lower levels of depressive symptoms receive recommendation going to mental health professional  Behavioral: CONEMO  Participants offered behavioral activation based intervention delivered by an application for smartphones (CONEMO) oriented to encourage be more activate and incorporate more activities in everyday life. Nurses train participants use CONEMO, make phone calls non-adherence, provide technical support when necessary.  No-intervention: Control Group  Enhanced usual pare. Participants higher risk of suicide and/or PHQ-9 score ≥20 referred to system for follow up. Lower levels of depressive symptoms receive recommendation going to mental health professional. | 21+  Presenting depressive symptoms (PHQ9 ≥10)  Clinical diagnosis of diabetes and/or hypertension  If pregnant, diagnosis of hypertension or diabetes not related to current pregnancy  Moderate or severe suicide risk (Level B2 or C measured by S-RAP) | Proportion of participants with a reduction of 50% or more in the Patient Health Questionnaire (PHQ-9) score at the 3-month assessment [Time Frame: 3 months after inclusion]  Presence and severity of depressive symptoms is measured with the PHQ-9. A reduction of 50% or more in the PHQ-9 score at the 3-month assessment as compared to the PHQ-9 score at baseline will be considered as treatment success.  Proportion of participants with a reduction of 50% or more in the PHQ-9 score at the 6-month assessment [ Time Frame: 6 months after inclusion ]Improvement in scores for Quality of Life measured by the EQD5 at the 3- and 6-month assessment [ Time Frame: 3 and 6 months after inclusion ]Q-5D-standardized instrument that investigates 5 dimensions: mobility, self-care, usual activities, pain/discomfort, and anxiety/depression. Proportion of participants who improve adherence to diabetes or hypertension medications, evaluated by the Morisky questionnaire at the 3- and 6-month assessments [ Time Frame: 3 and 6 months after inclusion ] Proportion of participants who improve on social functioning at the 3- and 6-month assessments compared to the baseline. [ Time Frame: 3 and 6 months after inclusion ]- WHO-DAS 2.0 12-item Interviewer-administered version in Spanish at the 3- and 6-month assessments after inclusion and compared to baseline. Cost-effectiveness: Number of medical consultations, hospitalizations, and visits to the health care team [ Time Frame: 3 and 6 months after inclusion ]Information about medical consultations, hospitalizations, and visits to the health care team will be obtained with a standardized questionnaire and cost-effectiveness analysis will be conducted, Proportion of participants who improve level of activity at the 3- and 6-month assessments [ Time Frame: 3 and 6 months after inclusion ], Behavioral Activation for Depression Scale (BADS), 9-item scale used to measure the frequency of activation and avoidance behaviors hypothetically underlying depression mechanisms. | N/A | N/A | N/A | N/A | N/A |
| **Smartphone-enabled Health Coaching Intervention for Youth Diagnosed with Major Depressive Disorders** ^31^  York University  Centre for Addiction and Mental Health  Recruiting  Toronto, Ontario, Canada  Paul G. Ritvo, PhD  [NCT03406052](https://clinicaltrials.gov/show/NCT03406052) | January 23, 2018  N/A  January 23, 2018 | N/A  Interventional (clinical trial)  Randomized  Triple (Care provider, investigator, outcomes assessor)  168 participants  N/A | Randomized controlled trial comparing youth diagnosed with major depressive disorder treated with online mindfulness-based cognitive behavioral therapy vs. standard psychiatric care.  Experimental: Smartphone-Assisted MB-CBT  Online intervention accessed through smartphone comprised of Mindfulness-Based Cognitive Behavior content.  Behavioral: Smartphone-Assisted MB-CBT  Mindfulness-based CBT online software program workbook accessible online. Exposure to and interaction w/ online workbook combined w/ health coaching (duration of 24 total hours). Each participant will receive a Fitbit-HR Charge. | 18-30 y/o  First Nations background or other ethnicity  BDI-II at mild-moderate levels  Diagnosis of major depressive disorder  <18 yrs and >30 yrs  BDI-II in severe range ≥ 29 or < 14  Does not qualify for diagnosis of major depressive disorder | Beck Depression Inventory (Time Frame: Baseline, 3 months, 6 months)  **Δ** from baseline self-report of symptoms of depression  Beck Anxiety Scale (Time Frame: Baseline, 3 months, 6 months)  Quick Inventory of Depressive Symptomology  Hamilton Depression Rating Scale (HRDS-24)- **Δ** from baseline interview and interviewer rating of symptoms of depression.  Five-Facet Mindfulness Questionnaire (Time Frame: Baseline, 3 months, 6 months)- **Δ** from baseline self-report of mindfulness experience  Brief Pain Inventory (Time Frame: Baseline, 3 months, 6 months)- **Δ** from baseline self-report of pain intensity. | N/A | N/A | N/A | N/A | N/A |
| **Testing the Value of Smartphone Assessments of People with Mood Disorders**  ^108^  Mindstrong, Kasima Neuropsychiatry Institute  Recruiting  La Jolla, California, USA, 92037  Paul Dagum, MD PhD, Mindstrong  David Feifel, MD PhD,  [NCT03429361](https://clinicaltrials.gov/show/NCT03429361) | February 12, 2018  N/A  August 15, 2018 | N/A  Observational  N/A  N/A  40 participants  N/A | Identify the best smartphone data featured (based on keyboard, sensor, voice/speech data) that correlate with mood, anxiety and cognitive assessments in patients with MDD and BD. Identify the best smartphone data features (based on keyboard, sensor, voice/speech) that predict relapse and remission in MDD or BD.  6 month open, exploratory study that follows longitudinally patients with MDD or BD receiving outpatient treatment. Screening and assessment during the week prior to treatment and follow-up at regular intervals for at least 3 weeks and no more than 6 months following treatment. Assessments will be completed by the patient, by a trained clinical rater and by a significant other. Mindstrong application (app) remain on participant’s mobile phone for 6 months. | 18-65 years  Diagnosis of MDD or BD  Montgomery-Asberg Depression Rating Scale (MADRS) ≥ 26; Patient Health Questionnaire (PHQ-9) ≥ 15; Clinical Global Impression (CGI) ≥  4 at screening  Undergoing ketamine treatment at the Kadima clinic or determined by the Kadima clinic to be eligible for ketamine treatment and agrees to receive such treatment prior to participating in the study  Female currently pregnant or planning pregnancy within 6 months  Any current Axis I DSM-5 disorder other than MDD or BD  Any other clinically significant medical condition or circumstance that could interfere with the study | Hamilton Depression Rating Scale (HAM-D) (Time Frame: 6 months)  Repeated measures, within subject design using exploratory techniques that will best identify best smartphone usage features or combination of features that correlate with or predict **Δ** on the HAM-D at post treatment and follow up.  Cognitive Battery (Time Frame: 6 months)  Cognitive battery includes self-report emotional referent task, face-morph task, dot-probe task, choice reaction time, forward digit span, Trails A&B, digit symbol substitution test, delayed memory recall, Stroop, Concerts Continuous Performance Test, 2-back test. Repeated measures, within-subject design using exploratory techniques will identify the best smartphone usage features or combination of features that correlate w/ or predict **Δ** on the Cognitive Battery at post-treatment and follow-up.  Hamilton Anxiety Rating Scale (HAM-A) (Time Frame: 6 months)  Patient Health Questionnaire (PHQ-9) (Time Frame: 6 months)  Defined as PHQ-9 >20 and/or 67% reduction in PHQ-9 improvement between baseline and post treatment or follow-up, at post treatment and follow up. | N/A | N/A | N/A | N/A | N/A |
| **Evolution of Dark Ideas When Introducing or Switching an Antidepressant (DEPASSE)** ^109^  University Hospital, Montpellier  Not yet recruiting  Montpellier, France 34295  Lucile Villain, MD  [NCT03327974](https://clinicaltrials.gov/show/NCT03327974) | November 1, 2017  N/A  November 1, 2017 | N/A  Interventional (clinical trial)  N/A  None (Open Label)  103 participants  N/A | Assess with an ecological momentary assessment method the evolution of black ideas over 30 days following the introduction or switch of an oral antidepressants. Each patient will attend a total of 3 scheduled visits, which will be completed over a period of 1 month (inclusion, between 10-15 days, 1 month. During this month, patient will complete daily assessments through smartphone application “Depasse.”)  Arm: Depressed patients  Ecological momentary assessment through smartphone and 3 scheduled visits. All patients are depressed patients  Other: DEPASSSE application  Daily monitoring on symptoms allowing regular evaluation through modules on a scale from 1-5. The 10 parameters evaluated are mood, dark ideas, sleep, stress, concentration, social activities, energy, pleasure, motivation and libido. | 18-65 years  Diagnosis of a characterized depressive episode  Depression score MADRS >20  Introduction or switch of an antidepressant w/o combination of molecules  French language  Psychiatric comorbidities: Bipolar disorder, schizophrenia, alcohol dependence and/or any other substance objectivized during clinical evaluation  Depressive episode characterized w/ mixed component, psychotic, melancholic or catanoc characteristics  MADRS ≥ 5 to one of the items | Values of the Likert scale assessing dark ideas from 1 (=not at all) to 5 (=often) (Time Frame: 1 month)  Evolution of dark ideas over 30 days. Through DEPASSE smartphone application, patient assess actual frequency of dark ideas on Likert scale.  Values of the Likert scale assessing the actual mood (Time Frame: 1 month)  Evolution of mood over 30 days. Using DEPASSE smartphone application, patient evaluates their motivation from 1 (=not at all) to 5(=absolutely).  Values of the Likert assessing the ability to feel pleasure as usual  Values of the Likert scale assessing the energy  Values of the Likert scale assessing social activities  Values of the Likert scale assessing concentration  Values of the Likert scale assessing stress  Values of the Likert scale assessing libido  Values of the Likert scale assessing sleep  Score at the Montgomery-Asberg Depression Rating Scale (MADRS) -Link between self-evaluation of the patient and evaluation conducted by psychiatrist.  Score at the Columbia Suicide Severity Rating Scale (CSSRS)  Number of emergency room visits-link between the evolution of dark ideas over 1 month and the # of emergency room visits.  Number of hospitalizations-link between the evolution of dark ideas over 1 month and the number hospitalizations  Satisfaction score on the Likert scale-assessment of the patient satisfaction regarding utilization of DEPASSE | N/A | N/A | N/A | N/A | N/A |
| **IntelliCare: Artificial Intelligence in a Mobile Intervention for Depression and Anxiety (AIM)**  ^110^  Northwestern University  Completed  Chicago, Illinois, United States 60611  David C. Mohr, Ph.D.  [NCT02176226](https://clinicaltrials.gov/show/NCT02176226) | June 27, 2014  October 29, 2018  October 29, 2018 | N/A  Interventional (Clinical Trial)  N/A  None (Open Label)  N/A  105 participants | Intelligent treatment system that uses learning approaches within a mobile intervention application to treat MDD and anxiety. Intellicare uses a mobile application to continuously collect patient data and adapt intervention content and motivational messaging to create individualized treatment system. Use smartphone application for 8 weeks and provide feedback at weeks 4 and 8. IntelliCare system consists of apps with lessons and tools designed to teach skills for mood management. Suggested patients use mobile phone tools every day. Each week, participants receive brief motivational intervention from a coach, available to participants via email throughout 8 week study.  Experimental: 8-week Single Arm Field Trial  Use of IntelliCare program for 8 weeks.  Behavioral: IntelliCare  Behavioral interventions for depression and anxiety via a mobile application, IntelliCare. | 19+ y/o  Meets criteria for clinically significant symptoms of depression or anxiety using self-report measures used in screening for depression + interviewer administered measures to ensure symptoms have been present for at least 2 weeks  Any psychiatric condition for which participation in clinical trial of psychotherapy would be inappropriate or dangerous  Exhibits severe suicidality, including ideation, plan and intent  Currently receiving psychotherapy or planning to receive psychotherapy during 8 weeks of the study  Been on an antidepressant or anxiolytic medication for less than 2 weeks and intend to have medication optimized will wait for screening until meet 2 week criterion | 1) Patient Health Questionnaire- 9 (PHQ-9)-Depression Severity Module (Time Frame: Baseline, Week 4 and Week 8)-Measures degrees of depression severity on range of scores from 0-27.  2) Measures degree of depression severity from range of scores 0-27.GAD-7 (Generalized Anxiety Disorder Scale-7) Self-administered 7 item instrument. Total score for the 7 items ranges from 0 to 21.  3) Mean Number of Treatment App Use Sessions by Study Week (Time Frame: Weekly for 2 Months) | 8-week Single Arm Field Trial  Use of IntelliCare program for 8 weeks. Behavioral interventions for depression and anxiety via a suite of mobile phone applications, IntelliCare. | N/A | N/A | 1)Baseline=12.5 (4.3). 96 patients analyzed.  Week 4=8.4 (4.1). 94 patients analyzed.  Week 8=6.4 (4.3). 94 patients analyzed.  2)Baseline=10.9 (4.5). 96 patients analyzed.  Week 4=7.1 (3.9). 94 patients analyzed.  Week 8=5.8 (4.0). 94 patients analyzed.  3) Week 1= 20.09 (15.63). 98 participants analyzed.  Week 2= 22.79 (16.76). 97 participants analyzed.  Week 3=24.1 (20.21). 97 participants analyzed.  Week 4=25.33 (20.86). 94 participants analyzed.  Week 5=26.07 (20.41). 93 participants analyzed.  Week 6=26.23 (23.66). 91 participants analyzed.  Week 7=23.44 (22.27). 89 participants analyzed.  Week 8=23.3 (25.57). 90 participants analyzed.  Week 9= 23.3 (25.57). 90 participants analyzed. | Original primary outcome measures (submitted: June 25, 2014) were changes in depression severity, adherence to mobile application intervention and changes in anxiety. Current Primary Outcome Measures (submitted: February 14, 2018) are the PHQ-9 and GAD-7. Original Secondary Outcome Measures was participant satisfaction. Current Secondary Outcome Measures is the Mean Number of Treatment App Use Sessions by Study Week. |
| **Augmenting Hospitalization for Serious Mental Illness: Cognitive Bias Modification**  ^111^  Mclean Hospital  Not yet recruiting  Belmont, MA, USA, 02478  Courtney Beard, PhD  [NCT03509181](https://clinicaltrials.gov/show/NCT03509181) | April 26, 2018  N/A  April 26, 2018 | N/A  Interventional (clinical trial)  Non-randomized  None (open label)  16 participants  N/A | Develop an augmentation to psychiatric hospital care (I-Change) that can be continued at home following discharge. Targets interpretation bias, or the tendency to resolve ambiguous situations negatively. Interpretation bias is a well-established cognitive vulnerability for psychopathology and is associated with poor emotion regulation, rumination, symptom severity and suicidal ideation.  Behavioral: I-Change  Smartphone delivered word-sentence association task that encourages a healthier interpretive style. | 18+  Currently receiving partial hospital care at the study site  Moderate symptom severity (PHQ-9 or GAD-7 score > 10)  A minimal level of interpretation bias (,50% accuracy on the Word Sentence Association Paradigm)  Current psychiatric symptoms that would prevent understanding of research procedures (e.g. active symptoms of psychosis, mania)  Current/active suicidal ideation (PHQ-9 >1) | Clinical Global Improvement Scale (CGIS) (Time Frame: Through study completion (3 months following discharge from hospital))-clinician administered improvement rating  5-item Work and Social Adjustment Scale-assesses interference caused by patient’s symptoms in work, home management, leisure and family relationships.  Quality of Life Enjoyment and Satisfaction Questionnaire (Time Frame: Through study completion (3 months following discharge from hospital))  Patient Health Questionnaire-9 item  7-item Generalized Anxiety Disorder Questionnaire | N/A | N/A | N/A | N/A | N/A |
| **Evaluation of Text Message Engagement Support of Mindfulness Smartphone Applications**  ^112^  Northwestern University  Recruiting  Chicago, Illinois, USA, 60611  Elizabeth C Adkins, MA  Stephen Schueller, PhD  [NCT03633682](https://clinicaltrials.gov/show/NCT03633682) | August 16, 2018  N/A  August 16, 2018 | N/A  Interventional (clinical trial)  Randomized  None (Open Label)  40 participants  N/A | Assess the usability of 2 mindfulness smartphone applications and to evaluate whether text message support can promote engagement with those apps through a 4 week trial comparing support vs. no support in a population with depression and anxiety.  Experimental: Engagement Support  Participants will be sent text messages that encourage use of app through tips and reminders.  Behavioral: Headspace-Mediation and Mindfulness App  1-month full access to the Headspace meditation app. Listen to at least 1 meditation/day over 4 week study period. Mindfulness is the ability to intentionally and nonjudgmentally observe thoughts, bodily sensations or feelings in the present moment. Targeting areas of stress, anxiety, compassion and sleep.  Behavioral: Stop, Breathe and Think-Meditation and Mindfulness App  1-month full access. Listen 1 meditation/day over 4 week study period.  No Engagement Support  No text message support | 18+  Meet criteria for clinically significant distress caused by anxiety defined by a GAD-7 >10 or clinically significant distress caused by depression PHQ-9>10  18+  Past or current diagnosis of a psychotic disorder, bipolar disorder, dissociative disorder, substance or alcohol abuse dependence  Suicidal, defined as >1 on PHQ-9 | **Δ** in Depression at Week 2 and Week 4 (Time Frame: Baseline, Week 2, Week 4)-PHQ-9 (depression) and GAD-7 (anxiety)  App Usage and Satisfaction (Time Frame: Week 1, Week 2, Week 3, Week 4)-Defined as how many minutes the participant meditations + how many meditations listened to. App satisfaction=participants satisfaction w/ the app. | N/A | N/A | N/A | N/A | N/A |
| **Enhancing Delivery of Problem Solving Therapy Using SmartPhone Technology**  ^113^  VA Office of Research and Development  Completed  Little Rock, Arkansas, USA, 72114  Kathleen M Grubbs, PhD  [NCT01891734](https://clinicaltrials.gov/show/NCT01891734) | July 3, 2013  September16, 2016  November 28, 2016 | N/A  Interventional (Clinical trial)  Randomized  None (Open Label)  N/A  33 participants | Experimental: Problem Solving Therapy plus Moving Forward  All veterans will receive a standard 6 session administration of Problem Solving Therapy. Session 1 will take place in person during a scheduled appointment and will last for 1 hour. Subsequent sessions over the phone for 30 mins. Also receiving Moving Forward app for smartphones, which was adapted from problem solving therapy. Phone content matches problem solving therapy. 24-hours accessibility of psychoeducational materials and worksheets.  Active Comparator: Standard 6 session administration of problem solving therapy. Session 1 in person during scheduled appt for 1 hour. Subsequent sessions over the phone for 30 minutes. | 18-64 years  Patients in the Central Arkansas Veterans Healthcare System Primary Care-Mental Health Integration  Current diagnosis of depression (major depressive disorder, dysthymic disorder, depressive disorder not otherwise specified)  And/or anxiety (panic disorder w/ or w/o agoraphobia, specific phobia, social phobia, OCD, posttraumatic stress disorder, acute stress disorder, GAD, anxiety disorder not otherwise specified)  Any current suicidal ideation  Substance dependence diagnosis and current use  Any psychotic spectrum diagnoses | 1.Depression Anxiety and Stress Scale (DASS) (Time Frame: 6 weeks, 12 weeks)  2.Short Form Health Survey-12-Veterans (SF-12 V) Mental Composite Score (Time Frame: 6 weeks, 12 weeks)  3.Client Satisfaction Questionnaire (CSQ-8) | Problem Solving Therapy Moving Forward: Veterans in each group received 6 sessions of Problem Solving Therapy. Session 1 took place in person immediately after the eligibility assessment for 1 hour. Subsequent sessions took place over the telephone for 30-45 minutes. As part of each PST session, participant completed the PHQ-9 to evaluate ongoing treatment effects and write questions about safety and suicidality. As part of therapy, participant completed home work (psychoeducation about stress, information on effective problem solving and worksheets using the Moving forward app.  Problem Solving Therapy: Same but utilized the Moving Forward workbook instead of the Moving Forward app. | 1.17 participants analyzed for Problem Solving Therapy-Moving Forward  DASS-Depression week 6=6.14(5.76)  DASS-Anxiety week 6= 7.21 (4.53)  DASS-Stress week 6= 11.29 (6.59)  DASS-Depression week 12=6.14 (5.76)  DASS-Anxiety week 12=7.21 (4.53)  DASS-Stress week 12=11.29 (6.59)  16 participants analyzed in Problem Solving Therapy  DASS-Depression week 6=5.23 (5.15)  DASS-Anxiety week 6=5.62 (4.59)  DASS-Stress week 6=8.23 (5.31)  DASS-Depression week 12=5.23 (5.15)  DASS-Anxiety week 12= 5.62 (4.59)  DASS-Stress week 12=8.23 (5.31)  2. 15 participants analyzed for Problem Solving Therapy Plus Moving Forward. SF-12 V-Mental Composite Score 6 weeks=40.00(6.77) SF-12 V Mental Composite Score 12 weeks=39.92 (8.93)  16 participants analyzed for Problem Solving Therapy  SF-12 V-Mental Composite Score 6 weeks=37.19 (7.50) SF-12 V Mental Composite Score 12 weeks=40.15 (10.89)  3. 17 participants analyzed for problem solving therapy plus moving forward. Client Satisfaction Questionnaire (CSQ-8)=27.87 (3.36) 16 participants analyzed for problem solving therapy.  CSQ-8=29.06 (2.62) | N/A | N/A | Original Primary Outcome Measures (submitted: June 28, 2013) was Depressio, Anxiety and Stress, an instrument that measures clinical indices of depression and anxiety as well as acute stress. Current Primary Outcome Measures (submitted: July 27, 2016) is the Depression Anxiety and Stress Scale (DASS). |
| **Technology Assisted Programs that Promote Mental Health for Teenagers (ProjectTECH)** ^32^  Northwestern University  Completed  Chicago, Illinois, USA, 60611  David Mohr  [NCT01912729](https://clinicaltrials.gov/show/NCT01912729) | July 31, 2013  N/A  October 11, 2016 | N/A  Interventional (clinical trial)  Non-randomized  Single (Outcomes Assessor)  N/A  41 participants | Behavioral intervention technologies (BITs) for the prevention of depression in adolescents. BITs=interventions that use information and telecommunications technologies such as the internet, mobile or traditional phones, computers and/or other technologies to support and deliver psychological and behavioral interventions.  Experimental: Networked Peer Support w/ Peer Guide  Have access to tools and lessons based on CBT through a mobile phone application. Access to a private social network that connects them with other participants in the study. Social network moderated by a trained peer coach.  Experimental: Networked Peer Support w/ Clinician Coach  Have access to tools and lessons based on CBT through a mobile phone application. Access to a private social network that connects them with other participants in the study. Social network moderated by a clinician coach.  No Intervention: Wait List Control  May be asked to wait for up to 8 weeks until minimum group size is met. After 4 weeks from baseline, if a group has not yet started, conduct another assessment. | Score of 12-39 (males)/15-39 (females) on the Center for Epidemiologic Studies Depression Scale (CES-D) or reported past month use of marijuana, cigarettes, alcohol or other substances on the Center for Disease Control Youth Risk Behavior Survey (YRBS)  Familiar w/ use of computers, internet, mobile phones  14-19 y/o  Currently taking an antidepressant medication or has taken 1 in the previous 3 months  Visual, hearing, voice or motor impairment that would prevent completion of study procedures  Diagnosis for which participation in this trial is either inappropriate or dangerous.  Is severely suicidal | Depressive Symptoms (Time Frame:  **Δ**  from baseline to up to 8 weeks)  **Δ**  in self-reported depressive symptom severity from baseline to end of treatment.  Adherence to the application (Time Frame:  **Δ**  from baseline to up to 8 weeks)  Number of times accessed and time to last use | N/A | N/A | N/A | N/A | Original Primary Outcome Measures (submitted: July 30, 2013) were Depression (Time Frame: Throughout participation, lasting up to 26 weeks) as measured by the CES-D and MINIKid and Usability of the Program as measured by the USE survey. |
| **New Technologies for Cognitive Behavior Therapy (CBT) Treatment of Adolescent Depression**  ^36^  National Institute of Mental Health (NIMH)  Center for Psychological Consultation  Completed  Madison, Wisconsin, USA, 53717  N/A  NCT01868867 | June 5, 2013  N/A  February 3, 2016 | N/A  Interventional (clinical trial)  Randomized  None (Open Label)  N/A  18 participants | Examine the use of new technologies in disseminating and improving CBT treatment of adolescent depression. An online therapist training tutorial will be followed by 12 weeks of CBT treatment according to the training protocol. CBT treatment will be augmented w/ use of automated text messages for homework reminders + reinforcement of learning. In session patient education and review of CBT concepts will be augmented through teaching materials delivered via iPad.  Experimental: Intervention  Receives online training and text messaging for treatment.  No Intervention: TAU  Similar duration. Intervention provided following TAU period. | 13-18 y/o  Adolescents w/ a DSM-5 mood disorder and a minimum score of 11 on the QUIDS  Bipolar disorder  Severe Conduct Disorder  Moderate to severe alcohol or substance use disorder  Autism spectrum disorder  Any psychotic disorder  Severe suicidal/homicidal ideation or behavior requiring in-patient treatment | QUIDS Quick Inventory for Adolescent Depression (Time Frame:  **Δ**  from baseline to 12 weeks)-assesses clinical domains of adolescent depression  Clinical Global Impressions (CGI) (Time Frame: end of 12 weeks) | N/A | N/A | N/A | +1 Paper Submitted | N/A |
| **Study of Technology-assisted Treatment of Adolescent Depression (iTAD)**  ^33^  iHope Network, Inc  Woburn Pediatric Associates  UConn Health  Completed  Connecticut, USA  Massachusetts, USA  Steven E Locke, MD  Thomas J. McLaughlin, ScD  NCT01582581 | April 20, 2012  N/A  April 30, 2015 | N/A  Interventional (clinical trial)  Randomized  Single (Participant)  N/A  45 participants | Evaluate a computer guided, telephone based therapy for adolescent depression, delivered in a pediatric primary setting in the community.  Active Comparator: Telephonic CBT  Computer guided CBT delivered by a clinician administered telephone intervention. 15-18 sessions lasting 30-45 minutes at weekly intervals.  Sham Comparator: Wait List Control  Randomized wait list control with measurement of study outcomes at week 0, 3 and 5 after the initiation of waiting | 12-17 y/o  Diagnosis of MDD  Ability to receive care as an outpatient  Ability to participate in at least 1 session by phone per week for approximately 12-16 weeks  Pediatrician impression of normal IQ for developmental level  Current or past diagnosis of BD, severe conduct disorder, pervasive developmental disorders or thought disorder  Current treatment w/ CBT  Confounding medical condition  Dangerousness to self or others if they have been hospitalized for dangerousness within 3 months of consent  Deemed to be “high risk” b/c of a suicide attempt requiring medical attention within 6 months of consent or clear intent or active plan to commit suicide  History of self-harm, suicidal attempts or suicidal ideation  Specialty care for substance abuse | Changes in measures of depression over the course of treatment among teens with Major Depressive Disorder (Time Frame: 12 weeks)-PHQ-9  Evidence of program efficacy as indicated by changes in subjects’ knowledge of depression (Time Frame: 12 weeks)-DKT  Evidence of program efficacy as indicated by development of self-efficacy skills-SSEQ  Evidence of program efficacy as indicated by the establishment of therapeutic alliance-Therapeutic Alliance Scale for Adolescents  Evidence of program efficacy as indicated by fidelity and treatment quality-CTR  Evidence of program efficacy as indicated by program acceptance-acceptance questionnaire | N/A | N/A | N/A | N/A | Original Secondary Outcome Measures (submitted: April 19, 2012) was preliminary indicators of program efficacy (Time Frame: 12 weeks) measured by the 1.Depression Knowledge Test 2. Skill Self Efficacy Questionnaire 3.The Therapeutic Alliance Scale for Adolescents 4. The Cognitive Therapy Scale 5.Acceptance Questionnaire |
| **Mobile Phone Sensing and Outreach as Adjuncts to Internet Based Behavior Intervention for Depression**  ^114^  Northwestern University  Completed  Chicago, Illinois, USA  David Mohr  NCT01107041 | April 20, 2018  N/A  March 24, 2017 | N/A  Interventional (clinical trial)  N/A  None (Open Label)  N/A  2010 participants | Evaluating the use of phone sensor data to estimate behavior, depression and anxiety. Participants will include people who are high on depression, high on anxiety, high on both depression and anxiety and health controls. Participants will load software on their phones that collects phone sensor data and obtains self-report on sleep, mood and social contacts. Data will be collected for at least 6 weeks.  Experimental: Mobilyze!  Mobilyze! Delivers behavioral intervention for depression via a mobile phone, interactive website and email. | 19+ y/o  PHQ-9 score 10 or higher  Has an email account, computer and broadband access to the Internet  Hearing or voice impairment preventing participation in psychotherapy  Visual impairment that would prevent use of the website, mobile phone application and assessment materials  Has any psychiatric conditions for which participation in a clinical trial of psychotherapy may be either inappropriate or dangerous  Exhibits severe suicidality | Depression, as assessed the PHQ-9 (Time Frame: Baseline, Weeks 2-6)  Positive Affect (PANAS-Positive Affect Scale) (Time Frame: Baseline, Weeks 2-6)  Anxiety (GAD-7) (Time Frame: Baseline, Weeks 2-6) | N/A | N/A | N/A | N/A | Original Primary Outcome Measures (submitted: April 19, 2010) was Depression, as assessed by Quick Inventory of Depressive Symptoms, PHQ-9 and the Mini International Neuropsychiatric Interview Major Depressive Disorders Module (Time Frame: Measured as baseline, 4 and 8 weeks)  Original Secondary Outcome Measures (submitted: April 19, 2010) was Utilization-Adherence markers for the mobile phone (eg-number of responses to prompts for information) the website (Time frame: Measured from baseline to 8 weeks), Positive Affect, Anxiety (GAD-7) and Health-Related Quality of Life. |
| **Effectiveness of a Technology Assisted Behavioral Intervention in Assisting People with Major Depressive Disorder**  ^34^  Northwestern University  National Institute of Mental Health (NIMH)  Completed  Chicago, Illinois, USA, 60612  David Mohr  NCT00719979 | July 22, 2008  N/A  March 19, 2014 | Interventional (clinical trial)  Randomized  Single (Outcomes Assessor)  N/A  102 participants | Develop and evaluate the effectiveness of technology assisted behavioral intervention, consisting of internet based CBT combined with telephone and email support, in reducing depressive symptoms and improving treatment adherence in PCP with MDD.  Experimental: iCBT and TeleCoaching  Received the technology assisted behavioral intervention (iCBT + Telecoaching). TABI will include 12 weeks of internet based CBT (iCBT) and email support from a coach.  Experimental: iCBT (MoodManager)  Received internet based CBT only. ICBT=access to an interactive web based intervention, designed to teach and facilitate the use of cognitive behavioral skills. Intervention will last 12 weeks.  Active Comparator: TAU/ Wait list control  TAU (standard treatment from participants PCP) For wait list control, participants were not provided any intervention for 6 weeks, after which they were allowed to choose coach or self-directed moodManager. | 19+ y/o  DSM-IV diagnosis of MDD as assessed using the MINI  Has a telephone, email account, computer and broadband access to the Internet  Hearing or voice impairment  Visual impairment  Meets criteria for dementia  Diagnosed w/ a psychotic disorder, BD, dissociative disorder, current substance abuse or other diagnosis for which participation in a clinical trial of psychotherapy may be either inappropriate or dangerous  Currently receiving individual psychotherapy or planning to receive psychotherapy for 12 week treatment phase of the study  Exhibits severe suicidality | Depression (Time Frame: Measured at baseline and Weeks 6 and 12 (post treatment))-PHQ-9 and diagnosis using MINI  Utilization and Attrition (Time Frame: Baseline-week 12)-# logins  Patient satisfaction (Satisfaction Index- Mental Health) (Time Frame: Measured at baseline and weeks 6 and 12) | N/A | N/A | N/A | N/A | Original Primary Outcome Measures (submitted: July 18, 2008) was Depression, as assessed by HRSD, PHQ-9 and SCID and Attrition (in arms with i-CBT)  Original Secondary Outcome Measures was i-CBT utilization (e.g., number of logins, average visit length, total time spent on the site, number of exercises completed), Health-Related Quality of Life (SF-36V) and Patient Satisfaction (Satisfaction Index-Mental Health) |
| **Technology Enabled Mental Health Intervention for Individuals in the Criminal Justice System**  ^115^  Jail Education Solutions, Inc  University of Illinois at Chicago  Not yet recruiting  N/A  NCT03105973 | April 10, 2017  N/A  April 12, 2017 | N/A  Interventional (clinical trial)  None (Open Label)  N/A  65 participants  N/A | Build and test a technology enabled mood disorder treatment intervention for individuals who are incarcerated.  Experimental: Open Label Trial Arm  4 weeks of technology enabled CBT treatment. CBT based modules delivered via a tablet over 4 weeks. | 18+ y/o  Reside in general Population pod of the Allegheny County jail | Beck Depression Inventory (BDI) (Time Frame: 4 weeks)-inventory of depression items  Generalized Anxiety Disorder-7 (GAD-7) (Time Frame: 4 weeks)  PTSD Checklist-Civilian Version (PCL-17) (Time Frame: 4 weeks)  User satisfaction-users (Time Frame: 4 weeks)  User satisfaction-correctional facility staff (Time Frame: 4 weeks) | N/A | N/A | N/A | N/A | N/A |
| **Training and Supervision Program for Depression Management**  ^39^  University of Chile  Completed  Santiago, Chile  Graciela Rojas Castillo, MD  NCT02232854 | September 5, 2014  N/A  October 25, 2017 | N/A  Interventional (Clinical Trial)  Randomized  Single (Outcomes Assessor)  N/A  256 participants | Test whether a Comprehensive Technology Assisted Training and Supervision Program will improvement depression management in Primary Health Care clinics in Santiago, Chile.  Experimental: Depression training/supervision program  Primary Health care team training in depression, a focus group after training, telephone monitoring of patients and web based supervision of clinicians.  No Intervention: Usual Care  Receive all interventions that are guaranteed for persons with depression in Chile: treatment in Primary Health clinics with the Primary Health Care team and referral to the regional specialized psychiatric service, | 18-65 y/o  Current depressive episode, according to the MINI  Current depression treatment  No access to telephone | **Δ**  from Baseline Depressive Symptomology at 3 months (Time Frame: Baseline, 3 months)-PHQ-9 after patient recruitment  **Δ**  from Baseline Depressive Symptomology at 6 months (Time Frame: Baseline, 6 months)-PHQ-9 at 6 months after patient recruitment  Adherence to depression treatment at patient’s level (Time Frame: 3 and 6 months after baseline)  **Δ**  from Baseline quality of life at 3 and 6 months (Time Frame: Baseline, 3 months, 6 months)-measured with SF-36.  **Δ**  from Baseline Clinical Outcomes at 3 and 6 months- CQ-45.2  Use of Health Care services at patient’s level (Time Frame: 3 and 6 months after baseline)  Rate of treated depressed cases at Primary Care team level (Time Frame: 12 months before randomization and 12 months after) | N/A | N/A | N/A | N/A | N/A |
| **Online Peer Networked Collaborative Learning for Managing Depressive Symptoms (MoodTech)**  ^35^  Northwestern University  Completed  Chicago, IL, USA 606111Re  David Mohr  NCT02841787 | July 22, 2016  October 29, 2018  October 29, 2018 | N/A  Interventional (clinical trial)  Non-randomized  None (Open Label)  N/A  47 paicipants | Tailor and test a web-based intervention in adults 65+ y/o. Pilot a novel intervention to examine methods to improve adherence to web based interventions.  Experimental: Individual Internet Intervention (III)  Coached internet intervention based on principles of CBT for depressed older adults delivered individually by 2 clinical psychologists (iCBT for late life depression without social network included)  Experimental: Internet Intervention+ Peer Supp (II+PS)  Coached internet intervention based on principles of CBT for depressed older adults delivered w/ peer support; group moderate was provided by 2 clinical psychologists.  No intervention: Waitlist Control (WLC)  Waiting period, no intervention administered. WLC participants received access to the III following the 8 week waiting period. | 65+ y/o  Has elevated depressive symptoms  Has a telephone, email account, computer and broadband access to the internet  Has basic internet skills and is able to access the internet independently  Hearing or voice impairment that would prevent participation in psychotherapy  Visual impairment that would prevent completion or assessment materials  Is diagnosed with a psychotic disorder, BD, dissociative disorder, current substance abuse or other diagnosis for which participation in a clinical trial of psychotherapy may be either inappropriate or dangerous  Is currently receiving individual psychotherapy or planning to receive psychotherapy during the 8 week treatment phase of the study, exhibits severe suicidality, has initiated treatment with an antidepressant in past 14 days | 1.Patient Health Questionnaire-9 (PHQ-9)-2.Depression Severity Module (Time Frame: Baseline and Week 8-Difference in PHQ-9 score)  3.Mean Number of Sessions Across the 8-week Trial (Time Frame: 8 weeks)  System Usability Scale (SUS) (Time Frame: 8 weeks)  4.Average Coaching Time Per Participant by Group (Time Frame: 8 weeks)-average time spent on messages and calls and on group moderation. | N/A | N/A | Results posted on ClinicalTrials.gov | N/A | Original Primary Outcome Measures (submitted: July 20, 2016) was Depression (Time Frame: Baseline to end of treatment) measured as the  **Δ**  in self-reported depressive symptom severity from baseline to end of treatment and adherence to the program measured as the number of times the program is accessed from start to last use from baseline to end of treatment. |

| **ALZHEIMER’S DISEASE AND OTHER DEMENTIAS** | | | | | | | | | |
| --- | --- | --- | --- | --- | --- | --- | --- | --- | --- |
| **Title, Sponsors and Collaborators, Recruitment Status, Location, Investigator, ClinicalTrials.gov Identifier** | **First Posted, Results First Posted, Last Update Posted,** | **Phase, Study Type, Allocation, Masking, Estimated Enrollment, Actual Enrollment** | **Intervention** | **Eligibility Criteria: Inclusion/ Exclusion** | **Primary and Secondary Outcomes** | **Reporting Groups: Description** | **Measures of Adherence: Planned & Posted** | **Reporting of Clinical Trials Outcomes** | **Altered Outcomes** |
| **iCare-AD: A Mobile Health Application for Caregivers of Patients With Dementia** ^11^  Indiana University  Not yet recruiting  Indiana, United States  Richard Holden  NCT03119259 | April 18, 2017  N/A  August 16, 2017 | N/A  Interventional (Clinical Trial) (Clinical Trial)  Randomized  Single (Outcomes Assessor)  448 participants  N/A | To test if mobile health technology w/ Aging Brain Care (ABC) Clinical Program improves behavioral and psychological symptoms of patients suffering from Alzheimer’s disease and related dementias (ADRD) and the distress of their informal caregivers.  Active Comparator: ABC Clinical Programs Only  ABC (Aging Brain Care) team creates individualized care plan with the informal caregiver. For a year, ABC team uses face to face and telephone interactions with the patient and caregiver to monitor/modify the care plan.  Experimental: HABC 2.0 Mobile App Plus ABC  HABC 2.0 software on caregiver’s mobile phone. Manage the behavioral and psychological symptoms of dementia by delivering feedback and support without any intervention from ABC Team while providing longitudinal assessment. | 18+,  Diagnosis of possible or probable Alzheimer’s disease from physician in the ABC Clinical Program  No visual impairment  Patient and caregiver are community dwelling in central Indiana  Willing to participate in the ABC clinical program (including receiving home visits) | Behavioral and Psychological Symptoms of Dementia (BPSD) and Caregiver Distress (12 months)-  Neuropsychiatric Inventory (NPI) and NPI-Caregiver Distress Score  Health care utilization (3, 6, 9 and 12 months)  Caregiver depression  Caregiver burden  Caregiver self-efficacy | N/A | N/A | N/A | N/A |
| **Diabetes as an Accelerator of Cognitive Impairment and Alzheimer’s Disease**  ^12^  Parc Sanitari Pere Virgili  Hospital Universitari Vall d'Hebron (HUVH - VHIR)  Consorci Sanitari de Terrassa (CST)  Fundació Recerca Mútua Terrassa (FMT)  Clínica Universitària de la Fundació Universitària del Bages (FUB)  Althaia Xarxa Assistencial Universitària de Manresa  Fundació Privada Hospital Asil de Granollers (HAG)  Consorci Hospitalari de Vic (CHV)  Lambdaloopers  Universitat Politècnica de Catalunya (UPC)  Leitat  Mixestat  Meditecnologia  Mind the Byte  Not yet recruiting  Marco Inzitari, MD, PhD  Carmina Castellano-Tejedor, PhD  [NCT03578991](https://clinicaltrials.gov/show/NCT03578991) | July 6, 2018  N/A  July 6, 2018 | N/A  Interventional (clinical trial)  Randomized  None (Open Label)  174 participants  N/A | Studying the effects of an eHealth intervention on improving metabolic control and other cardiovascular risk factors as the approach to prevent or delay the process of cognitive impairment + reduce conversion rates to Alzheimer’s disease in a sample of patients diagnosed with type 2 diabetes mellitus with mild cognitive impairment. The smart electronic pillbox will monitor adherence to pharmacological treatment by registering time of medications intake. Incorporate visual and auditory reminders for taking medications. Also have sensors and alarms to ensure the proper preservation of the medicines contained. The interactive digital platform will enable communication b/w patients and caregivers and healthcare professionals. Aims to provide feedback and guidelines on treatment adherence + better optimize treatment on cardiovascular risk factors. Inform about medication dosages and intake schedule, healthy lifestyles, occurrence of adverse events and monitor patients’ glycemic control. Alerts caregivers and healthcare professionals when the patient is hyperglycemic or hypoglycemic.  No Intervention: Arm 1. Control Group TAU.  Type 2 diabetic patients w/ mild cognitive impairment who will receive standard clinical treatment recommended by their PCP/endocrinologist.  Experimental: Arm 2. Intervention-smart pillbox: TAU plus the use of a smart pillbox.  Experimental: Arm 3. Intervention- smart pillbox & interactive digital platform | Diagnosis of diabetes mellitus type 2 with time evolution >5 years  65-85 y/o  Active treatment with hypoglycemic agents  Diagnosed with mild cognitive impairment  Family history of Alzheimer’s disease  Any type of dementia  History of neurological or psychiatric conditions not stabilized that can substantially affect cognition  Severe metabolic or systemic disease that affects to the cognitive state  Treatment w drugs that alter the cognitive state  Limitations of mobility that can avoid/restrict application or evaluation of intervention  Other types of diabetes  Unstable advanced diabetic retinopathy | **Δ** of score obtained in the Repeatable Battery for the Assessment of Neuropsychiatric Status (RBANS) (Time Frame: At baseline and an average of 18 months after enrollment)-detection of cognitive disorder in degenerative and nondegenerative pathology. Evaluates 5 functions, by means of 12 subsets.  Cardiovascular risk factor: Hypertension (Time Frame: At baseline and an average of 18 months after enrollment)-Systolic and diastolic assessed.  Cardiovascular risk factor: Obesity-BMI combined with height and weight  Cardiovascular risk factor: Dyslipidemia  Usual medication  Presence of micro and macrovascular complications  Biomarkers (serum and DNA)  Short Physical Performance Battery test  Hachinski Scale (HS)  Montreal Cognitive Assessment (MOCA)  Mini-mental state examination (MMSE)  Degree of diabetes control (Time frame: at baseline, at 9 and an average of 18  months after enrollment)  Lipid profile and renal and hepatic function  Presence and severity of hypoglycemia (Time Frame: at baseline, at 4, 9, 13 and an average of 18 months after enrollment)  Blessed Dementia Rating Scale (Time frame: at baseline, at 4, 9, 13 and an average of 18 months after enrollment)  Geriatric Depression Scale  Schwab and England Activities of Daily Living Scale  Memory failure in everyday life questionnaire (MFE)  Morisky Medication Adherence Scale (MMAS-8)  Functional and Social Support Questionnaire (DUKE-UNC-11) | N/A | N/A | N/A | N/A |
| **Comparing Smartphone Technology and a Memory Strategy on Improving Prospective Memory in Alzheimer's Disease** ^13^  Baylor University  National Institute on Aging (NIA)  Not yet recruiting  Waco, Texas 76798  Michael Scullin  [NCT03384043](https://clinicaltrials.gov/show/NCT03384043) | December 27, 2017  N/A  December 27, 2017 | N/A  Interventional (Clinical Trial) (Clinical Trial)  Randomized  Double (Care Provider, Outcomes Assessor)  52 participants  N/A | Technology-based intervention to help patients with their daily prospective memory tasks. Prospective memory is memory for future intentions and goals. The loss of prospective memory may result in a decrease in independent functioning. Participants w/ mild cognitive impairment and mild Alzheimer’s disease will use smartphone for 4 weeks  Experimental: Smartphone Personal Assistant  Personal assistant feature of the smartphone (“Cortana”) to provide reminders to do prospective memory tasks at the appropriate time and location.  Active Comparator: Implementation Intention  Memory strategy in which participants verbally state when/where they will perform a prospective memory intention. Participants use the smartphone voice recorder app. | Meet National Institute of Aging-Alzheimer’s Association recommended criteria for very mild to mild stages of Alzheimer’s disease  Clinical Dementia Rating (CDR) of 0.5-1.0  Clinical interview or medical record suggests that psychiatric, vascular, traumatic, movement disorders or other neurodegenerative diseases or medical conditions are causing the cognitive decline | Objective Prospective Memory Performance (Measured for 4 weeks)-  Performance on the experimenter-assigned time-based, event-based and person-based prospective memory tasks (# of tasks completed).  Quality of Life-Positive Affect and Well-Being (Pre-Intervention and Post-Intervention (4 weeks))  Quality of Life-Able to Participate in Social Roles and Activities  Quality of Life-Satisfaction with Social Roles and Activities  Quality of Life-Cognitive Function  Subjective Memory Performance Questionnaire  Perceived Memory Structured Interview  Use of Technology/Strategy (Measured for 4 weeks)  Instrumental Activities of Daily Living  Smartphone Accessibility/Usability Scale  Training Duration (Pre-Intervention) | N/A | N/A | N/A | N/A |
| **Therapeutic Efficacy of Categorical Language Fluency Smartphone Game Application** ^14^  Seoul National University, Bundang Hospital, Seoul National University, Ministry of Science, ICT and Future Planning  Enrolling by invitation  Seong-nam, Korea  Ki Woong Kim, M.D., Ph.D.  NCT02848404 | July 28, 2016  N/A  September 6, 2018 | N/A  Interventional (Clinical Trial) (Clinical Trial)  N/A  None (Open Label)  N/A  20 participants | Purpose is to test therapeutic efficacy of categorical language fluency in mild cognitive impairment and mild Alzheimer’s dementia through the Categorical Language Fluency Smartphone Game Application.  Experimental: Categorical Language Fluency Smartphone Application  Smartphone game application with the intention of training categorical language fluency in 4 weeks training. | ≥55 years and older  Confirmed literacy  Diagnosed to mild cognitive impairment , Clinical Dementia rating of 0 or 0.5  Diagnosed to possible or probable Alzheimer’s dementia by NINCDS-ADRDA and Clinical Dementia Rating (CDR)≤1  Evidence of delirium, confusion  Any neurological conditions causing cognitive decline  Evidence of severe cerebrovascular pathology  History of LOC over 1 hour due to head trauma, repetitive head trauma of mild severity  History of substance abuse or dependence  Presence of depressive symptoms or medical comorbidities that could influence cognitive function or decline | Application usage log (4-weeks treatment)-Log to assess compliance of participant by recording frequency and duration of training by using application.  Subjective memory complaint questionnaire (after 4-weeks treatment)  Geriatric depression scale  Korean version of MMSE for dementia screening | N/A | N/A | N/A | N/A |
| **ArtontheBrain: An Inclusive Evidence-based Cognitive Health App for Older Adults to Promote Aging at Home** ^15^  Baycrest, University of Calgary, Sheridan College  Recruiting  Toronto, Ontario, Canada  Kelly Murphy, Ph.D., C.Psych,  [NCT03551483](https://clinicaltrials.gov/show/NCT03551483) | June 11, 2018  N/A  October 31, 2018 | N/A  Interventional (clinical trial)  Randomized  Single (participant)  110 participants  N/A | Seeks to validate the positive health impacts of a mobile health (mhealth) intervention ArtontheBrain, a web-based app aimed at promoting cognitive health in older adults. ArtontheBrain incorporates three basic activities; learning (history of the artwork), play (telling stories, solving puzzles) and socializing with other users online or in person. It is modeled after participatory arts-based interventions which are associated with health benefits in older adults, like an improved sense of wellbeing, physical health, decreased risk of dementia and reduced need for health services.  Experimental: ArtontheBrain  30-45 minutes 2x/week over 6 weeks  Active Comparator: Seniors Online Victoria  30-45 mins 2x/week over 6 weeks, after which they will participate in the ArtontheBrain intervention.  Waitlist Control: No treatment for 6 weeks, then 6 weeks of the ArtontheBrain intervention | 60+  Normal or corrected to normal vision  Compliance w/t treatment over specified 6 weeks  Having experienced:  Age normal cognitive decline defined as having a Montreal Cognitive Assessment (MoCA) score ­≥23 with no functional impairment in Instrumental Activities of Daily Living (iADLs) and no subjective memory complaint  MCI as defined as having a MoCA score ≤26 with no significant functional impairment in iADLs and report of memory decline by self or family member; or health professional Early dementia as defined as MoCA score ≤23 with significant functional impairment in more than one iADLs domain. Access to computer and internet  Significant vision loss Major psychiatric disorder Neurological disorder causing aphasia or causing severe dementia, Motor limitations that prevent independent use of computer technology, Current history of substance abuse | EQ-5D-5L (Time frame: **Δ** from baseline quality of life at 6 week)-Comprises 5 dimensions of subjectively perceives quality of life in areas of mobility, self-care, usual activities, pain/discomfort and anxiety/depression. Participants report on a 5 point scale. Visual analog scale from 0-100 to assess momentary health state.  Short Warwick-Edinburg Mental Wellbeing Scale-7 item scale designed to measure mental wellbeing over previous 2 weeks on 5 point Likert scale.  Short-Form Health Survey (SF-36)-36 items that measure health across 8 domains.  Standard Health Care Utilization-evaluates individuals of health care utilization occurring in previous 6 months. 4 items of 3 types of health care utilization.  Physical Activity Scale in the Elderly (Time Frame: **Δ** from baseline at 6 weeks)-self-report measure of physical activity based on 10 items by multiplying time spent or participation with empirically derived weights.  Social Engagement Survey  Alternative Uses Task  Digit Span Test  Means-End Problem Solving  Art Engagement Survey  Life Space Questionnaire  Autobiographical Interview  Attention Network Task  Corsi Block Tapping Task | N/A | N/A | N/A | N/A |

|  | | | | | | | | | | |
| --- | --- | --- | --- | --- | --- | --- | --- | --- | --- | --- |
| **ANXIETY DISORDERS** | | | | | | | | | | |
| **Title, Sponsors and Collaborators, Recruitment Status, Location, Investigator, ClinicalTrials.gov Identifier** | **First Posted, Results First Posted, Last Update Posted,** | **Phase, Study Type, Allocation, Masking, Estimated Enrollment, Actual Enrollment** | **Intervention** | **Eligibility Criteria: Inclusion/ Exclusion** | **Primary and Secondary Outcomes** | **Reporting Groups: Description** | **Outcomes Measures:** | **Measures of Adherence: Planned & Posted** | **Reporting of Clinical Trials Outcome** | **Altered Outcomes** |
| **Connection to Care: Pilot Study of a Mobile Health Tool for Patients with Depression and Anxiety** ^116^  University of Washington  Completed  Washington, USA  Amy M Bauer, MD, MS  NCT02497755 | July 14, 2015    December 2, 2017    December 2, 2017 | N/A  Interventional (Clinical Trial) (Clinical Trial)  N/A  N/A  N/A  18 participants | Pilot feasibility study of a mobile health application for PCP patients receiving treatment for depression or anxiety in an integrated primary care-based behavioral health program.  Experimental: Mobile App  Smartphone application to add to their treatment for anxiety and/or depression. Smartphone app will send psychoeducation and reminders to complete self-report data, collect passive data, and provide aggregated information to a provider dashboard. | 8-65 y/o  Be part of Behavioral Health Integration Program for anxiety and/or depression  Actively suicidal as determined by healthcare professional  Score of 3 on PHQ-9 item 9  Documented history of 2 or more suicide attempts  Working diagnosis of psychotic disorder, bipolar disorder, dementia, active substance dependence | 1) App Acceptability as Measured by # of Patient App Users Who Rate App Easy to Use and Time Spent Reasonable (4 weeks after intervention started)-# of patients who rated the app easy to use and amount of time spent using the app as reasonable when asked about app acceptability in qualitative interview and quantitative survey.  2) App Acceptability as Measured by # of Care Manager Dashboard Users Who Rate Dashboard Easy to Use and Time Spent Reasonable (8-16 weeks after final patient participant is enrolled)-# of care managers who agreed that the app dashboard was easy to use and that the amount of time spent using the app dashboard was reasonable when asked about app acceptability and benefit vs. burden of use with regard to clinical workflow in a qualitative interview.  3) App Usefulness as Measured by # of Patient App Users Who Rate App as Useful (4 weeks after intervention started)-# of patients who rated the app as useful to them when asked about app usefulness in a qualitative interview and via a quantitative survey.  4) App Usefulness as Measured by # of Care Manager Dashboard Users Who Rate Dashboard as Useful (8-16 weeks after patient is enrolled)-# of care managers who expressed that app was useful to them with regard to clinical workflow in a qualitative interview.  Technology Acceptability as Measured by the Obtrusiveness Scale for Pervasive Technology  Patient Satisfaction as Measured by the Ginger.io Product Feedback Survey  Patient Use of the App as Measured by Percentage of App Surveys Completed  Care Team Communication as Measured by the Consumer Assessment of Healthcare Providers and Systems (CAHPS) - Communication Scale  Care Process Measures as Measured by the # and Type of Contacts With Care Manager | Patient Mobile App Users: Smartphone app sent psychoeducation and reminders to complete self-report data, collect passive data, and provide aggregated information to a provider dashboard. Participants used the app for at least 4 weeks, with the option to continue for up to 12 weeks. After 4 weeks, conducted phone interview on satisfaction with the app.  Care Manager Dashboard User:  Online dashboard to monitor patient usages and responses to mobile app assessments. Used dashboard for duration of participant involvement in the study. | 1) 16/16  2) 1/1  3)11/16  4) 1/1 | N/A | Posted on ClinicalTrials.gov | Original Primary Outcome Measures (submitted: July 13, 2015) were patient acceptability as determined by qualitative interview, care manager acceptability as determined by qualitative interview, patient report of usefulness as determined by qualitative interview and care manager report of usefulness as determined by qualitative interview. Current primary outcome measures (submitted: October 25, 2017) are app acceptability as measured by number of patient app users who rate app easy to use and time spent reasonable, app acceptability as measured by number of care manager dashboard users who rate dashboard easy to use and time spent reasonable, app usefulness as measured by number of patient app users who rate app easy to use and time spent reasonable and app usefulness as measured by number of care manager dashboard users who rate dashboard as useful. |
| **Improving Medical Care With Electronic Interventions Based on Automated Text and Phone Messages** ^117^  Washington University School of Medicine, Epharmix, Inc  Enrolling by invitation  Saint Louis, Missouri, United States 63110  Will R Ross, MD, MPH  NCT03002311 | December 23, 2016    December 2, 2017    September 25, 2017 | N/A  Interventional (Clinical Trial) (Clinical Trial)  Randomized  None (Open Label)  5000 participants  N/A | Goal is to test the effects of an electronic health intervention developed by Epharmix; 2-way SMS text messages and phone calls.  Experimental: EpX eHealth Intervention  SMS text messaging or phone call reminders of physician prescribed actions or requesting clinical data. Patient can respond to messages. Answers may trigger alert to provider to manage health condition.  Control: TAU as designated by clinical provider. Depending on specific condition, participants may receive placebo version of eHealth intervention. | 5+ y/o  BJC Health Care/ Washington University patients who have medical records  Known phone #  Severe neurological conditions or cognitive disorders | Adherence (Up to 5 years)-Determine if Epharmix automated text and phone reminders improve adherence to physician prescribed activities. Percent increase in adherence to physician prescribed actions (# of completed events of interest divided by # of clinically prescribed events) compared to standard of care and/or placebo.  Patient engagement (Up to 5 years)  Time to COPD special hospitalizations  Δ in hbA1c from baseline  Follow-up appointment adherence  Breastfeeding duration (Up to 6 months postpartum) | N/A | N/A | N/A | N/A | Current Secondary Outcome Measures (submitted: February 10, 2017)-Breastfeeding duration was added as a current secondary outcome measure. |
| **Impact of Preanesthetic Information and Behavioral Intervention Using Smartphone on Anxiety of Children** ^61^  Faculdade de Ciências, Médicas da Santa Casa de São Paulo  Completed  São Paulo, Brazil, 01221010  Ligia Mathias, PhD  [NCT02246062](https://clinicaltrials.gov/show/NCT02246062) | September 22, 2014    January 7, 2016    January 7, 2016 | N/A  Interventional (Clinical Trial) (Clinical Trial)  Randomized  None (Open Label)  N/A  84 participants | To test if the information provided to relatives and/or smartphone application provided to children effects the prevalence and level of child preoperative anxiety.  No intervention: Control group  Relative receive only conventional verbal information before the procedure.  Active Comparator: Info Group  Relative, in addition to conventional verbal information, received a leaflet containing information about anesthetic procedure one day before procedure.  Active Comparator: Smartphone group  Relative received conventional verbal information one day before procedure and child received smartphone application immediately before entering the operating room  Active Comparator: Smartphone and Info Group  Relative received conventional verbal information, leaflet containing information about the anesthetic procedure one day before the surgery and child received smartphone application immediately before entering the operating room. | 4-8 y/o  ASA physical status I, II, III  Undergoing minor-medium elective surgical procedures  Indication of general anesthesia  Not receiving premedication  Psychomotor deficits  Use of psychoactive drugs  Hearing and visual impairment  Previous surgery  For relatives, illness or mental disorder clinically recognized and decline to participate | Impact of Preanesthetic Information and Behavioral Intervention Using Smartphone Application on Anxiety of Children Measure by m-YPAS (24 hours before surgery)-Yale Preoperative Anxiety Scale (m-YPAS)  Impact of Preanesthetic Information and Behavioral Intervention Using Smartphone Application on Anxiety of Children Measure m-YPAS (Immediately before entering operating room)  Impact of Preanesthetic Information and Behavioral Intervention Using Smartphone Application on Anxiety of Children Measure by m-YPAS (Immediately before induction of anesthesia). | Refer to intervention | Impact of Preanesthetic Information and Behavioral Intervention Using Smartphone Application on Anxiety of Children Measure by M-YPAS  Yale Preoperative Anxiety Scale (m-YPAS).  N= 21 in control group with average score  27.8 SD + 10.26  N= 21 in info group with average score  24.18 SD+ 2.12  N= 21 in smartphone group with average score  23.79 SD+ 1.27  N= 21 in smartphone and info group with avg score 24.98 SD+ 6.20  (No statistical analysis provided) | N/A | +1 Paper Submitted | N/A |
| **Evaluating the Psychophysiological Effects of a Smartphone-Based Mindfulness Task** ^68^  York University  Recruiting  Toronto, Ontario, Canada  Joel Katz, PhD  [NCT03296007](https://clinicaltrials.gov/show/NCT03296007) | September 28, 2017  N/A  January 10, 2018 | N/A  Interventional (clinical trial)  Randomized  None (Open Label)  180 participants  N/A | Evaluate the psychophysiological effects of a smartphone based mindfulness meditation app (MMA) for individuals with clinically significant symptoms of major depression and/or anxiety or symptoms of chronic pain. Meditative practice involves paying attention to present moment experiences and focusing on breathing sensations.  Experimental: Mindfulness Meditation App  Use smartphone app to practice mindfulness meditation for 12 minutes.  Active Comparator: Mindfulness Meditation No App  Not use a smartphone app but will receive instructions to practice mindfulness meditation for 12 minutes. | 18+  York University students  Individuals diagnosed cardiac conditions will be excluded due to confounds with heart rate variability assessment | Hear Rate Variability (Time Frame: 22 minutes)-stress biomarker derived from heart rate recordings that reflects parasympathetic control of cardiac output. | N/A | N/A | N/A | N/A | N/A |
| **Effect of Premedication Type on Preoperative Anxiety in Children**  ^62^  Diskapi Yildirim Beyazit Education and Research Hospital  Bahar SAKIZCI UYAR, Diskapi Yildirim Beyazit Education and Research Hospital  Recruiting  Ankara, Turkey  Bahar Sakizci Uyar  [NCT03530670](https://clinicaltrials.gov/show/NCT03530670) | May 21, 2018    N/A    August 15, 2018 | Phase 4  Interventional (Clinical Trial) (Clinical Trial)  Randomized  Single (participant)  138 participants  N/A | To compare the effects of pharmacological and nonpharmacological premedication on preoperative anxiety and mask acceptance after adenotonsillectomy.  Active Comparator: Oral midazolam (demizolam)  To prevent preoperative anxiety premedicated by 0.5mg/kg oral midazolam. In preoperative holding area, in the operating room, while anesthesia induction by m-YPAS. Make anesthesia induction easier while anesthesia induction by Mask Acceptance Scale.  Active Comparator: <http://www.animaturk.com/animasyon/suko-ameliyat-oluyor>.  Prevent preoperative anxiety by watching a short movie in the preoperative holding area in the operating room while anesthesia induction by m-YPAS + easier anesthesia induction by Mask Acceptance Scale  Active comparator: Playing smartphone game in the preoperative holding area in the operating room while anesthesia induction by m-YPAS + easier anesthesia induction by Mask Acceptance Scale | 5-8 years  ASA 1-2  Elective surgery  Chronic diseases  Development disability  Prematurity  Neurological diseases  Psychoactive medication use  Hearing/visual impairment  History of surgery | Δ in operative anxiety (Before premedication, 20 minutes after premedication, in operating room, at anesthesia induction)-mYPAS (modified Yale Preoperative Anxiety Scale)  Emergence agitation (Postoperative every 10 minutes from entrance to the recovery room until discharge)  Mask acceptance score while mask induction (An anesthesia induction) | N/A | N/A | N/A | N/A | Current Primary Outcome Measures (submitted: August 13, 2018)-Mask acceptance (At anesthesia induction) was eliminated as a primary outcome measure and added as a secondary outcome measure. |
| **Using Smartphones to Enhance the Treatment of Childhood Anxiety** ^65^  University of Pittsburgh  National Institute of Mental Health (NIMH)  Completed  Pittsburgh, Pennsylvania, 15213  Jennifer Silk, PhD  [NCT02259036](https://clinicaltrials.gov/show/NCT02259036) | October 8, 2014    N/A  June 6, 2017 | N/A  Interventional (Clinical Trial) (Clinical Trial)  N/A  None (Open Label)  N/A  40 participants | Interactive smartphone app to increase effectiveness of talk therapy such as CBT for child anxiety.  Arm: SmartCAT Enhanced Treatment  Cognitive behavioral therapy + ecological monetary treatment enhancement smartphone app called SmartCAT. Participants answer series of questions about moods and daily experiences. Electronic notification/day and prompted through series of questions that take 5 mins. Participant data sent to therapists to be reviewed weekly-integrate into treatment + customize feedback to the patient | 9-14 years  DSM-IV diagnosis of Generalized Anxiety Disorder (GAD), Separation Anxiety Disorder (SAD), and/or Social Phobia (SP) as identified by the Kiddie Schedule of Affective Disorders.  Requires current ongoing treatment with psychoactive medication others other than stimulants  Actively suicidal or at risk for harm to self or others  Neuromuscular or neurological disorder  Read level <80 on WRAT-4  Comorbid diagnosis  Lifetime diagnosis of autism spectrum disorder, bipolar disorder, psychotic depression, schizophrenia, schizoaffective disorder | PARS Treatment Response (10 weeks)-Pediatric Anxiety Rating Scale  Absence of diagnosis on K-SADS (10 weeks) | N/A | N/A | N/A | N/A | Original Primary Outcome Measures (submitted: October 3, 2014) was the Clinical Global Impression Improvement (CGI-I) defined as treatment response at post treatment. Current Primary Outcome Measures (submitted: January 5, 2016) is the PARS Treatment response. Original Secondary Outcome Measures was the PARS and Screen for Childhood Anxiety Related Emotional Disorders (SCARED). Current Secondary Outcome Measures is the Absence of diagnosis on K-SADS. |
| **Smartphone-Based Exposure Treatment for Dental Anxiety** ^63^  West Virginia University  Enrolling by invitation  Morganton, West Virginia, USA, 26505  Daniel W. McNeil, PhD  [NCT03461016](https://clinicaltrials.gov/show/NCT03461016) | March 9, 2018  N/A  March 9, 2018 | N/A  Interventional clinical trial)  Randomized  None (Open Label)  40 participants  N/A | Utilization of smartphones to improve adherence to self-directed exposure therapy/how presenting phobic material on a smartphone might promote increased adherence in conducting self-directed exposure.  Experimental: Smartphone-Based Exposure Therapy  2 weeks of exposure therapy via smartphone. Participants will have the opportunity to receive up to 50 mins of exposure video intervention daily for 2 weeks.  No Intervention: Waitlist Control  Randomly assigned. Will not receive treatment. After 2 weeks of no intervention, participants in this condition will be offered the same treatment as treatment condition. | 18+  Reporting a low level of discomfort with dental related care | **Δ** in Dental Fear Survey (scale-20 item) (Time Frame: **Δ** from Baseline to 14 days)-participant self-report of dental care related fear and anxiety, range of 20-100  **Δ** in Subjective Units of Distress (**Δ** from baseline to 14 days)-participant self-report of anxiety, scale of 0-100  Acceptability (Time Frame: 14 days)-participant self-report of acceptability of treatment range of 1-48 | N/A | N/A | N/A | N/A | N/A |
| **Youth Mayo Clinic Anxiety Coach Pilot Study** ^66^  Stephen Whiteside  National Institute of Mental Health (NIMH)  Completed  Rochester, MN, USA 55905  Stephen Whiteside, PhD  [NCT02205177](https://clinicaltrials.gov/show/NCT02205177) | July 31, 2014  N/A  August 20, 2018 | N/A  Interventional (Clinical Trial)  Randomized  Single (Outcome Assessor)  N/A  10 participants | Test the feasibility and effectiveness of using the Mayo Clinic Anxiety Coach smartphone app as an addition to traditional therapy for the treatment of anxiety disorders in youth. Mayo Clinic Anxiety Coach=smartphone application based on CBT for anxiety disorders. Scheduled reminders to engage in therapeutic exercise, point of performance support, individually tailored information, real time symptom assessment, readily available communication with therapist.  Active Comparator: Face-to-Face w/ Anxiety Coach  Therapists will provide 6-12 50 minute face to face therapy sessions using Anxiety Coach. Sessions expected to initially occur weekly and be within the office. Therapist expected to utilize Anxiety Coach within the session, encourage patient use application to complete homework and review progress in-session via web-based portal.  Experimental: Minimal Contact w/ Anxiety Coach  Therapist will meet w/ patient and PCP for an initial 50 minute session to provide tutorial on use of Anxiety Coach. Therapist expected to view patient’s progression via web-based portal and communicate w/ patient electronically at least 1x/week total of at least 6 and up to 12 weeks of intervention. Therapists allowed 2 additional face to face 2 sessions if necessary. | 7-17 years  Primary diagnosis of social phobia, separation anxiety disorder, panic disorder w/ or w/o agoraphobia, specific phobia or OCD  Parent or other primary care giver able to participate w/ the child in all assessment and treatment activities  Estimated average intelligence  History of an/or current diagnosis of psychosis, autism, bipolar disorder, mental retardation, oppositional defiant disorder, PTSD, selective mutism, major depressive disorder  Currently suicidality or recent suicidal behavior  Parent unable to adequately participate due to intellectual or psychiatric difficulties  Starting or changing dosage of psychiatric medication last 2 months | Mean **Δ** from Baseline in Pediatric Anxiety Rating Scale (PARS) at Treatment Completion (Time Frame: Within 5 working days of treatment completion)-interview based tool to assess for the presence and severity of anxiety symptoms in children and adolescents utilizing parental and youth input to guide clinician ratings.  Qualitative interview assessing subject safety and treatment adherence (Time Frame: Within 5 working days of treatment completion) | N/A | N/A | N/A | N/A | Current Primary Outcome Measures (submitted: July 30, 2014)-qualitative interview assessing subject safety and treatment adherence (Time frame: within 5 working days of treatment completion) was eliminated as a primary outcome measure. |
| **Youth Mayo Clinic Anxiety Coach Randomized Controlled Trial** ^118^  Stephen Whiteside  National Institute of Mental Health (NIMH)  Completed  Rochester, MN, USA 55905  Stephen Whiteside, PhD, LP  [NCT02205203](https://clinicaltrials.gov/show/NCT02205203) | July 1, 2014  N/A  April 11, 2018 | N/A  Interventional (Clinical trial)  Randomized  Double (Investigator, Outcomes Assessor)  70 participants  N/A | Active Comparator: Face-to-Face w/ Anxiety Coach  Therapists will provide 6 to 12 50-minute face-to-face therapy sessions using Mayo Clinic Anxiety Coach. Sessions expected to initially occur weekly and be within the office, but therapist can leave office to conduct exposure. Therapist expected to utilize Anxiety Coach within the session, encourage patient use application to complete homework + review progress in-session via the web-based portal.  Experimental: Treatment as Usual (TAU)  Therapists provide treatment consistent w/ orientation and clinical judgement. Supportive therapy, relaxation and cognitive restricting. 6 to 12 50-minute face to face therapy sessions in therapists office, with flexibility to leave office for exposure. Therapists can communicate with patients with sessions as long as not the primary mode of treatment | 7-17 years  Primary diagnosis of social phobia, separation anxiety disorder, panic disorder w/ or w/o agoraphobia, specific phobia or OCD  Parent or other primary care giver able to participate w/ the child in all assessment and treatment activities  Estimated average intelligence  History of an/or current diagnosis of psychosis, autism, bipolar disorder, mental retardation, oppositional defiant disorder, PTSD, selective mutism, major depressive disorder  Currently suicidality or recent suicidal behavior  Parent unable to adequately participate due to intellectual or psychiatric difficulties  Starting or changing dosage of psychiatric medication last 2 months | Mean **Δ** from Baseline in Pediatric Anxiety Rating Scale (PARS) at Treatment Completion (Time Frame: Within 5 working days of treatment completion)-interview based tool to assess for the presence and severity of anxiety symptoms in children and adolescents utilizing parental and youth input to guide clinician ratings. | N/A | N/A | N/A | N/A | N/A |
| **ACT-smart: Smartphone-supplemented iCBT for Social Phobia and/or Panic Disorder** ^67^  Stockholm University  Completed  Stockholm, Sweden  Per Carlbring, Professor  [NCT01963806](https://clinicaltrials.gov/show/NCT01963806) | October 16, 2013  N/A  November 17, 2015 | N/A  Interventional (clinical trial)  Randomized  None (Open Label)  150 participants  N/A | Investigate the effect of a transdiagnostic, Internet administered cognitive behavioral (iCBT) self-help program for anxiety, supplemented with a tailored smartphone application. The effect of added therapist support will also be studied. In addition to pre and post treatment measurements, the study includes 2 mid treatment and 2 follow up assessments (12 and 24 months)  Experimental: Smartphone supplemented iCBT with therapist support  Internet administered cognitive behavioral self-help program will be divided into 10 weekly, progressively available modules covering CBt and Acceptance and Commitment Therapy (ACT) conceptualizations of anxiety disorders and more therapeutic techniques. Each module also includes writing exercises. Smartphone applications allows the user to access, create and modify personal database of behaviors. Frequency of carrier out behaviors are recorded. Also allows user to browse and search database of behaviors by other study participants. Behavioral: Therapist support  Provide feedback of written exercises of each treatment module. 15 mins per participant/week  Experimental: Smartphone supplemented iCBT without therapist support  Active Comparator: Active waiting list control group with delayed treatment  Completes measurements at day 24 and 48 and at the end of the initial treatment period, otherwise no activity until crossover. | 18+  DSM-IV-TR criteria for panic disorder and/or social anxiety disorder as assessed by SCID  Daily access to internet via computer and smartphone  Parallel psychological treatment  Non-stable dosage of psychoactive medication during last 3 months  Participants deemed to suffer from suicidal tendencies or other condition requiring specialized treatment | The Generalized Anxiety Disorder 7-item (GAD-7) [ Time Frame: **Δ** from baseline at: [1] day 24 and [2] day 48 into the 10-week treatment period; [3] 0-1 week after the treatment period; follow-ups at [4] month 12 and [5] month 36 after the treatment period. ]  Self-rated Liebowitz Social Anxiety Scale (LSAS-SR) [ Time Frame: **Δ** from baseline at: [1] 0-1 week after the treatment period; follow-ups at [2] month 12 and [3] month 36 after the treatment period. ] Self-rated Panic Disorder Severity Scale (PDSS-SR) 9-item Patient Health Questionnaire (PHQ-9) Quality of Life Inventory (QOLI) | N/A | N/A | N/A | N/A | Current Primary Outcome Measures and Current Secondary Outcome Measures (submitted: November 27, 2013)  All outcomes that listed month 24 after the treatment period in the time frame were changed to month 36 after the treatment period. |
| **Effects of Complementary Therapies Delivered Via Mobile Technologies**  ^64^  University of San Francisco  Fulbright  Completed  Rejkavik, Iceland, 101  N/A  NCT02236455 | September 10, 2014  N/A  September 10, 2014 | N/A  Interventional (clinical trial)  Randomized  None (Open Label)  N/A  105 participants | Determine the effects of complementary therapies delivered via mobile technologies have a therapeutic effect on surgical patients’ anxiety, pain and self-efficacy in healing reports before, following and at 10 day follow up.  Experimental: Audio Relaxation technique  Relaxation is a process that decreases effects of stress on mind and body. Can help cope with everyday stress and stress related to health problems.  Experimental: Medical Music Intervention  Used to assist with relaxation and reduce stress levels in patients.  Experimental: Nature Therapy w/o Music  Ecotherapy=use of nature to reduce stress and increase levels of wellbeing in patients.  Experimental: Nature Therapy w/ Music  Nature therapy videos were produced w/ and w/o music for surgical patients. | 18-75 y/o  English or Icelandic speaking  Cognitively alert and oriented to person, place, time and situation  Intact visual and auditory senses  History of substance abuse  Chronic pain > 6 months  Use of narcotic medication for more than 6 months  Major psychiatric disorders  Taking prescribed medications for thought disorders  Having ophthalmology and/or auditory surgery or impairments | **Δ** from baseline anxiety state via the State Trait Anxiety Scale (STAI) (Time Frame: 10 days)  **Δ** in baseline pain level using the Numeric Rating Scale (NRS) (Time Frame: 10 days) | N/A | N/A | N/A | +1 Paper Submitted | N/A |

| **ALCOHOL USE DISORDERS** | | | | | | | | | |
| --- | --- | --- | --- | --- | --- | --- | --- | --- | --- |
| **Title, Sponsors and Collaborators, Recruitment Status, Location, Investigator, ClinicalTrials.gov Identifier** | **First Posted, Results First Posted, Last Update Posted** | **Phase, Study Type, Allocation, Masking, Estimated Enrollment, Actual Enrollment** | **Intervention** | **Eligibility Criteria: Inclusion/Exclusion** | **Primary and Secondary Outcome Measures** | **Reporting Groups: Description** | **Measures of Adherence: Planned & Posted** | **Reporting of Clinical Trials Outcome** | **Altered Outcomes** |
| **The Effectiveness of a Smartphone Application in the Treatment of Alcohol Use Disorder**  ^69^  St. Patrick’s Hospital, Ireland  Recruiting  Ireland  Conor Farren  NCT03396887 | January 11, 2018  N/A  January 11, 2018 | N/A    Interventional (Clinical Trial)    Randomized (Intervention or Control)    None (Open Label)  50 participants    N/A | U Control Drink is a smartphone application in aiding recovery for patients with AUD. The application consists of supportive messages, computer-based CBT, a drinking log, activities and trigger log, craving management, and gamification.  Experimental: U Control Drink Smartphone Application- Use of a smartphone application and treatment as usual for three months. Smartphone application delivers text 2x a day, 12 sessions of prevention cognitive behavioral therapy.  Control Group: To serve as an active comparator, the control group receives treatment as usual. | alcohol use disorder diagnosis, alcohol treatment program participation, 18+ y/o, MSSE greater than 25, iPhone or android phone  younger than 18 or older than 70, psychotic disorder, history of alcohol use disorder (or current), no smartphone | # of drinking days Timeline to Follow-Back Scale (descriptive questionnaire using calendar), (3 months) Timeline to Follow-Back Scale (descriptive questionnaire using calendar), app activity score- generated from the app (3 months), units of alcohol consumed per drinking day- Timeline to Follow-Back (3 months)  (3 months): time to first drink, Δ in the Alcohol Use Disorders Identification Test score, Δ in The Alcohol Abstinence Self-Efficacy Scale scores, Δ in The Obsessive-Compulsive Drinking Scale scores, Δ in the Beck Depression Inventory scores, Δ in the Beck Anxiety Inventory scores. | N/A | N/A | N/A | N/A |
| **AlcoChange: An Open Label Pilot Study of Smartphone Monitoring for Alcoholic Liver Disease** ^70^    Royal Free Hospital NHS Foundation Trust  Not Recruiting    N/A  Gautam Mehta  NCT03474328 | March 15, 2018    N/A    August 31 , 2018 | N/A    Interventional (Clinical Trial)    N/A    None (Open Label)    60 participants    N/A | AlcoChange is a smartphone app with a breathalyzer that allows patients to self-monitor and incorporates basic life support in regard to triggers.  AlcoChange: a smartphone app and breathalyzer facilitating self-monitoring used for 3 months to determine compliance and changes in self-reported alcohol consumption. | presence of ARLD, ages 18-70, intent to maintain abstinence, compatible smartphone  inability to provide consent | self-reported alcohol consumption (average/week)- timeline follow back method (3 month)  compliance with the application (3 months) | N/A | N/A | N/A | N/A |
| **Health Mobile Cognitive Stimulation in Alcoholics** ^71^    Pedro Gamito, Universidade Lusófona de Humanidades e Tecnologias  Unknown  N/A  Pedro Gamito  NCT01942954 | September 16, 2013    N/A  September 16 , 2013 | N/A    Interventional (Clinical Trial)    Randomized    Single (Outcomes Assessor)    N/A    54 participants | A mobile health technology is used as a neuropsychological intervention program by providing patients with cognitive stimulation.  Experimental: Mobile Health Cognitive Stimulation- Use of mobile health technology to deliver cognitive stimulation for four weeks on a three day/ week basis.  Control Group: TAU for alcohol abstinence according to the Minnesota Model. | 18-75 y/o, patients who scored higher than cutoff values for their age on Mini Mental examination Test  dependency from substance other than alcohol or with history of previous neurological disorders | Δin the results of Frontal Assessment Battery (FAB)- Frontal Lobe Cognitive Functioning (retention, attention and calculation, language and visual-spatial abilities (retention, attention and calculation, language and visual-spatial abilities) (before cognitive rehabilitation - after cognitive rehabilitation 1month/10 sessions) | N/A | N/A | N/A | N/A |
| **mWELLCARE: An Integrated mHealth System for the Prevention and Care of Chronic Disease (mWELLCARE)** ^119^    Public Health Foundation of India, Wellcome Trust, London School of Hygiene and Tropical Medicine    Completed  India  Dorairaj Prabhakaran, Vikram Patel  NCT02480062 | June 24, 2015  N/A  October 2, 2017 | N/A    Interventional (Clinical Trial)    Randomized    None (Open Label)    N/A    3702 participants | m-WELLCARE is a smartphone application that provides patient health profiles, decision support for clinical care and monitoring and feedback for use in health centers with SMS follow-up.  Experimental: mWELLCARE software arm- Intervention Includes a software application loaded on a tablet and used by care coordinators to register patients with hypertension or diabetes to generate clinical decision support recommendations and track patients overtime to improve follow up care.  Active Comparator: TAU.  [AUD was considered one of the chronic diseases evaluated in the outcomes.] | 18-75 y/o, patients who scored higher than cutoff values for their age on Mini Mental examination Test  dependency from substance other than alcohol or with history of previous neurological disorders | (12 months): systolic blood pressure- difference in mean Δ in systolic blood pressure between two treatment arms, glycated hemoglobin (HbA1C) - difference in mean Δ in glycated hemoglobin between two treatment arms  (12 months): depression, smoking, BMI, alcohol use, fasting blood sugar, total cholesterol, CVD risk, Cost | N/A | N/A | N/A | Changed September 12, 2016: no longer tracking 10 year risk of CHD and added tracking for alcohol use, fasting blood sugar, total cholesterol, CVD risk, and cost |
| **Study of Mobile Phone Delivered Intervention to Reduce Alcohol Consumption (mROAD)** ^72^    University of Southern California    Completed  Los Angeles, California  Elizabeth Burner  NCT02158949 | June 9, 2014  N/A    June 3, 2015 | N/A    Interventional (Clinical Trial)    Randomized    None (Open Label)    100 participants    N/A | mROAD is an intervention through mobile phone test messaging to reduce alcohol use for people with AUD.    Experimental: mROAD- Intervention includes 1 week of texts 2x a day modeled after Screening, Brief Intervention, and Referral to Treatment intervention.  No Intervention: TAU in ED | AUDIT score 15-20, mobile phone w text capability  age < 18, unable to consent, language other than English or Spanish | (1 week): acceptability of intervention to patient- percentage of patients that do not opt out messages prior to 1-week completion  feasibility of follow up of urban low-income at-risk drinkers by phone (1 month and 3 months, | N/A | N/A | N/A | N/A |
| **Smartphone Based Continuing Care for Alcohol** ^120^  University of Pennsylvania    Recruiting  Pennsylvania, United States  James R, McKay  NCT02681406 | February 12, 2016    N/A    October 11, 2017 | N/A    Interventional (Clinical Trial)    Randomized    None (Open Label)    280 participants    N/A | ACHESS is a smartphone-based application to treat AUD and is paired with telephone monitoring and counselling (TMAC). ACHESS provides automates 24/7 support while TMC provides regular continued contact with the same counselor and no support in between.  Experimental: Telephone Monitoring and Counseling- Participants receive 20-minute telephone counselling calls once a week, then biweekly, etc. for 12 months.  Experimental: ACHESS- Participants use an addiction-based smartphone application that connects them anonymously to a social network of other people in the study.  Experimental: TMC + ACHESS- Participants receive both interventions.  No intervention: Treatment as Usual | DSM-V diagnosis, 3 weeks of IOP, 18-75 y.o, name + phone # of two contacts, functionally literate, read smartphone, willingness to be randomized  current psychotic disorder or dementia, acute medical problem requiring inpatient treatment, participating in other forms of treatment for substance abuse, unable to read/comprehend informed consent | (18 months): Timeline Follow Back (percent % of heavy alcohol use) | N/A | N/A | N/A | N/A |
| **Project Guard: Reducing Alcohol Misuse/Abuse in the National Guard** ^73^    Joseph Calabrese, MD, , Case Western Reserve University, University of Michigan, University of Toledo Health Science Campus  Enrolling by Invitation  Ohio, United States  Frederic C Blow, PhD  NCT02860442 | August 9, 2016    N/A    September 19, 2017 | N/A  Interventional (Clinical Trial)  Randomized    None (Open Label)  750 participants    N/A | The SP-BI intervention is designed to target behaviors of AUD and a personalized review of participants’ goals/value, feedback regarding their alcohol use and consequences, and a decisional balance exercise in order to formulate a change of plan.  Experimental: Behavioral Smartphone Brief Intervention (SP-BI)- Participants will receive 20-30-minute intervention through an app program based on the Feedback, Advice, Menu of Options, Empathic clinical behaviors, and Support of Self-efficacy regarding making changes format.  Placebo Comparator: Enhanced TAU- Participants will receive an informal brochure with resources available to members of the military related to mental health and alcohol use. | 3-month AUDIT-C score of >5 (men) or >4 (women), current member of Ohio National Guard  No access to a smartphone, active treatment for substance use disorders | (baseline,3-,6-,and 12-months post baseline): Δ in # of drinking days- AUDIT-C and TLFB, Δ in # of drinks/day - AUDIT-C and TLFB, Δ in # of binge drinking days- AUDIT-C and TLFB  (baseline,3-,6-,and 12-months post baseline): frequency of illicit drug use between SP-BI and EUC conditions | N/A | N/A | N/A | Updated August 8, 2016: No changes |
| **A Text Message Behavioral Intervention to Reduce Alcohol Consumption in Young Adults (TRAC)** ^74^  University of Pittsburgh  Completed  Pennsylvania, United States  Brian P Suffoletto  [NCT01688245](https://clinicaltrials.gov/show/NCT01688245) | September 19, 2012  N/A  May 27, 2015 | N/A  Interventional (Clinical Trial)  Randomized  Double (Investigator, Outcomes Assessor)  N/A  765 Participants | TRAC uses SMS messaging to deliver health prevention interventions.  Experimental: SMS Assessments and Feedback- Participants receive weekly (pre-weekend) intention and post weekend outcomes assessment paired with personalized feedback and harm-reduction support.  Active Comparator: SMS Assessments- The participants receives weekly post-weekend drinking outcome assessments.  No intervention: Control- No SMS Dialog | AUDIT-C score of 3 or more for women and 4 or more for men  Current treatment for psychiatric disease, any prior treatment for drug or alcohol use disorder | (30 days): Timeline Follow-Back Procedure  (3 months): Injury Behavior Checklist | N/A | N/A | +1 Paper Submitted  *1 Paper Submitted | N/A |
| **A Tailored Physical Activity Smartphone App for Patients With Alcohol Dependence** ^75^  Butler Hospital, National Institute on Alcohol Abuse and Alcoholism (NIAAA)  Recruiting  Rhode Island, United States  Anna M. Abrantes Ph. D  [NCT02958280](https://clinicaltrials.gov/show/NCT02958280) | November 8, 2016  N/A  January 23, 2018 | N/A  Interventional (Clinical Trial)  N/A  None (Open Label)  30 Participants  N/A | The Fit&Sober application aims to help patients in early recovery from Alcohol Use Disorders increase their levels of physical activity.  App Development and Open Pilot:  Phase 1- The prototype of the app is being developed with usability studies from patients with AUDs  Phase 2- Patients will be in a 12-week open pilot trial (n=20) to test the feasibility and acceptability of the application. | Between 18-65 y.o. DSM-5 criteria for AUD, sedentary lifestyle, engaged in alcohol treatment  DSM-5 diagnosis of moderate/severe substance use disorder, history of psychotic disorder, suicidality, mania, organic impairment, physical problems, pregnancy | (12 weeks): Minutes of moderate-vigorous physical activity per week (accelerometry)  (12 weeks): Satisfaction with app (system usability scale), app usage (metadata collected from app) | N/A | N/A | N/A | N/A |
| **Young Adult Naturalistic Alcohol Study (YANAS) Using Smartphone Technology in a Stimulated Laboratory Environment** ^76^  University of Florida  Recruiting  Florida, United Stated  Robert F Leeman, PhD  [NCT02841735](https://clinicaltrials.gov/show/NCT02841735) | July 22, 2016  N/A  December 22, 2017 | N/A  Observational  Cohort  N/A  129 participants  N/A | The goal of this observational study is to compare different types of mobile technology for their effects on alcohol drinking and compare their usability ratings among young adults.  Smartphone breathalyzer device and app: Participants can blow into the smartphone breathalyzer after each alcoholic drink and the readings can inform their following decisions.  BAC estimator app: Participants can make an entry into the BAC estimator app which produces estimated BAC and can inform subsequent decisions.  Text Messaging: Participants will send a text a study smartphone each time they have an alcoholic drink. | Read English and complete assessments, drink alcohol regularly  Positive urine test for use of certain illegal drugs, undergraduate student, graduate student at University of Florida, pregnant | (during an alcohol drinking session): quantity of alcohol consumption (compare forms of mobile technology for their effects) | N/A | N/A | N/A | N/A |
| **Feasibility of a Smart-phone Based support System for Hazardous Drinkers (NZStepAway)** ^121^  University of Auckland, Waitemate District Health Board, University of Alaska Anchorage  Recruiting  New Zealand  Natalie Walker, PhD  [NCT03553056](https://clinicaltrials.gov/show/NCT03553056) | June 12, 2018  N/A  June 12, 2018 | N/A  Interventional (Clinical Trial)  Randomized  Triple (Participant, Investigator, Outcomes Assessor)  200 participants  N/A | Step- Away is a smartphone delivered health intervention meant to help individuals moderate or abstain from drinking alcohol.  Experimental: Intervention (NZ Step Away App)- Participants will have access to 10 modules of the app that they use daily (drinkers profile, goal setting, rewards, cravings, strategies, support, reminders, high risk times, moods, activities)  Active Comparator: Control (Modified App)- Participants will only have access to 2 modules: drinkers profile and goals setting | Reside in Auckland, 18+, smartphone, currently drink alcohol, at least 2 episodes of binge drinking, criteria for hazardous drinking, motivation to moderate or abstain  Moderate to severe alcohol dependence, enrolled in an alcohol program, another person in household is participant | (6 months post-randomization): # of days spent hazardously drinking in last 30 days  (1 months, 3 month, 6 months post-randomization): # of days spent hazardously drinking, drinks per day, drinking related problems (SIP-R), contact with substance abuse related health services, who have they talked to about drinking, serious adverse events, app utilization, withdrawal from study, loss to follow up, alcohol withdrawal, medical help sough for withdrawal signs/symptoms, | N/A | N/A | N/A | N/A |
| **Smartphone Technology: Young Adult Drinking (STEADY)** ^77^  University of Florida, National Institute on Alcohol Abuse and Alcoholism (NIAAA)  Recruiting  Florida, United States  Robert F Leeman, PhD  [NCT02963818](https://clinicaltrials.gov/show/NCT02963818) | November 15, 2016  N/A  June 25, 2018 | N/A  Interventional (Clinical Trial)  Randomized  None (Open Label)  109 participants  N/A | The goal of this study is to compare different types of mobile technology for their effects on alcohol drinking and compare their usability ratings among young adults.  Smartphone breathalyzer device and app: Participants can blow into the smartphone breathalyzer after each alcoholic drink and the readings can inform their following decisions.  BAC estimator app: Participants can make an entry into the BAC estimator app which produces estimated BAC and can inform subsequent decisions.  Text Messaging: Participants will send a text a study smartphone each time they have an alcoholic drink. | Read English and complete assessments, drink alcohol regularly  Positive urine test for use of certain illegal drugs, undergraduate student, graduate student at University of Florida, pregnant | (during one alcohol drinking session, an avg of 2 hours): quantity of alcohol consumption (compare forms of mobile technology)  (two-week field project): quantity of alcohol consumption (compare forms of mobile technology) | N/A | N/A | N/A | N/A |
| **Smartphone-paired Breathalyzers and Loss- and Gain-framed Texts for Reducing Drinking and Driving (BESAFE)** ^78^  University of Pennsylvania  Completed  Pennsylvania, United States  Kit Delgado, MD, MS  [NCT03335735](https://clinicaltrials.gov/show/NCT03335735) | November 8, 2017  N/A  August 8, 2018 | N/A  Interventional (Clinical Trial)  Randomized  Single (Participant)  N/A  58 participants | This study aims to demonstrate feasibility of scalable behavioral intervention by using smartphone paired breathalyzers and test messages to reduce drinking and driving among individuals.  Experimental: Loss-Framed Test Messages- Participants receive loss-framed text messages relating to drinking and driving on Thursday-Saturday related to loss of personal freedom, loss of money, loss of employment due to DUI conviction. The messages are related to loss aversion (better not to lose than to gain).  Experimental: Gain-Framed Test Message- Participants receive gain-framed text messages relating to drinking and driving on Thursday-Saturday related to saving lives, gaining control, making loved ones happy. The messages are related to how the participants gains from taking preventative action.  No Intervention: Control- Participants receive no information. | Ages 21-39, one heavy drinking day/week, valid photo ID, willing to use Uber or Lyft, drives 4x or more, owns apple iPhone or android  Desire/received alcohol treatment, severe AUD per DSM-V, non-English speaking, pregnant, people who should not consume alcohol due to medical condition | (8 weeks): Δ proportion of breathalyzer measurements submitted with self-reported drinking episodes  (8 weeks): Δ frequency of BACtrack monitoring, driving and drinking episodes in which BAC via self-report or BAC measure is positive (driving monitoring app passively running on phone), Δ in accuracy of BAC guess vs actual BAC. | N/A | N/A | N/A | N/A |
| **Adaptive Goal-Directed Adherence Tracking and Enhancement (AGATE)** ^79^  Talaia, Inc, National Institute on Alcohol Abuse and Alcoholism (NIAAA)  Completed  New Mexico, United States  Susan Stoner, Ph.D  [NCT01349985](https://clinicaltrials.gov/show/NCT01349985) | May 9, 2011  N/A  May 13, 2014 | N/A  Interventional (Clinical Trial)  Randomized  None (Open Label)  N/A  136 Participants | The AGATE adherence tracking and enhancement system uses text messaging and internet capabilities to address medication adherence.  Experimental: AGATE- All participants will be prescribed naltrexone 50mg 1xdaily for 8 weeks.  Active Comparator: SASED- All participants will receive a web based alcohol and side effects diary via smartphone and will be prescribed naltrexone 50mg 1x daily for 8 weeks. | Problem or heavy drinkers, 21-55 y.o, greater Albuquerque NM area, interest in stopping/reducing drinking, candidates for naltrexone pharmacotherapy  Participation in other naltrexone study, unable to operate smartphone, significant psychiatric or physical illness, drug dependence, opioid use, lifetime dependence | (8 weeks): medication adherence (medication event monitoring system, pill counts, self-report indices)  (8weeks): indices of alcohol use, craving, etc. (patient self-report measures) | N/A | N/A | +1 Paper Submitted  *1 Paper Submitted | N/A |
| **The Efficacy of a Smartphone-based Support System to Reinforce Alcohol Abstinence in Treatment-seeking Patients**  ^80^  Ming-Chyi Huang  Unknown  Taiwan  Ming-Chyi Huang, MD, PhD  [NCT02385643](https://clinicaltrials.gov/show/NCT02385643) | March 11, 2015  N/A  March 11, 2015 | N/A  Interventional (Clinical Trial)  Randomized  None (Open Label)  100 Participants  N/A | Experimental: Intervention Group- This group receives mobile support system (BAC test using phone application, Bluetooth sensor unit, feedback, self-management strategies, and mutual support system) and TAU.  No Intervention: Control Group- TAU | 20-50 y.o, uses Android primarily, DSM-IV diagnosis of alcohol dependence, complete abstinence for 10 days w/o withdrawal symptoms, drug screening negative  DSM-IV diagnosis of other dependence, current mental or psychiatric impairment, history of opioid abuse, history of psychosis, current use of antipsychotics, homelessness, pregnancy, cognitive deficit | (12 weeks): time to first lapse, time to first relapse, cumulative abstinence days, number of drinks per drinking days, VAS craving measurement, complete abstinence rate, drop-out rate | N/A | N/A | N/A | N/A |
| **Skills-Training for Reducing Risky Alcohol Use in App Form**  ^122^  Karolinska Institutet  Not yet recruiting  Sweden  Anne H Berman  NCT03696888 | October 5, 2018  N/A  October 24, 2018 | N/A  Interventional (Clinical Trial)  Randomized  Single (Participant)  1000 Participants  N/A | Experimental: Telecoach- Participants use a skills-training web application that teaches skills to reduce problematic alcohol use.  Active Comparator: TeleCoach Control- Participants use a web-app that provides information on consequences related to health of alcohol consumption. | 18 + y/o, excessive alcohol consumption  Severe depression, suicide risk, drug abuse, 6 or more criteria fulfilled for alcohol use disorder (DSM-5) | (26 week follow-up): Δ in alcohol consumption (7 day timeline follow back)  (baseline, 6-, 12-, 26- weeks): daily drinking questionnaire (DDQ), alcohol use disorders identification test (AUDIT), Δ in alcohol consumption (7day timeline follow back), readiness ruler, DSM-5 alcohol use disorder criteria, Penn Alcohol Craving Scale (PACS), Alcohol Abstinence Self Efficacy scale (AASE), Drug Use Disorders Identification Test (DUDIT), Montgomery Asberg Depression Rating Scale, Generalized Anxiety Disorder-7, treatment questions, usability questions | N/A | N/A | N/A | Updated October 23, 2018: no longer looking for reduction in alcohol consumption |
| **Usefulness of Supportive Text Messages in the Treatment of Depressed Alcoholics** ^81^  University of Dublin, Trinity College  Completed  Ireland  Conor Farren, PhD  NCT01037868 | December 23, 2009  N/A  March 28, 2013 | N/A  Interventional (Clinical Trial)  Randomized  Single (Outcomes Assessor)  N/A  56 Participants | Experimental: Supportive SMS Messages- Patients in the intervention group receive text messages twice a day for 3 months which encourage them to refrain from drinking alcohol. They also receive a fortnightly phone call from a member of research team to confirm continued use of the phone.  No Intervention: No Supportive SMS Text Message- Patients receive a call every fortnight to thank them for participating and a monthly call to ensure continued use. | DSM-5 criteria for alcohol dependence and comorbid for uni-polar depression, Mini Mental State Examination Score of at least 25, mobile phone and familiarity with SMS  Blind, suffer from dipolar affective disorder, history of psychosis or current diagnosis of psychotic disorder, poly-substances dependence, unavailable for follow-up | (3 months): cumulative abstinence duration (TLFB), Becks Depression Inventory Score  (3 months): Δ in gamma Glutamyl Transferase, global assessment of function score, obsessive compulsive drinking scale scores, alcohol abstinence self-efficacy scale score | N/A | N/A | N/A | Updated December 17, 2011: Becks Depression inventory Score was added, and global assessment of function score was added. |
| **Test Messaging to Reduce Alcohol Relapse in Liver Transplant Patients** ^82^  Yale University  Completed  Connecticut, United States  Benjamin Toll, PhD  NCT03402256 | January 18, 2018  N/A  January 18, 2018 | N/A  Interventional (Clinical Trial)  Randomized  Single (Care Provider)  N/A  15 Participants | Experimental: Test Message- Participants receive daily text messages (3x per day for the first 4 weeks and 3x a week for the last 4 weeks). Message topics are chosen based on content of relapse prevention treatment are sent via Google Voice on a research computer.  No Intervention: TAU | 18+ y.o, diagnosis of alcohol-related liver disease, in evaluation for UNOS as liver transplant candidate, last reported use of alcohol within past 1 year, willingness to receive and respond to text  Unstable psychiatric/ medical conditions | (8 weeks): Intervention Feasibility Evaluation (patterns of text message usage), alcohol abstinence rates (biologically confirmed rates)  (8 weeks): Subjective Alcohol Abstinence Rates (self-reported), Stress Ratings (perceived stress scale) | N/A | N/A | N/A | N/A |
| **Lifestyle Physical Activity Intervention for Depressed Alcohol Dependence** ^83^  Butler Hospital, NIAAA  Recruiting  Rhode Island, USA  Ana M Abrantes, Ph.D.  NCT02**7**05898 | March 11, 2016  N/A  April 25, 2018 | N/A  Interventional (Clinical Trial)  Randomized  None (Open Label)  70 Participants  N/A | Experimental: LPA+ Fitbit- For 12 weeks, women in intensive alcohol treatment will participate in a single in-person physical activity (PA) counselling, 6 brief phone-based PA counseling sessions to increase PA to cope with effect of alcohol cravings, use of Fitbit fitness tracker for physical activity goal setting, and weekly supportive messages via email.  Active Comparator: Health Education Contact Control (HEC)- This intervention includes an in person orientation session, 6 telephone delivered health education session, and weekly health related emails. | Engaged in alcohol treatment, meets criteria for DSM-5 alcohol use disorder, score of 1 on one of the first 2 items in patient health questionnaire, less than 150 minutes of moderate-intensity aerobic exercise, access to a computer connected to internet or a smartphone compatible with FitBit application  DSM-5 diagnosis of moderate/severe substance use disorder, history of psychotic disorder, current suicidality or homicidality, current mania, marked organic impairment, pregnant, physical or medical problems that prevent safe exercise | (6 months): percent days abstinent  (6 months): depressive symptoms (QIDS), steps/day (actigraphy), positive coping scores, level of motivation for abstinence (Contemplation ladder), self-efficacy, estimated VO2 on 6-min astrandn-thyming cycle test | N/A | N/A | N/A | N/A |

| **OPIOID USE DISORDERS** | | | | | | | | | |
| --- | --- | --- | --- | --- | --- | --- | --- | --- | --- |
| **Title, Sponsors and Collaborators, Recruitment Status, Location, Investigator, ClinicalTrials.gov Identifier** | **First Posted, Results First Posted, Last Update Posted** | **Phase, Study Type, Allocation, Masking, Estimated Enrollment, Actual Enrollment** | **Intervention** | **Eligibility Criteria: Inclusion/Exclusion** | **Primary and Secondary Outcome Measures** | **Reporting Groups: Description** | **Measures of Adherence: Planned & Posted** | **Reporting of Clinical Trials Outcome** | **Altered Outcomes** |
| **Reducing Non-Medical Opioid Use: An Automatically Adaptive mHealth Intervention** ^84^    University of Michigan, National Institute on Drug Abuse    not yet recruiting    Michigan, USA    Amy S.B Bohnert (UM)    NCT02990377 | December 13, 2016  N/A    June 11, 2018 | N/A  Interventional (Clinical Trial)  Randomized    None (Open Label)  600 participants  N/A | The study uses an interactive voice response (IVR) to ask patients about their health and medications followed by reinforcement learning (RL) through motivational intervention post emergency department visit.  Experimental: RL-Supported IVR intervention- Participants will receive brief information related to decreasing opioid analgesic risk via pamphlets given at the ED and interactive voice response and reinforcement learning supported motivational intervention for 20 minutes.  No Intervention: Enhanced Usual Care- Participants receive brief information and risk pamphlets related to opioid analgesics in the ED. | at study site ED for pain related complaint, past 3-month non-medical opioid analgesic use, receiving an OA in the ED or prescription for OA  unable to perform informed consent, pain related to acute cancer therapy, DSM-V moderate or severe opiate use disorders, unable to read/understand English, lives 50+ miles from site, acute risk for self-harm, pregnant | (1-,3-, and 6-months post-ED visit): Level of Non-Medical Opioid Use- Current Opioid Misuse Measure (COMM)  (1-,3-, and 6-months post-ED visit): ED Utilization, driving after consuming opioids, Overdose Risk Behaviors | N/A | N/A | N/A | N/A |
| **Impact on Opioid Use of Bundling Medication-assisted Treatment with mHealth (Bundling)** ^123^    University of Wisconsin, Madison, Stanley Street Treatment and Resources (SSTAR), Gosnold on Cape Cod, ARC Community Services Inc., National Institute on Drug Abuse (NIDA).  recruiting    Wisconsin, USA    David H Gustafson(UW Madison)    NCT02712034 | March 17, 2016  N/A  October 16, 2017 | N/A  Interventional (Clinical Trial)  Randomized    None (Open Label)  600 participants  N/A | In order to determine the impact on long-term opioid use medication assisted treatment (MAT) is combined with evidence based mobile health system (A-CHESS) which provide self-directed modules, information about addition, advice on how to acquire help, a channel to talk to experts, and a way to update the health care team through reports.  Experimental: MAT + A-CHESS- Patients receive MAT + A-CHESS support system. The support system provides interactive modules that teach basic recovery support, latest info about addiction, advice on how to acquire help, a way to talk to experts in the area of addiction, a way to keep healthcare team updated through A-CHESS.  Placebo: MAT + A-CHESS- Patients will receive standard medication-assisted treatment (MAT). Participants receive treatment consisting of a recovery plan, appropriate pharmacology, routine urine screens, and behavioral interventions such as monthly group counseling sessions. | at 18+ y/o, meet criteria for opioid use disorders of at least moderate severity (4 or 5 DSM-V), currently taking medication-assisted treatment (MAT) as part of their standard clinical care, no acute medical problem, no history of psychotic disorders, are willing to participate in randomized clinical trial, provide 2 contacts, able to read/write English, not pregnant, share health-related data with primary care clinicians, at intake abstinent for at least 1 week but no longer than 2 months | (24 months): Opioid Use- Detect difference in illicit opioid use between patients who have MAT + A-CHESS vs MAT alone. | N/A | N/A | N/A | N/A |
| **mHealth for Patient Self-Management of Opioid Use Disorder** ^85^  Biomedical Development Corporation  not yet recruiting  Texas, USA  Charles Bowden, MD  NCT03633929 | August 16, 2018  N/A  August 16, 2018 | N/A  Observational    N/A    N/A    20 participants  N/A | In this study, an online tool is used to better self-management of recovery. KOIS-OUD will be used, and participants will be asked to review the software.  Experimental: KIOS OUD- Participants will be enrolled in a 4-week single group pre-post evaluation. A two-hour orientation used to complete baseline assessments and then complete assessments online at least twice per week. After four-week evaluation phase, participants will return for a two-hour debriefing and completion of assessment surveys. | male or female outpatients > 18y.o, opioid use disorder assessed by MINI 6.0, currently stable in OUD outpatient treatment > 4 weeks, ability to access KIOS,OUD via comp, smartphone, or tablet  unwilling or unable to comply with study requirements, have a major psychiatric illness | (4 weeks): User Satisfaction and Usability- Systems Usability Scale-Modified (SUS), single factor with 10-item self-report scale will be used to evaluate participants’ subjective experience using the software.  (4 weeks): opioid use, depression | N/A | N/A | N/A | N/A |
| **A Mobile Application for Post-op Analgesic Consumption** ^86^  Boston University  not yet recruiting  Massachusetts, USA  Radhika Chigurupati (BU Medical)  NCT03197311 | June 23, 2017  N/A  July 27, 2018 | N/A  Interventional (Clinical Trial)  Randomized  None (Open Label)    20 participants    N/A | The purpose of the mobile application is to determine if the application can improve adherence to narcotic analgesic use and disposal. The Medable Axon Software will be used to monitor postoperative analgesic consumption, pain control, and patient satisfaction.  Experimental: Mobile App Group- In addition to standard of care, participants will be using a smartphone application to monitor postoperative analgesic consumption, disposal and pain control, and patient satisfaction for one-week post-surgery.  No Intervention: Control Group- Participants will receive standard of care which includes prescription of postoperative narcotic and NSAID analgesics and usual post-op instructions. | patients between 18-40 y.o, patients who require two mandibular third molars extracted, patients with smartphone  patients taking recreational drugs and medications for chronic pain | (7 days): assessment of quantity of post op analgesia medications used, assessment of Δ in postoperative pain control- time pills were taken and how many left at end of one week from the app  (7 days): satisfaction with the education received regarding prescribed medications, use of analgesic and post-op care education links on the mobile app- Short Form McGill Pain Questionnaire (SF-MPQ) to assess post-op pain control. | N/A | N/A | N/A | N/A |
| **Using mHealth to Aid Opioid Medication Adherence** ^87^  Care Team Solutions  Completed  Kentucky, United States  Michael Bailey, PhD  NCT02017041 | December 20, 2013  N/A  February 3, 2015 | N/A  Observational  Cohort  N/A  N/A  9 participants | The SubAID system is a smartphone application and medication monitor designed to optimize adherence behaviors for a patient responsible for administering buprenorphine/naloxone maintenance therapy medication.  Device: Medisignals- A cellular communicating medication management device designed to improve adherence and tracks when medication is removed from device at dosing times. (5-week trial)  Device: Smartphone Application- The smartphone application group is designed to support effective medication management. | 21+ y.o, physician diagnosis of Opioid dependence, prescribed buprenorphine/naloxone, able to speak + read English, willing to provide written informed consent prior to study entry, able to understand the study, iPhone or android  Concurrent medical or psychiatric condition, cognitive impairment | (5 weeks): questionnaire assessing usability of the system | N/A | N/A | N/A | N/A |
| **Using m-Health Tools to Reduce the Misuse of Opioid Pain Relievers** ^88^  Bloomberg School of Public Health, West Virginia University  Completed  Maryland, USA and West Virginia, USA  Andrea C Gielen, ScD ScM  NCT03012087 | January 6, 2017  N/A  August 22, 2017 | N/A  Interventional (Clinical Trial)  Randomized  None (Open Label)  N/A  123 Participants | Experimental: Intervention Group (MyHealthyChoices)- Participants use myhealthchoices which explains what opioid pain medications are, assess and explains the patient’s risk factors related to taking opioids, assesses patient preferences, and produces a tailored report. Patient is encouraged to use report to discuss with ED clinician.  Placebo Comparator: Control Group (Health Risk Assessment)- A WellSource health risk assessment focuses on general health promotion and the summary report is emailed to the patient’s email address. | ED visit for an injury or pain related complaint, 18+ y.o, English peaking, smartphone and email address, triage score pain between n7-10  Allergy to pain medication, use of prescription pain medication in past month, renal problems or history of dialysis | (baseline and immediate post-test): Δ self-reported preference for opioid pain reliever  (baseline and 6 weeks): Δ in knowledge about prescription pain medication side effects and safe practices for medication use, self-reported prescription drug storage and disposal behaviors | N/A | N/A | *1 Paper Submitted | N/A |
| **Mobile Intervention for Young Opioid Users** ^89^  National Development and Research Institutes, Inc  Recruiting  New York, United States  Michelle Acosta  NCT03610672 | August 1, 2018  N/A  August 2, 2018 | N/A  Interventional (Clinical Trial)  Randomized  None (Open Label)  64 Participants  N/A | Through the PI app, participants receive daily prompts and will share problems associated with opioid use with peers in social network and encourage their peers to download the PI app.  Experimental: OD training + mobile PI intervention- Participants complete a baseline assessment and overdose training (and naloxone) in addition to receiving mobile phones pre-loaded with the PI app.  Active Comparator: OD training- Participants will complete OD training and view the NYC Dept. Of Health’s 13 minute OD prevention and response video. Participants will also be given a prescription for naloxone and printed literature reviewing key training info. | Current opioid use, 18-29 y/o, speak + understand English, willing to participate in assigned intervention  Reporting of regular injection drug use | (baseline to 12 weeks): Δ in opioid use (self-reported timeline followback), Δ self-reported injection status (timeline follow back)  (baseline to 12 weeks): Δ HIV/HCV risk behavior, OD, peer IDU norms | N/A | N/A | N/A | N/A |
| **Needle-X: Usability Testing of Smartphone Application** ^124^  New York University School of Medicine  Not yet recruiting  New York, United States  Babak Tofighi  NCT03665298 | September 11, 2018  N/A  September 11, 2018 | N/A  Interventional (Clinical Trial)  Single Group Assignment  None (Open Label)  65 Participants  N/A | Experimental: Needle-X- Participants will use the smartphone application to enhance access to sterile needles through syringe exchange programs, naloxone overdose kits, and addiction treatment and free medical care programs in NYC. | Fluency in English, owns smartphone, DSM-5 criteria for current opioid use disorder  Any patient who does not meet inclusion criteria | (12 months): feasibility of needle-x application (# of patients enrolled) | N/A | N/A | N/A | N/A |
| **Using mHealth to Aid Opioid Addicts** ^125^  Care Team Solutions  Completed  Kentucky, United States  Michael Bailey, PhD  NCT01955902 | October 8, 2013  N/A  November 11, 2013 | N/A  Observational  Cohort  N/A  N/A  11 participants | This study uses focus groups to solicit opinions regarding the SubAid APP which engages and supports patients receiving treatment in order to monitor adherence. | 21+ y.o, physician diagnosis of Opioid dependence, prescribed buprenorphine/naloxone, able to speak + read English, willing to provide written informed consent prior to study entry, able to understand the study, iPhone or android  Concurrent medical or psychiatric condition, cognitive impairment | (8 weeks): attitudes, beliefs, and perceptions (recall, notes, and transcripts from session recordings) | N/A | N/A | N/A | N/A |
| **Smartphone Technology to Alleviate Malignant Pain (STAMP)** ^126^  Dana-Farber Cancer Institute, National Institute of Nursing Research (NINR)  Massachusetts, United States  Andrea Enzinger, MD  NCT03717402 | October 24, 2018  N/A  October 25, 2018 | N/A  Interventional (Clinical Trial)  Non-Randomized  None (Open Label)  70 participants  N/A | The STAMP application is a patient facing mobile phone application with a clinician web-based portal to help advanced cancer patients and providers better manage pain with opioids together.  Activity 3 (User Acceptability Testing) – Patients will use the STAMP application in an observed setting and complete a validated usability survey as well as think aloud for 30 minutes as they use STAMP.  Activity 4 (Pilot Testing)- Participants will use STAMP app for 8-weeks and will prompt patients to complete comprehensive pain assessments 3x a week and submit on-demand pain ratings. | Activity 3 Patient: Patient in the DFCI, gastrointestinal cancer center (GCC), breast oncology OR thoracic oncology programs, 21+ y.o, Diagnosed with metastatic cancer, Currently on short and long-acting opioids for cancer pain, Own a smartphone.  Activity 3 Clinician: Palliative and oncology physicians, mid-level providers, and nurses from across DFCI solid tumor programs  Activity 4 Patient: Patient in the DFCI, gastrointestinal cancer center (GCC), 21+ y.o, Diagnosed with incurable GI cancer, Chronic pain rated >4/10 within the last week, Have initiated or up-titrated a short and long-acting opioid within the last 2 weeks, Own a smartphone.  Activity 4 Clinician: Palliative and oncology physicians (and their NPs)  Activity 3 Patient Cognitive impairment, Inability to speak English, History of substance abuse  Activity 4 Patient: Cognitive impairment. Inability to speak English. History of substance abuse, Enrolled on hospice, Currently hospitalized, Use of >300mg oral morphine equivalents per day, or opioids not supported by STAMP (e.g. methadone), Diagnosed with a bowel obstruction, adults unable to consent, prisoners, and pregnant women.  Activity 3 + 4 Clinician: Unwilling to participate | Activity 3: (1 day): Patients' responses to a usability/acceptability survey as assessed by the Acceptability E-scale  Activity 4: (8 weeks): Patients' adherence to thrice weekly comprehensive symptom self-assessments (85% confidence interval), Clinicians' adherence to STAMP CDS recommendations  generalized estimating equations) | N/A | N/A | N/A | N/A |

| **EPILEPSY** | | | | | | | | | | | | | | | | | | | | | | | | |
| --- | --- | --- | --- | --- | --- | --- | --- | --- | --- | --- | --- | --- | --- | --- | --- | --- | --- | --- | --- | --- | --- | --- | --- | --- |
| **Title, Sponsors and Collaborators, Recruitment Status, Location, Investigator, ClinicalTrials.gov Identifier** | | **First Posted, Results First Posted, Last Update Posted** | | | **Phase, Study Type, Allocation, Masking, Estimated Enrollment, Actual Enrollment** | | | **Intervention** | | | **Eligibility Criteria** | | **Primary and Secondary Outcomes** | | **Reporting Groups: Description** | | | **Measures of Adherence: Planned & Posted** | | | | **Reporting of Clinical Trials Outcome** | | **Altered Outcomes** |
| **Behavioral and Educational Tools to Improve Epilepsy Care** ^20^    NYU School of Medicine, UCB Pharma  recruiting  New York, NY  Taya Spruill    NCT02646631 | | January 5, 2016    N/A    March 29, 2018 | | | N/A  Interventional (Clinical Trial)  Randomized    Single (Outcomes Assessor)    65 participants    N/A | | | MORE is a smartphone application intervention where participants can record information on a daily basis regarding seizures, medications, and stress levels. Application is equipped with a camera pill scanner, educational video clips, and self-management tips.  Experimental- MORE- Smartphone application used to record information about seizures on a daily basis (3 months).  Experimental: MORE + MI- Smartphone app and telephone based motivational interviewing (3 months).  Usual Care | | | 18+, English or Spanish speaking, partial or generalized epilepsy, poor medication adherence, ability to use smartphone.  active psychotic disorder, known or suspected psychogenic nonepileptic seizures | | (3 months): percent adherence to anti-epileptic drug schedule- pill count  (3 months): # of patients to complete study, % MI sessions completed, % diary entries completed, adherence to drug schedule, seizure frequency, quality of life | | N/A | | | N/A | | | | N/A | | N/A |
| **Stress Management Intervention for Living with Epilepsy (SMILE)** ^21^  Montefiore Medical Center, University of Cincinnati, Charles L Shor Foundation for Epilepsy Research  Completed  California, New York, Ohio- United States  Sheryl Haut, MD, Michael Privitera, MD, Susannah Cornes, MD  NCT01444183 | | September 30, 2011  N/A  April 19, 2018 | | | N/A  Interventional (Clinical Trial)  Randomized  Triple (Participant, Investigator, Outcomes Assessor)  N/A  95 participants | | | Smartphone devices will be used to identify high risk days for seizures.  Active Comparator: Progressive Muscle Relaxation (PMR)- Participants will practice PMR for 15 minutes every AM and 5 minutes every PM.  Sham Comparator: Sham Exercise- Participants practice an exercise consisting of focused attention activities. | | | 18+ y.o, English speaking, partial epilepsy, 2+ seizures/month, awareness of all seizures, able to maintain e-diary, 6^th^ grade reading level minimum, stable dose of anti-epileptic  - for clinical phase  4+ seizures in 8-week baseline, 1 seizure/ 4-week period, diaries satisfactorily completed, study compliance  Non-motor simple partial seizures, VNS use, suicide attempt, current suicidality, not competent to sign consent, behavioral techniques used for stress reduction, poor medication compliance, psychiatric illness, intermittent use of benzodiazepines | | (end of baseline, end of 12 weeks): Δ in seizure frequency | | N/A | | | N/A | | | | +1 Paper Submitted | | N/A |
| **Generalized Seizure Detection and Alerting in the EMU with The Empatica Embrace Watch and Smartphone Based Alert System** ^22^  Empatic, Inc  Recruiting  New York, USA, Italy  Rosalind W Picard, Sc. D  NCT03207685 | | July 5, 2017  N/A  February 8, 2018 | | | N/A  Interventional (Clinical Trial)  Single Group Assignment  None (Open Label)  100 participants  N/A | | | While a patient is in the epilepsy monitoring unit (EMU) they will receive an Embrace device which will identify convulsive seizures to be compares to EEG results.  Experimental: Additional Seizure Monitoring- Patients will receive EMU standard of care and Embrace. | | | Male and female patients w/ history of convulsive seizure  Patients not expected to have typical convulsive seizure during hospital administration, pregnant women, history of PNES only, known allergy to nickel or steel, infants born pre-term | | (6months): comparison of video-EEG to Embrace System- 95% confidence interval of the positive percent agreement  (6months)- alarm system comparison, false alarm rate tolerability, patient care improvement measure, embrace system usability | | N/A | | | N/A | | | | N/A | | N/A |
| **Embrace: Seizure Characterization** ^23^  Empatic, Inc  Enrolling by invitation  N/A  N/A  NCT03206502 | | July 2, 2017  N/A  June 25, 2018 | | | N/A  Interventional (Clinical Trial)  Single Group Assignment  None (Open Label)  100000 participants  N/A | | | Experimental: Embrace + Alert App- Participants will wear an Embrace smartwatch which collects data on the occurrence of seizures to send to the Alert app which issues detections or false alarms when it senses a potential convulsive seizure. Participants note all false alarms in the app. | | | 1-99 y/o, able to consent, smartphone possession, identify frequent caregiver, fluent in language of consent forms, reside in US  No broken or injured portion of wrist or leg, not homeless, no active dependence on substances, not pregnant | | (assessed continuously for 6 months): sensitivity of the GTCS detection of the Embrace + alert app  (assessed continuously for 6 months): false alarm rate of the GTCS detection of the Embrace + alert app | | N/A | | | N/A | | | | N/A | | Updated June 22, 2018: Original measures not given |
| **SCHIZOPHRENIA** | | | | | | | | | | | | | | | | | | | | | | | | |
| **Title, Sponsors and Collaborators, Recruitment Status Location, Investigator, ClinicalTrials.gov Identifier, Other Study ID Numbers** | **First Posted, Results First Posted, Last Update Posted,** | | **Phase, Study Type, Allocation, Masking, Estimated Enrollment, Actual Enrollment** | | | **Intervention** | | | | **Eligibility Criteria: Inclusion/Exclusion** | | | **Primary and Secondary Outcomes** | | | **Reporting Groups: Description** | | | **Measures of Adherence: Planned & Posted** | | **Reporting of Clinical Trials Outcome** | | | **Altered Outcomes** |
| **Study of m-RESIST, an m-Health Program for Treatment-resistant Schizophrenia** ^43^  Fundació Institut de Recerca de l’Hospital de la Santa Creu i Sant Pau, Sheba Medical Center, Semmelweis University, iMinds, AQuAS, Agència de Qualitat i Avaluació Sanitàries  Recruiting    Hungary, Israel, Spain  Corripio Illuminada, MD, PhD  NCT03064776 | February 27, 2017  N/A    March 10, 2017 | | N/A  Interventional (Clinical Trial)  Non-Randomized    None (Open Label)    45 participants    N/A | | | The study uses m-RESIST an m-health intervention program that develops, tests, and evaluates an application to allow patients to self-manage their condition.  Experimental: m-RESIST Patients- m-RESIST is a system that is designed to improve self-management of illness and facilitate recovery through capturing multidimensional behavior as it occurs in real-time and detect individual early warning signed and trigger targeted interventions using CDSS and recommender operation.  Experimental: m-RESIST Caregivers- m=RESIST aims to improve illness self-knowledge and facilitate involvement of caregivers in treating individuals with schizophrenia resistant to treatment. | | | | DSM-V diagnosis of schizophrenia meet criteria for treatment resistant schizophrenia, duration of disease < 15 years, used to information and communication technologies, presence of caregiver, both patient and caregiver sign consent  meet criteria for remission, presence of delusions, hearing, vision, or motor impairment, intellectual development disability | | | (Δ from baseline at 1 and ,3-months scores): Δ in Patient Activation Measure (PAM)  (Δ from baseline at 1 and ,3-months scores): Δ in Technology Acceptance Model (TAM), Δ in EuroQol-5D Health Questionnaire, Δin Client Satisfaction Questionnaire, Δ in Positive and Negative Syndrome Scale for Schizophrenia, Δ in Neuropsychological Assessment, Δ in Clinical Global Impression- Schizophrenia, Δ in Social Functioning Scale | | | N/A | | | N/A | | N/A | | | N/A |
| **Mobile Health Technology to Enhance Abstinence in Smokers with Schizophrenia** ^49^    Duke University  recruiting  North Carolina, USA  Jean C Beckham, Ph.D.  NCT02420015 | April 17, 2015  N/A    November 6, 2017 | | N/A    Interventional (Clinical Trial)    Randomized  None (Open Label)  36 participants    N/A | | | iCOMMIT is a smoking cessation treatment combining mobile technology, behavioral strategies, counseling, and medication.  Experimental: iCOMMIT- Patients receive behavioral therapy through mobile contingency management, pharmacotherapy for smoking cessation and nicotine replacement therapy, four sessions of guideline based cognitive behavioral smoking cessation counselling, a smart-phone based relapse prevention application, and SMS texting to increase medication adherence.  Active Comparator: Patients will receive pharmacotherapy for smoking cessation with nicotine replacement therapy and four sessions of guideline based cognitive behavioral smoking cessation counseling. | | | | At least smoke 10 cigarettes a day, have been smoking for 1+ years, meet criteria for schizophrenia, can speak/write English fluently, between 18-70 y.o, willing to make smoking cessation attempt, score > 26 on Montreal Cognitive Assessment  history of myocardial infarction in past 6 months, contraindication to NRT with no medical clearance, unwillingness to stop, pregnant, meet criteria for manic episode, enrolled in another smoking cessation trial, imprisoned | | | (6 month follow up): #of participants who self-report prolonged abstinence, # of participants whose prolonged abstinence is bio-verified  # of participants who report 7-day point prevalence abstinence (3 months post quit attempt), # of participants who report 30-day point prevalence abstinence (3 months post quit attempt), # of participants who report 7-day point prevalence abstinence (6 months post quit attempt), # of participants who report 30-day point prevalence abstinence (6 months post quit attempt) | | | N/A | | | N/A | | N/A | | | N/A |
| **Development of a Mobile System for Self-Management of Schizophrenia (SOS)** ^44^    Dartmouth-Hitchcock Medical Center    completed    New Hampshire, USA  Dror Ben-Zeev, PhD  NCT01969500 | October 25, 2013  **N/A**    February 8, 2017 | | N/A  Interventional (Clinical Trial)  Randomized    None (Open Label)    N/A    50 participants | | | The mobile Self-Management of Schizophrenia (SOS) administers interventions targeting persistent symptoms of psychosis, poor medication adherence, and social dysfunction in order to create a system that users can use in their own environments.  Experimental: Self-Management of Schizophrenia- Participants will use a mobile application system designed to improve coping with psychotic symptoms, social functioning, and medication adherence daily,  Active Comparator: TAU | | | | DSM-IV criteria for schizophrenia, 18+ y.o, prescribed oral antipsychotic medications  hearing, vision, or motor impairment, English reading level below 4th grade | | | (baseline, week 12, week 24): Δ in Severity of Psychotic Symptoms- Positive and Negative Syndrome Scale (PANSS), Δ in Social Functioning- Social Functioning Scale (SFS)  Δ in System Use (every week 1-24), Usability and Satisfaction (week 24) | | | N/A | | | N/A | | Results Submitted on August 1, 2018- pending quality control review | | | Updated June 2, 2016: No longer tracking medication adherence. |
| **A New Paradigm for Illness Monitoring and Relapse Prevention in Schizophrenia** ^48^  Dartmouth- Hitchcock Medical Center  active, not yet recruiting    New York, USA  Dror Ben-Zeev, PhD  NCT01952041 | September 27, 2013  N/A    February 8, 2017 | | N/A    Interventional (Clinical Trial)    Randomized    None (Open Label)  n/a  150 participants | | | The ecological momentary assessment (EMA) and sensor technologies are used to provide mobile monitoring of schizophrenia with the goal of detecting early signs of relapse. The system aims to capture behavior in real-time, detect warning signs, and trigger interventions that can mitigate the severity of relapse.  Experimental: Smartphone- Patients in smartphone arm will receive tau and a smartphone with the intervention application which uses sensor streams to continuously capture multidimensional behavior in real time and identify relapse signatures and prompts individual and clinical team to enhance services.  Active Comparator: TAU | | | | DSM-IV criteria for schizophrenia, 18+ y.o, inpatient psychiatric hospitalization  hearing, vision, or motor impairment, below 6th grade reading level | | | (1 year): Proportion of Relapses, psychiatric hospitalization, increase in level of psychiatric care, Time to Relapse- total of days from study commencement to first relapse  (every 3 months for a year): psychotic symptom severity, depression, social functioning | | | N/A | | | N/A | | N/A | | | Updated June 2, 2016: Now using BRPS to assess psychotic symptom severity instead of PANSS |
| **The Efficacy of Using a Smartphone App to Support Shared Decision Making in People with a Diagnosis of Schizophrenia** ^45^  Mental Health Services in the Capital Region, Denmark, TrygFonden, Denmark, Odense Patient Data Explorative Network    not yet recruiting  n/a  Lisa Kosbek, Ph.D  NCT03554655 | June 13, 2018  N/A  June 13, 2018 | | N/A  Interventional (Clinical Trial)  Randomized  Double (Investigator, Outcomes Assessor)  260 participants  n/a | | | Evaluates the effects of using a smartphone app for shared decision making (SDM). Momentum App was developed to support people with schizophrenia spectrum disorders in preparation for consultations to better involve themselves in decisions. Intended to promote collaboration with provider, awareness and eliciting of one’s needs, values, and preferences.  Experimental: Momentum App- TAU plus the Momentum system which consists of a smartphone app and web portal and aids in supporting shared decision making.  No Intervention: TAU | | | | diagnosis of schizophrenia, adults 18+. Patient received treatment for a minimum of 6 months and max of 18  do not understand or speak Danish, unable to give written informed consent, participation in other research studies involving OPUS treatment, no access to smartphone, mental retardation or dementia | | | (baseline, 3months, 6months): Δ in Patient Activation- Patient Activation Measure - Mental Health Version (PAM-MH)  (every 3 months for a year): Δ in self-efficacy, Δ in preparedness for decision making, Δ in hope, Δ in efficacy of interactions, Δ in treatment satisfaction, self-made questions, Δ in treatment alliance, clinical decision-making style, service engagement scale, Δ in positive symptoms, Δ in negative symptoms, Δ in level of functioning | | | N/A | | | N/A | | N/A | | | N/A |
| **MedActive: A Smartphone Intervention to Improve Adherence to AntiPsychotic Medications** ^46^  University of Maryland    completed    Maryland, USA  Julie A Kreyenbuhl  NCT01953237 | September 30, 2013  December 19, 2016    December 19, 2016 | | N/A    Interventional (Clinical Trial)    Randomized    None (Open Label)    N/A    31 participants | | | MEDactive app-based intervention provides personalized reminders to patients to take their antipsychotic medications in adherence with their prescription and query them about their use of medication, occurrence of side effects, and the presence of positive psychotic symptoms.  Experimental: MedActive- Patients complete a 1-hour training session on the app and their administration schedule will be understood and pre-programmed onto the app. The participants will use the app over three months.  Active Comparator : Control- Participants will receive a smartphone and will receive a phone call at the end of first week to troubleshoot problems and then no contact until the end. They do not use the application. | | | | capacity to provide informed consent, chart diagnosis of schizophrenia, prescribed and self-administers at least one oral antipsychotic medication, 18-64 y.o, speak/read English  visual, hearing, voice, or motor impairment | | | (3 months): Adherence to Antipsychotic Medications | | | N=31 (% of days adherent)  N=21,  MedActive group: 93.4 SD + 10.3 % of days  N=10  control group: 92.6 SD + 10.6 % of days  p-value=0.5580. | | | N/A | | P-value (0.5580) | | | N/A |
| **Real-Time Mobile Cognitive Behavioral Intervention for Serious Mental Illness** ^50^  University of California, San Diego    completed    California, USA  Colin Depp PhD  NCT02035202 | January 14, 2014  N/A    May 23, 2017 | | N/A    Interventional (Clinical Trial)    Randomized    Single (Outcomes Assessor)  N/A    255 participants | | | CBT2go is an intervention that integrates ecological momentary assessment with personalized interventions that are delivered when symptoms occur as it is equipped with location-based data and predictive modeling.  Experimental: CBT2go- Patients attend one session with a therapist to identify cognitive/behavioral strategies for mood symptoms, medication adherence, socialization, and relapse prevention. For 12 weeks, participants will answer questions through the app 3x a day and receive personalized strategies alone with bi-monthly telephone support. .  Active Comparator : EMA only- Participants will answer questions on the app 3x a day for 12 weeks but do not receive the strategies.  No Intervention: Standard Care- Participants will only participate in the assessments. | | | | male or female between 18-55 y.o, MINI diagnosis of either BD 1, SZ, outpatient participating in routine psychiatric care, no psychotropic medication c, capable of providing informed consent  diagnosis of dementia, seizures, mental retardation, or past head trauma, cannot complete assessment battery, visual acuity, symptoms in remission, participating in other interventions | | | (baseline, Δ at mid treatment (6 weeks), Δ at post-treatment (12 weeks)): Brief Psychiatric Rating Scale (BPRS-24)  Ecological Momentary Assessment (3x for 12 weeks), Birchwood Social Functioning Scale (baseline, 6 weeks, 12 weeks), PSR Toolkit (baseline, 6 weeks, 12 weeks) | | | N/A | | | N/A | | N/A | | | N/A |
| **Comparing Mobile Health (mHealth) and Clinic-Based Self-Management Intervention for Serious Mental Illness** ^47^  Dartmouth-Hitchcock Medical Center, Patient-Centered Outcomes Research Institute  completed  Illinois, USA  Dror Ben-Zeev PhD  NCT02421965 | April 21, 2015  N/A    August 8, 2018 | | N/A    Interventional (Clinical Trial)  Randomized  Single (Outcomes Assessor)  N/A:  174 participants | | | Experimental: FOCUS- Patients will be given a study smartphone with the FOCUS application which is designed to improve illness self-management and facilitate recovery through real-time assessment for 3 months. The application delivers patient initiated and system initiated real time assessment.  Active Comparator : WRAP (Wellness Recovery Action Planning- Participants will be provided with handout materials and will meet weekly in groups. WRAP is a clinic-based intervention to improve illness self-management and facilitate recovery through group sessions for 3 months. | | | | chart diagnosis of schizophrenia, 18+ y.o, rating of 3 or lower on ⅓ items from Domination by Symptoms factor  hearing, vision, or motor impairment, English reading level below 6th grade, received FOCUS or WRAP intervention in past three years | | | Patient Willingness to Enroll/Initiate the Intervention- proportion of individuals in each arm who commence intervention (1st day of WRAP or FOCUS session), Patient Engagement- weeks of WRAP sessions attended or weeks focus was used (3months), Patient Satisfaction- 5 item questionnaire on a 7-point scale (3 months), Δ in Severity of Symptoms- Symptom Checklist-9, Beck Depression inventory-2 (BDI-2), Psychotic Symptom Rating Scale (PSYRATS) (3 months, 6 months), Recovery- Recovery Assessment Scale (RAS) (3 months, 6 months), Δ in Quality of Life- 6-item quality of life scale (3 months, 6 months) | | | N/A | | | N/A | | N/A | | | N/A |
| **Feasibility and Outcomes of a Digital Health Support for the Schizophrenia Spectrum** ^51^  Centre for Addiction and Mental Health  Completed  Ontario, Canada  Sean Kidd, PhD  NCT03649815 | August 28, 2018  N/A  August 28, 2018 | | N/A    Interventional (Clinical Trial)  Single Group Assignment  None (Open Label)  N/A  38 participants | | | Experimental: use of mHealth technology- The participants will use App4Independence which helps prompts scheduling of activities relevant to social engagement, provides strategies to enhance wellness, encourages check-in on daily essential activities, provides basic health and tracking for level of wellness, anonymous peer-peer online network, and ambient sound detector to assist in identifying hallucinations. | | | | 18+ y/o, schizophrenia spectrum diagnosis, regular use of smartphone, read + speak English | | | (1month): Symptomatology (Brief Symptoms Inventory), Recovery Process Engagement (Personal Recovery Outcome Measure), Treatment Adherence (brief adherence rating scale) | | | N/A | | | N/A | | N/A | | | N/A |
| **The Application of Wearable Technology to Improve the Physical Activity Level of People with Chronic Mental Illness** ^127^  Kaohsuing Medical University Chung-Ho Memorial Hospital  Recruiting  Taiwan  Ming-De Chen Ph.D  NCT03408327 | January, 24, 2018  N/A  August 9, 2018 | | N/A    Interventional (Clinical Trial)  Randomized  Single (Participant)  90 participants  N/A | | | Experimental: Experimental Group- Participants will receive fitness wristband and smartphone application, will participate in group activities 4 times (2 hour each), LINE which promotes group interaction, and receive reminder and feedback from researcher.  Active Comparator: Control Group-  Participants will receive fitness wristband and smartphone application in addition to a health promotion manual containing information about physical activity. | | | | Diagnosis of serious mental illness, living in community + receiving community based mental health rehab services, stable psychiatric conditions, 20-64 y/o, no cognitive impairment, can provide consent  Clinically significant physical condition hindering walking activity, uncontrolled hypertension, cognitive impairment, unconscious, already exercising regularly, participation in other studies | | | (baseline, 12 weeks, 24 weeks): Δ daily physical activity data (fitness wristband app daily step counts)  (baseline, 12 weeks, 24 weeks): daily physical activity data (IPAQ-TW), body composition (BMI), cardiorespiratory fitness (6 minute walk test), muscular strength (hand-grip dynamometer), leg muscular strength and endurance (30-second sit to stand test), balance and mobility (timed up and go test), attention and processing speed (symbol digit modalities test), verbal working memory (digit span test), cognitive flexibility (stroop color word test), attitude to exercise (outcome expectations for exercise scale), behavior of exercise goal setting (exercise goal setting scale), situation encountered during exercise (exercise benefits/barriers scale), situation of social support (social support and exercise survey) | | | N/A | | | N/A | | N/A | | | N/A |
| **Feasibility and Acceptability of a Smartphone App to Assess Early Warning Signs of Psychosis Relapse (ExPRESS:2)** ^128^  Manchester Academic Health Science Centre  Completed  N/A  Emily Eisner, BA, MRes  NCT03558529 | June 15, 2018  N/A  June 15, 2018 | | N/A  Observational  Cohort  N/A  N/A  27 participants | | | The goal of the study is to develop a smartphone application to monitor the predictive value of basic symptoms as early signs of psychosis relapse. | | | | 18+ y/o, current contact with mental health services, primary clinical diagnosis of non-affective psychotic disorder, one episode of acute psychosis, antipsychotic medication, fluent in English, fixed abode, informed consent  Unable to complete screening, alcohol or drug dependence, organic factors implicated in aetiology | | | (6 months): % weekly app assessment completed per person (indicator of feasibility)  (6 months): % of participants completing at least 33% weekly app assessments, relapse rate ( 5 PANSS items), items from basic symptoms checklist, items from early signs scale, items from derived PANSS, qualitative data from interviews | | | N/A | | | N/A | | N/A | | | N/A |
| **Using Mobile Technology to Enhance Early Psychosis Treatment Delivery (RWJFGinger)** ^129^  University of California Davis, Ginger.io  Completed  California, United States  Tara A Niendam, PhD  NCT03303456 | October 6, 2017  N/A  October 6, 2017 | | N/A    Interventional (Clinical Trial)  Non-Randomized  None (Open Label)  N/A  79 participants | | | The Ginger.io mobile application allows for daily surveys to assess mood and social interaction and weekly surveys which assess symptoms and psychosocial functioning. The app passively collects movement and interaction data.  Experimental: First Episode Psychosis (FEP)- Participants will use the Ginger.io mobile application  Experimental: Clinical High Risk (CHR)- Participants will use the Ginger.io mobile application  Experimental: Health Controls (HC)- Participants will use the Ginger.io mobile application | | | | Ages 13-30, fluent in English  IQ below 70, history of neurological disorders, current substance abuse/dependence | | | (maximum of 18 months): feasibility of ginger.io app (# of enrolled and active patients vs population invited), acceptability of app (satisfaction survey ratings), brief psychiatric rating scale (BRPS), Global Functioning Scale: Social, critical incidents, CGI, Global Assessment of Functioning, Global Functioning Scale: Role  (maximum of 18 months): Medication adherence (MARS), therapeutic alliance (STAR), insight (IS), clinically relevant patterns, cost of care | | | N/A | | | N/A | | N/A | | | N/A |
| **Smartphone Applications Youth with Early Psychosis in Community Outpatient Settings (BHCOEMobi)** ^130^  University of California, Davis  Completed  California, United States  Tara A Niendam, PhD  NCT03286595 | September 18, 2017  N/A  September 18, 2017 | | N/A    Interventional (Clinical Trial)  Non-Randomized  None (Open Label)  N/A  60 participants | | | Experimental: Early Psychosis (EP)- Participants are either First Episode Psychosis (FEP) or Clinical High Risk (CHR) and will use the mobile application that gathers active data such as self-report surveys.  Experimental: Clinicians- Clinicians and the treatment team view app data on a dashboard and use the data to inform treatment. | | | | Ages 13-30, fluent in English, receiving care at one of three UC Davis affiliated psychosis clinics, CHR patients no history of psychosis  IQ below 70, history of neurological disorders, current substance abuse/dependence | | | (18 months): feasibility, acceptability, Brief Psychiatric rating scale (BRPS), critical incidents, clinical global impression-schizophrenia scale  (18 months): Medication adherence (MARS), therapeutic alliance (STAR), insight (IS) | | | N/A | | | N/A | | N/A | | | N/A |
| **Motivation and Skills Support (MASS)** ^52^  Boston University Charles River Campus, San Francisco State University  Not yet recruiting  California, USA and Massachusetts, USA  Daniel Fulford, PhD  NCT03404219 | January 19, 2018  N/A  September 6, 2018 | | N/A    Interventional (Clinical Trial)  Single Group Assignment  None (Open Label)  40 participants  N/A | | | Behavioral Intervention using a mobile phone-based application to support social skills and social motivation. The intervention integrates social skills training. | | | | Schizophrenia or schizoaffective disorder, between 18-50 y.o, fluent in English  Current (past 6 months) substance use disorder, self-reported current suicidal ideation, diagnosis of neurological disorder | | | (baseline and 3months follow up): Δ in social functioning (SFS), Δ social engagement (QLS)  (4x a day for 2 months): Δ experience-sampling-based self-reports of social motivation | | | N/A | | | N/A | | N/A | | | N/A |
| **Mobile Enhancement of Motivation in Schizophrenia** ^131^  Indiana University  Completed  Indiana, USA  Michelle Salyers  NCT03059771 | February 23, 2017  N/A  July 30, 2018 | | N/A    Interventional (Clinical Trial)  Randomized  Single (Outcomes Assessor)  N/A  59 participants | | | Experimental: Mobile Enhancement of Motivation (MEMS)- Participants receive 3 sets of interactive text messages each weekday for 8 weeks to reinforce and cue goal completion.  Active Comparator: Control- Participants still engage in goal-setting but do not receive reminders. | | | | Schizophrenia-spectrum diagnosis, text-message enabled cellphone, currently client at community mental health center, non-acute phase of illness, minimum moderate motivation, English reading level at or above 4^th^ grade | | | (8 weeks): goal completion  (baseline and 8 weeks): motivation and pleasure scale self-report, intrinsic motivation index | | | N/A | | | N/A | | N/A | | | N/A |
| **OTHER MENTAL AND SUBSTANCE USE DISORDERS** | | | | | | | | | | | | | | | | | | | | | | | | |
| **Title, Sponsors and Collaborators, Recruitment Status Location, Investigator, ClinicalTrials.gov Identifier, Other Study ID Numbers** | | **First Posted, Results First Posted, Last Update Posted,** | | **Phase, Study Type, Allocation, Masking, Estimated Enrollment, Actual Enrollment** | | | **Intervention** | | **Eligibility Criteria: Inclusion/Exclusion** | | | **Primary and Secondary Outcomes** | | **Reporting Groups: Description** | | | **Measures of Adherence: Planned & Posted** | | | **Reporting of Clinical Trials Outcome** | | | **Altered Outcomes** | |
| **Mobile Health Cognitive Stimulation in Heroin Users (Re@dict)** ^57^    Pedro Gamito, Universidade Lusófona de Humanidades e Tecnologias, Ares Do Pinhal-Associação De Recuperação De Toxicodependentes, Cáritas diocesana de Beja - Cáritas Portuguesa  Unknown  Pedro Gamito PhD  NCT02308878 | | December 4, 2014    N/A    December 4, 2014 | | N/A    Interventional (Clinical Trial)    Randomized    Single (Outcomes Assessor)  65 participants    N/A | | | Experiment: Mobile Health Cognitive Stimulation- Participants will receive cognitive stimulation using mobile technology with m-Health applications for 4 weeks 3 days/week.  No Intervention: Treatment-as-Usual- Participants in this group will receive treatment as usual for opioid dependence syndrome. | | patients who score higher than the cutoff values for their age on Mini Examination Test with no clinical scores on Symptoms Checklist Revised  patients with alcohol dependence or with history of previous neurological disorders | | | (1 months): Δ in the Results of Frontal Assessment Battery (FAB)- participants were assessed in Frontal Lobe Cognitive Function | | N/A | | | N/A | | | N/A | | | N/A | |
| **Mobile Application to Improve Care Coordination Among HIV Clinic and Substance Use Providers** ^55^  University of Texas at Austin  Enrolling by invitation    Texas, USA  Kasey Claborn PhD  NCT02906215 | | September 20, 2016  N/A    July 30, 2018 | | N/A    Interventional (Clinical Trial)    Single Group Assignment    None (Open Label  78 participants    N/A | | | The study aims to develop a secure mobile technology platform that will enable rapid communication among providers, improve dual care and coordination of patient services.  Experimental: Care Coordination Intervention- The care coordination intervention will include of a mobile application designed for treatment providers and a series of cross-trainings in HIV and addiction along with a digital health tool to improve screening, intervention, referral, and management. | | 18+, provide either HIV care of substance abuse treatment, employed at participating recruitment site  physical impairments that prevent completion of intervention, not fluent in English | | | (1-,3-, and 6-months)): Δ in interagency collaboration- measured by Levels of Collaboration scale  acceptability of the care coordination intervention, usability of the mobile application, Δ in frequency and quantity of communication, provider perception inventory, implementation climate scale, Δ in patient treatment retention in both HIV and Substance Use Services | | N/A | | | N/A | | | N/A | | | N/A | |
| **The Cedar Project: Impact of mHealth for HIV Prevention Among Young Indigenous People Who Use Illicit Drugs** ^54^    University of British Columbia, Canadian Institutes of Health Research (CIHR)  completed  British Columbia, Canada  n/a  NCT02437123 | | May 7, 2015  N/A  July 15, 2016 | | N/A    Interventional (Clinical Trial)    Randomized    None (Open Label    N/A  180 participants | | | Experimental: The Cedar Project mHealth- The Cedar Project intervention includes a package of culturally-safe supports including a mobile phone and long-distance cellular plan, weekly two-way texting, and support from community-based advocates.  No Intervention: Comparison Group- The comparison group will be sampled from The Cedar Project. | | currently enrolled in the Cedar Project, completed main Cedar Project Baseline questionnaire + 1 follow up, had not tested positive for HIV, joined study in Vancouver, alive an initiation of mHealth study. | | | (0,6, and 12 months): HIV propensity score- Factors associated with HIV will be used to build a propensity score. Δ in score will be used to determine the impact of the intervention on HIV vulnerability  (0,6, and 12 months): HIV risk, resilience, access to drug-related services, connection to culture, psychological distress | | N/A | | | N/A | | | N/A | | | N/A | |
| **Mental Health Engagement Network (MHEN)** ^56^    Lawson Health Research Institute, London Health Sciences Centre, Canadian Mental Health Institution, St. Joseph’s Health Care London  completed  Ontario, Canada  Cheryl Forchuk, PhD  NCT01473550 | | November 17, 2011  N/A    March 7, 2016 | | N/A    Interventional (Clinical Trial)    Randomized    None (Open Label)  N/A    400 participants | | | TELUS health space is a model for patients to be given handheld personal devices with personal health records in order to deliver healthcare catered to patient’s personal needs.  Experimental: Early Intervention- At phase 1 ( 3months) the 200 participants in Group 1 will be provided with a Personal Health Record through TELUS health space where they can access their individual care plan, prompts, and assessments.  Experimental: Later Intervention- Delayed implementation will be used to remaining 200 participants in group 2 are originally a control group but as phase 2 (6 months) group 2 is also added to TELUS. | | diagnosed with mood disorder or psychotic disorder, able to make informed consent, understand and speak English  Younger than 18 or older than 80 | | | (every 6 months up tp18 months): Lehman Quality of Life- Brief Version | | N/A | | | N/A | | | +1 Paper Submitted | | | N/A | |
| **Augmenting Specialty Eating Disorder Clinical Treatment with a Smartphone Application** ^60^    Nova Scotia Health Authority    recruiting  Nova Scotia, Canada  Aaron Keshen, MD, FRCPC    NCT02484794 | | June 30, 2015  N/A  January 26, 2018 | | N/A    Interventional (Clinical Trial)    Randomized    None (Open Label)  100 participants    N/A | | | The study aims to evaluate the usefulness of a smartphone application in replacing paper food journals as a self-monitoring tool.  Experimental: Treatment with Smartphone App- Patients will receive standard outpatient treatment but will use a smartphone application to replace the paper food record. They will also receive daily feedback through the app and will attend weekly nutritional counselling.  Active Comparator: Treatment as Usual- Patients will receive standard outpatient treatment-- group psychotherapy, skills training, self-monitoring, nutritional counselling, and meal support. | | eating disorder, apple or android smartphone, 17 y.o +, signed consent form  Patient has insufficient knowledge of English | | | (pre-treatment, 2 months post-treatment, 3-month follow-up): Δ in Eating Disorder Severity- Eating Disorder Examination Questionnaire (EDE-Q)  (pre-treatment, 2 months post-treatment, 3-month follow-up): Δ in coping skill use, Δ in coping self-efficacy, treatment acceptability, dropout rate | | N/A | | | N/A | | | N/A | | | N/A | |
| **Waitlist-Control Trial of Smartphone CBT for Body Dysmorphic Disorder (BDD)** ^132^  Massachusetts General Hospital, Telefonica  not yet recruiting  Massachusetts, United States  Sabine Wilhelm PhD    NCT03673046 | | September 17, 2018  N/A    September 17, 2018 | | N/A    Interventional (Clinical Trial)    Randomized    None (Open Label)  50 participants    N/A | | | The goal is to determine how effective a smartphone is in delivering cognitive behavioral therapy for patients with BDD.    Experimental: Smartphone Delivered CBT for BDD- Participants will receive smartphone delivered CBT for BDD for 12 weeks.  Experiment: 12 Week Waitlist Control- Participants will be crossed over to 12-week smartphone delivered CBT after 12 weeks of waitlist. | | 18+ y.o, appropriate for outpatient, diagnosis of primary DSM-5 BDD, score on BDD-YBOCS > equal to 20, live in USA  Psychotropic medication Δ within 2 months prior to enrollment, past participation in 4+ sessions of CBT for BDD, current substance dependence, lifetime bipolar disorder, acute- active suicidal ideation, severe comorbid major depression, personality disorder, concurring psychological treatment, no data plan on smartphone, lack of technology literacy | | | (week 0 and week 12): Δ in Scores on Yale-Brown Obsessive-Compulsive Scale Modified for BDD (BDD-YBOCS), Client Satisfaction Questionnaire (CSQ)  (week 12): treatment completion rates | | N/A | | | N/A | | | N/A | | | N/A | |
| **Smartphone Addiction Recovery Coach for Adolescents (SARC-A) Experiment** ^53^  Chestnut Health Systems    not yet recruiting  Illinois, United States  Michael L Dennis PhD  NCT03301012 | | October 4, 2017  N/A    July 17, 2018 | | N/A    Interventional (Clinical Trial)    Randomized    None (Open Label)  300 participants  N/A | | | Smartphone Addiction Recovery Coach for adolescents (SARC-A) is a smartphone w calling/texting/data plan with 2-3-minute recovery focused ecological momentary assessment 5x a day + feedback and continuous access to self-initiated momentary interventions to support recovery.  Experimental: Smartphone Assisted Relapse Prevention-  Other: Recovery Support as Usual | | discharge from an adolescent outpatient SUD treatment program, substance use during the 90 days prior to treatment, 15-18 at time of discharge, inability to read/communicate in English, does not reside or plan to stay in Chicago, unable to provide parental/guardian consent if under 18 | | | (6-month SFS minus baseline SFS): Δ in GAIN Substance Frequency Scale from Baseline to 6 months | | N/A | | | N/A | | | N/A | | | N/A | |
| **Automated Recovery Line for Medication Assisted Treatment** ^58^  Yale University, National Institute on Drug Abuse (NIDA), APT Foundation, Inc.  Completed  Connecticut, United States  Brent Moore, PhD  NCT02124980 | | April 28, 2014  N/A  July 2, 2017 | | N/A    Interventional (Clinical Trial)    Randomized    None (Open Label)  60 participants  N/A | | | Experimental: Recovery Line plus Treatment-as-usual (RL+TAU)- Participants use recovery line which is an automated computer based interactive voice response that provides CBT based modules for 12 weeks.  No Intervention: TAU | | 18+ y/o, currently receiving methadone maintenance treatment, illicit drug use in past 14 days  Suicide or homicide risk, criteria met for DSM-5 diagnosis of current psychotic or bipolar disorder, unable to read or understand English, anticipated incarceration or move, life threatening medical problems | | | (6 months): urine screens, self-reported drug use  (6 months): treatment retention, coping behaviors | | N/A | | | N/A | | | +1 Paper Submitted | | | N/A | |
| **Preventing HIV/STI in Urban Adolescents via an mHealth Primary Care Intervention** ^133^  University of Michigan  Recruiting  Michigan, United States  David Cordova, PhD  NCT03368456 | | December 11, 2017  N/A  August 20, 2018 | | N/A    Interventional (Clinical Trial)    Randomized    None (Open Label)  100 participants  N/A | | | The S4E application is loaded with modules focused on youth alcohol & drug use, HIV & STIs, and tobacco prevention and cessation. The content is based on scientific prevention principles and input from youth in order to reduce HIV./STI risk behaviors and increase testing.  Experimental: S4E App intervention  Placebo Comparator: TAU | | 14-21 y/o, sexually active, live in southeast Michigan, access to smartphone or tablet, must see enrolled clinician  Report of prior psychiatric hospitalization, visible cognitive impairment due to drug use | | | (baseline, 3 and 6 months post baseline): Δ in adolescent HIV testing  (baseline, 3 and 6 months post baseline): Δ condom less sex behaviors, Δ in drug use behaviors | | N/A | | | N/A | | | +1 Paper Submitted | | | Updated February 12, 2018: No longer tracking Δ in adolescent STI testing. | |
| **CopeSmart: Using Mobile Technology to Promote Positive Mental Health In Young People** ^134^  University College Dublin, Irish Research Council  Completed  Ireland  Rachel E Kenny, M.Psych.Sc  NCT02265978 | | October 16, 2014  N/A  July 10, 2015 | | N/A    Interventional (Clinical Trial)    Randomized    None (Open Label)  N/A  387 participants | | | CopeSmart is a mental health mobile app aimed to promote positive metal health through emotional self-awareness in adolescents.  Experimental: CopeSmart- Participants will use the copesmart app at least once a day for four weeks.  No Intervention: Control | | 14-19 y.o, enrolled in second-level education, access to an iphone, itouch, or android, parental consent | | | (baseline, 4 weeks, and 8-10 weeks): Δ in levels of emotional self-awareness (Emotional Self-Awareness Scale)  (baseline, 4 weeks, and 8-10 weeks): Δ in frequency of use of positive coping strategies, Δ in levels of well-being (WH0-5 well-being index), Δ help-seeking behavior, Δ levels of emotional distress | | N/A | | | N/A | | | +1 Paper Submitted | | | Updated October 10, 2014: Changed to use Emotional Self-Awareness Scale. | |
| **Homeless Care Management App (Link2Care)** ^59^  University of Oklahoma  Recruiting  Texas, USA and Oklahoma, USA  Michael S Businelle, Ph.D and Jennifer M Reingle, Ph.D  NCT03399500 | | January 16, 2018  N/A  May 1, 2018 | | N/A    Interventional (Clinical Trial)    Randomized    None (Open Label)  432 participants  N/A | | | Experimental: UCM + Smartphone- Participants receive standard case management and an unlimited smartphone.  Experimental: Smartphone Based Case Management (SPCM)- Group receives standard case management and an unlimited smartphone loaded with the SPCM app.  Active Comparator: Usual Case Management | | Released from Dallas County Jail in the past month, plan to reside in Dallas for the next year, enrolled in The Bridge Homeless Recovery Program, willing and able to attend baseline, randomization, and 1,3,6 month follow ups, score ≥ 4 on the REALM-SF, score >24 on the Mini-Mental State Exam.  Cannot read English, under the age of 18 | | | (6 months): number of case management sessions completed  (6 months): number of homeless nights (self-reported), number of arrests, alcohol use (TLFB), drug use (TLFB) | | N/A | | | N/A | | | *1 Paper Submitted | | | N/A | |

*Publication automatically indexed to the study by ClinicalTrials.gov identifier (NCT Number) containing information pertaining to study without results.

**ABBREVIATIONS:**

TAU- Treatment as Usual

HTAS -health technology assessment

ADL- activities of daily living

MPA- mHealth enhanced physical activity

SB sedentary behavior

mRS- modified rankin scale

IRL- in real life

y/o – Years Old

CBT- cognitive behavioral therapy

References

1. Impact of M-health based intervention on adherence to healthy physical activity after stroke. ClinicalTrials.gov Web site. https://ClinicalTrials.gov/ct2/show/NCT03507894?cond=Impact+of+M-health+based+Intervention+on+Adherence+to+Healthy+Physical+Activity+After+Stroke&rank=1. Published April 25, 2018. Updated 2018. Accessed November 27, 2018.

2. Increasing physical activity in stroke survivors using STARFISH, an interactive mobile phone app. ClinicalTrials.gov Web site. https://ClinicalTrials.gov/ct2/show/NCT02494245?cond=Increasing+Physical+Activity+in+Stroke+Survivors+Using+STARFISH&rank=2. Published July 10, 2015. Updated 2017. Accessed November 27, 2018.

3. The stroke and exercise program. ClinicalTrials.gov Web site. https://ClinicalTrials.gov/ct2/show/NCT02701998?cond=The+Stroke+and+Exercise+Program&rank=1. Published March 8, 2016. Updated 2017. Accessed November 27, 2018.

4. Stroke inpatient rehabilitation reinforcement of ACTivity (2010). ClinicalTrials.gov Web site. https://ClinicalTrials.gov/ct2/show/NCT01246882?cond=Stroke+Inpatient+Rehabilitation+Reinforcement+of+ACTivity&rank=1. Published November 23, 2010. Updated 2017. Accessed November 27, 2018.

5. Focus group study of lifelong food and nutrition assistance (LIFANA) in stroke patients and caregivers (2018). ClinicalTrials.gov Web site. https://ClinicalTrials.gov/ct2/show/NCT03635476?cond=LIFANA&rank=1. Published August 17, 2018. Updated 2018. Accessed November 27, 2018.

6. mHealth screening to prevent strokes (mSToPS) (2015). ClinicalTrials.gov Web site. https://ClinicalTrials.gov/ct2/show/NCT02506244?cond=mstops&rank=1. Published July 23, 2015. Updated 2018. Accessed November 27, 2018.

7. VR-3D movie-based education. ClinicalTrials.gov Web site. https://ClinicalTrials.gov/ct2/show/NCT03104231?cond=vr+3d&rank=1. Published April 7, 2017. Updated 2017. Accessed November 27, 2018.

8. Empowerment and mobile technology in the control of cardiovascular risk factors in patients with ischemic stroke (CARDIOSTROKE). ClinicalTrials.gov Web site. https://ClinicalTrials.gov/ct2/show/NCT03710902?term=NCT03710902&rank=1. Published October 18, 2018. Updated 2018. Accessed November 27, 2018.

9. Hispanic secondary stroke prevention initiative (HISSPI). ClinicalTrials.gov Web site. https://ClinicalTrials.gov/ct2/show/NCT02251834?cond=hispanic+stroke&rank=1. Published September 29, 2014. Updated 2018. Accessed November 28, 2018.

10. Phone-based intervention under nurse guidance after stroke. ClinicalTrials.gov Web site. https://ClinicalTrials.gov/ct2/show/NCT02568137?cond=Phone-based+Intervention+Under+Nurse+Guidance+After+Stroke&rank=1. Published October 5, 2015. Updated 2017. Accessed November 27, 2018.

11. iCare-AD: A mobile health application for caregivers of patients with dementia. ClinicalTrials.gov Web site. https://ClinicalTrials.gov/ct2/show/NCT03119259?cond=iCare-AD%3A+A+Mobile+Health+Application+for+Caregivers+of+Patients+With+Dementia&rank=1. Published April 18, 2017. Updated 2017. Accessed November 27, 2018.

12. Diabetes as an accelerator of cognitive impairment and. ClinicalTrials.gov Web site. https://ClinicalTrials.gov/ct2/show/NCT03578991?cond=Diabetes+as+an+Accelerator+of+Cognitive+Impairment+and&rank=1. Published July 6, 2018. Updated 2018. Accessed November 27, 2018.

13. Comparing smartphone technology and a memory strategy on improving prospective memory in Alzheimer’s disease. ClinicalTrials.gov Web site. https://ClinicalTrials.gov/ct2/show/NCT03384043?cond=Comparing+Smartphone+Technology+and+a+Memory+Strategy+on+Improving+Prospective+Memory+in+Alzheimer%27s+Disease&rank=1. Published December 27, 2017. Updated 2018. Accessed November 27, 2018.

14. Therapeutic efficacy of categorical language fluency smartphone game application. ClinicalTrials.gov Web site. https://ClinicalTrials.gov/ct2/show/NCT02848404?cond=Therapeutic+Efficacy+of+Categorical+Language+Fluency+Smartphone+Game+Application&rank=1. Published July 28, 2016. Updated 2018. Accessed November 27, 2018.

15. ArtontheBrain: An inclusive evidence-based cognitive health app for older adults to promote aging at home. ClinicalTrials.gov Web site. https://ClinicalTrials.gov/ct2/show/NCT02848404?cond=Therapeutic+Efficacy+of+Categorical+Language+Fluency+Smartphone+Game+Application&rank=1. Published July 28, 2016. Updated 2018. Accessed November 27, 2018.

16. Developing a mobile health pain-coping skills training program for the treatment of chronic migraine: AIM 4. ClinicalTrials.gov Web site. https://ClinicalTrials.gov/ct2/show/NCT03465826?cond=Developing+a+Mobile+Health+Pain-Coping+Skills+Training+Program+for+the+Treatment+of+Chronic+Migraine%3A+AIM+4&rank=1. Published March 14, 2018. Updated 2018. Accessed November 27, 2018.

17. RELAXaHEAD for headache patients. ClinicalTrials.gov Web site. https://ClinicalTrials.gov/ct2/show/NCT03183791?cond=RELAXaHEAD+for+Headache+Patients&rank=1. Published June 12, 2017. Updated 2018. Accessed November 27, 2018.

18. Improving health outcomes of migraine patients who present to the emergency department (2016). ClinicalTrials.gov Web site. https://ClinicalTrials.gov/ct2/show/NCT02945839?cond=Improving+Health+Outcomes+of+Migraine+Patients+Who+Present+to+the+Emergency+Department&rank=1. Published October 26, 2016. Updated 2018. Accessed November 27, 2018.

19. A study of the effect of a disease-specific migraine smart phone application (app) on participant care. ClinicalTrials.gov Web site. https://ClinicalTrials.gov/ct2/show/NCT03559088?cond=A+Study+of+the+Effect+of+a+Disease-Specific+Migraine+Smart+Phone+Application+%28App%29+on+Participant+Care&rank=1. Published June 15, 2018. Updated 2018. Accessed November 27, 2018.

20. Behavioral and educational tools to improve epilepsy care. ClinicalTrials.gov Web site. https://ClinicalTrials.gov/ct2/show/NCT02646631?cond=Behavioral+and+Educational+Tools+to+Improve+Epilepsy+Care&rank=1. Published January 5, 2016. Updated 2018. Accessed November 27, 2018.

21. Stress management intervention for living with epilepsy (SMILE). ClinicalTrials.gov Web site. https://ClinicalTrials.gov/ct2/show/NCT01444183?cond=Stress+Management+Intervention+for+Living+with+Epilepsy+%28SMILE%29&rank=1. Published September 30, 2011. Updated 2018. Accessed November 27, 2018.

22. Generalized seizure detection and alerting in the EMU with the empatica embrace watch and smartphone based alert system. ClinicalTrials.gov Web site. https://ClinicalTrials.gov/ct2/show/NCT03207685?cond=Generalized+Seizure+Detection+and+Alerting+in+the+EMU+with+The+Empatica+Embrace+Watch+and+Smartphone+Based+Alert+System&rank=1. Published July 5, 2017. Updated 2018. Accessed November 27, 2018.

23. Embrace: Seizure characterization. ClinicalTrials.gov Web site. https://ClinicalTrials.gov/ct2/show/NCT03206502?cond=.+Embrace%3A+Seizure+Characterization&rank=1. Published July 2, 2017. Updated 2018. Accessed November 27, 2018.

24. AniMovil mHealth support for depression management in a low-income country (AniMovil). ClinicalTrials.gov Web site. https://ClinicalTrials.gov/ct2/show/NCT03615118?cond=AniMovil+mHealth+Support+for+Depression+Management+in+Low-Income+Country&rank=1. Published August 3, 2018. Updated 2018. Accessed November 27, 2018.

25. Text-message-based depression for high-risk youth in the ED. ClinicalTrials.gov Web site. https://ClinicalTrials.gov/ct2/show/NCT02332239?cond=Text-Message-Based+Depression+for+High-Risk+Youth+in+the+ED&rank=1. Published January 6, 2015. Updated 2017. Accessed November 27, 2018.

26. An adaptive intervention for depression among Latinos living with HIV ClinicalTrials.gov Web site. https://ClinicalTrials.gov/ct2/show/NCT03668379?cond=An+Adaptive+Intervention+for+Depression+Among+Latinos+Living+With+HIV&rank=1. Published September 12, 2018. Updated 2018. Accessed November 27, 2018.

27. Intervention to prevent peer violence & depressive symptoms among at-risk adolescents. ClinicalTrials.gov Web site. https://ClinicalTrials.gov/ct2/show/NCT03626103?term=NCT03626103&rank=1. Published August 10, 2018. Updated 2018. Accessed November 27, 2018.

28. Cognitive behavioral therapy treatment of depression with smartphone support. ClinicalTrials.gov Web site. https://ClinicalTrials.gov/ct2/show/NCT01819025?cond=Cognitive+Behavioral+Therapy+Treatment+of+Depression+With+Smartphone+Support&rank=1. Published March 27, 2013. Updated 2014. Accessed November 27, 2018.

29. Cognitive-behavioral intervention via a smartphone app for depressive symptoms in caregivers (app depression). ClinicalTrials.gov Web site. https://ClinicalTrials.gov/ct2/show/NCT03110991?cond=Cognitive-behavioral+Intervention+via+a+Smartphone+App+for+Depressive+Symptoms+in+Caregivers&rank=1. Published April 12, 2017. Updated 2017. Accessed November 27, 2018.

30. Reducing depressive symptomatology with a smartphone app. ClinicalTrials.gov Web site. https://ClinicalTrials.gov/ct2/show/NCT03060200?term=NCT03060200&rank=1. Published February 23, 2017. Updated 2018. Accessed November 27, 2018.

31. Smartphone-enabled health coaching intervention for youth diagnosed with major depressive disorders. ClinicalTrials.gov Web site. https://ClinicalTrials.gov/ct2/show/NCT03406052?term=NCT03406052&rank=1. Published January 23, 2018. Updated 2018. Accessed November 27, 2018.

32. Technology assisted programs that promote mental health for teenagers (ProjectTECH). ClinicalTrials.gov Web site. https://ClinicalTrials.gov/ct2/show/NCT01912729?term=NCT01912729&rank=1. Published July 31, 2013. Updated 2018. Accessed November 27, 2018.

33. Study of technology-assisted treatment of adolescent depression (iTAD). ClinicalTrials.gov Web site. https://ClinicalTrials.gov/ct2/show/NCT01582581?term=NCT01582581&rank=1. Published April 20, 2012. Updated 2015. Accessed November 27, 2018.

34. Effectiveness of a technology assisted behavioral intervention in assisting people with major depressive disorder. ClinicalTrials.gov Web site. https://ClinicalTrials.gov/ct2/show/NCT00719979?term=NCT00719979&rank=1. Published July 22, 2008. Updated 2014. Accessed November 27, 2018.

35. Online peer networked collaborative learning for managing depressive symptoms (MoodTech). ClinicalTrials.gov Web site. https://ClinicalTrials.gov/ct2/show/NCT02841787?term=NCT02841787&rank=1. Published July 22, 2016. Updated 2018. Accessed November 27, 2018.

36. New technologies for cognitive behavior therapy (CBT) treatment of adolescent depression. ClinicalTrials.gov Web site. https://ClinicalTrials.gov/ct2/show/NCT01868867?cond=New+Technologies+for+Cognitive+Behavior+Therapy+%28CBT%29+Treatment+of+Adolescent+Depression&rank=1. Published June 5, 2013. Updated 2016. Accessed November 27, 2018.

37. Mental health telemetry for self-management in major depression (MHTV). ClinicalTrials.gov Web site. https://ClinicalTrials.gov/ct2/show/NCT01999010?term=NCT01999010&rank=1. Published December 3, 2013. Updated 2017. Accessed November 27, 2018.

38. Using mental health telemetry to predict relapse and re-hospitalization in mood disorders (PATH-MOD). ClinicalTrials.gov Web site. https://ClinicalTrials.gov/ct2/show/NCT01882608?term=NCT01882608&rank=1. Published June 20, 2013. Updated 2016. Accessed November 27, 2018.

39. Training and supervision program for depression management. ClinicalTrials.gov Web site. https://ClinicalTrials.gov/ct2/show/NCT02232854?cond=Training+and+Supervision+Program+for+Depression+Management&rank=1. Published September 5, 2014. Updated 2017. Accessed November 27, 2018.

40. Effectiveness of a mHealth intervention for the treatment of depression in people with diabetes or hypertension in peru (LATIN-MHPeru). ClinicalTrials.gov Web site. https://ClinicalTrials.gov/ct2/show/NCT03026426. Published January 20, 2017. Updated 2018. Accessed November 27, 2018.

41. Lifestyle intervention for young adults with serious mental illness. ClinicalTrials.gov Web site. https://ClinicalTrials.gov/ct2/show/NCT02815813?cond=Lifestyle+Intervention+for+Young+Adults+with+Serious+Mental+Illness&rank=1. Published June 28, 2016. Updated 2018. Accessed November 27, 2018.

42. Comparing mobile health (mHealth) and clinic-based self-management interventions for serious mental illness. ClinicalTrials.gov Web site. https://ClinicalTrials.gov/ct2/show/NCT02421965?term=NCT02421965&rank=1. Published April 21, 2015. Updated 2018. Accessed November 27, 2018.

43. Study of m-RESIST, an m-health program for treatment-resistant schizophrenia (m-RESIST). ClinicalTrials.gov Web site. https://ClinicalTrials.gov/ct2/show/NCT03064776?cond=Study+of+m-RESIST%2C+an+m-Health+Program+for+Treatment-resistant+Schizophrenia&rank=1. Published February 27, 2017. Updated 2018. Accessed November 27, 2018.

44. Development of a mobile system for self-management of schizophrenia (SOS). ClinicalTrials.gov Web site. https://ClinicalTrials.gov/ct2/show/NCT01969500?cond=Development+of+a+Mobile+System+for+Self-Management+of+Schizophrenia+%28SOS%29&rank=1. Published October 25, 2013. Updated 2017. Accessed November 27, 2018.

45. The efficacy of using a smartphone app to support shared decision making in people with a diagnosis of schizophrenia. ClinicalTrials.gov Web site. https://ClinicalTrials.gov/ct2/show/NCT03554655?cond=The+Efficacy+of+Using+a+Smartphone+App+to+Support+Shared+Decision+Making+in+People+with+a+Diagnosis+of+Schizophrenia&rank=1. Published June 13, 2018. Updated 2018. Accessed November 27, 2018.

46. MedActive: A smartphone intervention to improve adherence to AntiPsychotic medications ClinicalTrials.gov Web site. https://ClinicalTrials.gov/ct2/show/NCT01953237?cond=MedActive%3A+A+Smartphone+Intervention+to+Improve+Adherence+to+AntiPsychotic+Medications&rank=1. Published December 19, 2016. Updated 2016. Accessed November 27, 2018.

47. Comparing mobile health (mHealth) and clinic-based self-management interventions for serious mental illness. ClinicalTrials.gov Web site. https://ClinicalTrials.gov/ct2/show/NCT02421965?cond=Comparing+Mobile+Health+%28mHealth%29+and+Clinic-Based+Self-Management+Intervention+for+Serious+Mental+Illness&rank=1. Published April 21, 2015. Updated 2018. Accessed November 27, 2018.

48. A new paradigm for illness monitoring and relapse prevention in schizophrenia. ClinicalTrials.gov Web site. https://ClinicalTrials.gov/ct2/show/NCT01952041?cond=A+New+Paradigm+for+Illness+Monitoring+and+Relapse+Prevention+in+Schizophrenia&rank=1. Published September 27, 2013. Updated 2017. Accessed November 27, 2018.

49. Mobile health technology to enhance abstinence in smokers with schizophrenia. ClinicalTrials.gov Web site. https://ClinicalTrials.gov/ct2/show/NCT02420015?cond=Mobile+Health+Technology+to+Enhance+Abstinence+in+Smokers+with+Schizophrenia&rank=1. Published April 17, 2015. Updated 2018. Accessed November 27, 2018.

50. Real-time mobile cognitive behavioral intervention for serious mental illness. ClinicalTrials.gov Web site. https://ClinicalTrials.gov/ct2/show/NCT02035202?cond=Real-Time+Mobile+Cognitive+Behavioral+Intervention+for+Serious+Mental+Illness&rank=1. Published January 14, 2014. Updated 2017. Accessed November 27, 2018.

51. Feasibility and outcomes of a digital health support for the schizophrenia spectrum. ClinicalTrials.gov Web site. https://ClinicalTrials.gov/ct2/show/NCT03649815?cond=Feasibility+and+Outcomes+of+a+Digital+Health+Support+for+the+Schizophrenia+Spectrum&rank=1. Published August 28, 2018. Updated 2018. Accessed November 27, 2018.

52. Motivation and skills support (MASS). ClinicalTrials.gov Web site. https://ClinicalTrials.gov/ct2/show/NCT03404219?cond=Motivation+and+Skills+Support+%28MASS%29&rank=1. Published January 19, 2018. Updated 2018. Accessed November 27, 2018.

53. Smartphone addiction recovery coach for adolescents (SARC-A) experiment. ClinicalTrials.gov Web site. https://ClinicalTrials.gov/ct2/show/NCT03301012?cond=Smartphone+Addiction+Recovery+Coach+for+Adolescents+%28SARC-A%29+Experiment&rank=1. Published October 4, 2017. Updated 2018. Accessed November 27, 2018.

54. The cedar project: Impact of mHealth for HIV prevention among young indigenous people who use illicit drugs ClinicalTrials.gov Web site. https://ClinicalTrials.gov/ct2/show/NCT02437123?cond=The+Cedar+Project%3A+Impact+of+mHealth+for+HIV+Prevention+Among+Young+Indigenous+People+Who+Use+Illicit+Drugs&rank=1. Published May 7, 2015. Updated 2016. Accessed November 27, 2018.

55. Mobile application to improve care coordination among HIV clinic and substance use providers ClinicalTrials.gov Web site. https://ClinicalTrials.gov/ct2/show/NCT02906215?cond=Mobile+Application+to+Improve+Care+Coordination+Among+HIV+Clinic+and+Substance+Use+Providers&rank=1. Published September 20, 2016. Updated 2018. Accessed November 27, 2018.

56. Mental health engagement network (MHEN). ClinicalTrials.gov Web site. https://ClinicalTrials.gov/ct2/show/NCT01473550?cond=Mental+Health+Engagement+Network+%28MHEN%29&rank=1. Published November 17, 2011. Updated 2016. Accessed November 27, 2018.

57. Mobile health cognitive stimulation in heroin users (Re@dict). ClinicalTrials.gov Web site. https://ClinicalTrials.gov/ct2/show/NCT02308878?cond=Mobile+Health+Cognitive+Stimulation+in+Heroin+Users&rank=1. Published December 4, 2014. Updated 2014. Accessed November 27, 2018.

58. Automated recovery line for medication assisted treatment. ClinicalTrials.gov Web site. https://ClinicalTrials.gov/ct2/show/NCT02124980?cond=Automated+Recovery+Line+for+Medication+Assisted+Treatment&rank=1. Published April 28, 2014. Updated 2017. Accessed November 27, 2018.

59. Homeless care management app (Link2Care) ClinicalTrials.gov Web site. https://ClinicalTrials.gov/ct2/show/NCT03399500?term=NCT03399500&rank=1. Published January 16, 2018. Updated 2018. Accessed November 27, 2018.

60. Augmenting specialty eating disorder clinical treatment with a smartphone application. ClinicalTrials.gov Web site. https://ClinicalTrials.gov/ct2/show/NCT02484794?cond=Augmenting+Specialty+Eating+Disorder+Clinical+Treatment+with+a+Smartphone+Application&rank=1. Published June 30, 2015. Updated 2018. Accessed November 27, 2018.

61. Impact of preanesthetic information and behavioral intervention using smartphone on anxiety of children. ClinicalTrials.gov Web site. https://ClinicalTrials.gov/ct2/show/NCT02246062?cond=Impact+of+Preanesthetic+Information+and+Behavioral+Intervention+Using+Smartphone+on+Anxiety+of+Children&rank=1. Published September 22, 2014. Updated 2016. Accessed November 27, 2018.

62. Effect of premedication type on preoperative anxiety in children. ClinicalTrials.gov Web site. https://ClinicalTrials.gov/ct2/show/NCT03530670?cond=Effect+of+Premedication+Type+on+Preoperative+Anxiety+in+Children&rank=1. Published May 21, 2018. Updated 2018. Accessed November 27, 2018.

63. Smartphone-based exposure treatment for dental anxiety. ClinicalTrials.gov Web site. https://ClinicalTrials.gov/ct2/show/NCT03461016?cond=Smartphone-Based+Exposure+Treatment+for+Dental+Anxiety&rank=1. Published March 9, 2018. Updated 2018. Accessed November 27, 2018.

64. Effects of complementary therapies delivered via mobile technologies. ClinicalTrials.gov Web site. https://ClinicalTrials.gov/ct2/show/NCT02236455?cond=Effects+of+Complementary+Therapies+Delivered+Via+Mobile+Technologies&rank=1. Published September 10, 2014. Updated 2014. Accessed November 27, 2018.

65. Using smartphones to enhance the treatment of childhood anxiety. ClinicalTrials.gov Web site. https://ClinicalTrials.gov/ct2/show/NCT02259036?cond=Using+Smartphones+to+Enhance+the+Treatment+of+Childhood+Anxiety&rank=1. Published October 8, 2014. Updated 2018. Accessed November 27, 2018.

66. Youth mayo clinic anxiety coach pilot study. ClinicalTrials.gov Web site. https://ClinicalTrials.gov/ct2/show/NCT02205177. Published July 31, 2014. Updated 2018. Accessed November 27, 2018.

67. ACT-smart: Smartphone-supplemented iCBT for social phobia and/or panic disorder. ClinicalTrials.gov Web site. https://ClinicalTrials.gov/ct2/show/NCT01963806?cond=ACT-smart%3A+Smartphone-supplemented+iCBT+for+Social+Phobia+and%2For+Panic+Disorder&rank=1. Published October 16, 2013. Updated 2015. Accessed November 27, 2018.

68. Evaluating the psychophysiological effects of a smartphone-based mindfulness task. ClinicalTrials.gov Web site. https://ClinicalTrials.gov/ct2/show/NCT03296007?cond=Evaluating+the+Psychophysiological+Effects+of+a+Smartphone-Based+Mindfulness+Task&rank=1. Published September 28, 2017. Updated 2018. Accessed November 27, 2018.

69. The effectiveness of a smartphone application in the treatment of alcohol use disorder. ClinicalTrials.gov Web site. https://ClinicalTrials.gov/ct2/show/NCT03396887?cond=The+Effectiveness+of+a+Smartphone+Application+in+the+Treatment+of+Alcohol+Use+Disorder&rank=1. Published January 11, 2018. Updated 2018. Accessed November 27, 2018.

70. AlcoChange: An open label pilot study of smartphone monitoring for alcoholic liver disease. ClinicalTrials.gov Web site. https://ClinicalTrials.gov/ct2/show/NCT03474328?cond=AlcoChange%3A+An+Open+Label+Pilot+Study+of+Smartphone+Monitoring+for+Alcoholic+Liver+Disease&rank=1. Published March 22, 2018. Updated 2018. Accessed November 27, 2018.

71. Health mobile cognitive stimulation in alcoholics. ClinicalTrials.gov Web site. https://ClinicalTrials.gov/ct2/show/NCT01942954?cond=Health+Mobile+Cognitive+Stimulation+in+Alcoholics&rank=1. Published September 16, 2013. Updated 2013. Accessed November 27, 2018.

72. Study of mobile phone delivered intervention to reduce alcohol consumption (mROAD). ClinicalTrials.gov Web site. https://ClinicalTrials.gov/ct2/show/NCT02158949?term=NCT02158949&rank=1. Published June 9, 2014. Updated 2015. Accessed November 27, 2018.

73. Project guard: Reducing alcohol Misuse/Abuse in the national guard. ClinicalTrials.gov Web site. https://ClinicalTrials.gov/ct2/show/NCT02860442?cond=Project+Guard%3A+Reducing+Alcohol+Misuse%2FAbuse+in+the+National+Guard&rank=1. Published August 9, 2016. Updated 2017. Accessed November 27, 2018.

74. A text message behavioral intervention to reduce alcohol consumption in young adults (TRAC). ClinicalTrials.gov Web site. https://ClinicalTrials.gov/ct2/show/NCT01688245?term=NCT01688245&rank=1. Published September 19, 2012. Updated 2015. Accessed November 27, 2018.

75. A tailored physical activity smartphone app for patients with alcohol dependence. ClinicalTrials.gov Web site. https://ClinicalTrials.gov/ct2/show/NCT02958280?cond=A+Tailored+Physical+Activity+Smartphone+App+for+Patients+With+Alcohol+Dependence&rank=1. Published November 8, 2016. Updated 2018. Accessed November 27, 2018.

76. Young adult naturalistic alcohol study (YANAS) using smartphone technology in a stimulated laboratory environment. ClinicalTrials.gov Web site. https://ClinicalTrials.gov/ct2/show/NCT02841735?term=NCT02841735&rank=1. Published July 22, 2016. Updated 2018. Accessed November 27, 2018.

77. Smartphone technology: Young adult drinking (STEADY). ClinicalTrials.gov Web site. https://ClinicalTrials.gov/ct2/show/NCT02963818?cond=Smartphone+Technology%3A+Young+Adult+Drinking+%28STEADY%29&rank=1. Published November 15, 2016. Updated 2018. Accessed November 27, 2018.

78. Smartphone-paired breathalyzers and loss- and gain-framed texts for reducing drinking and driving (BESAFE). ClinicalTrials.gov Web site. https://ClinicalTrials.gov/ct2/show/NCT03335735?term=NCT03335735&rank=1. Published November 8, 2017. Updated 2018. Accessed November 27, 2018.

79. Adaptive goal-directed adherence tracking and enhancement (AGATE). ClinicalTrials.gov Web site. https://ClinicalTrials.gov/ct2/show/NCT01349985?term=NCT01349985&rank=1. Published May 9, 2011. Updated 2014. Accessed November 27, 2018.

80. The efficacy of A smartphone-based support system to reinforce alcohol abstinence in treatment-seeking patients. ClinicalTrials.gov Web site. https://ClinicalTrials.gov/ct2/show/NCT02385643?cond=The+Efficacy+of+A+Smartphone-based+Support+System+to+Reinforce+Alcohol+Abstinence+in+Treatment-seeking+Patients&rank=1. Published March 11, 2015. Updated 2015. Accessed November 27, 2018.

81. Usefulness of supportive text messages in the treatment of depressed alcoholics. ClinicalTrials.gov Web site. https://ClinicalTrials.gov/ct2/show/NCT01037868?cond=Usefulness+of+Supportive+Text+Messages+in+the+Treatment+of+Depressed+Alcoholics&rank=1. Published December 23, 2009. Updated 2013. Accessed November 27, 2018.

82. Text messaging to reduce alcohol relapse in liver transplant patients. ClinicalTrials.gov Web site. https://ClinicalTrials.gov/ct2/show/NCT03402256?term=NCT03402256&rank=1. Published January 18, 2018. Updated 2018. Accessed November 27, 2018.

83. Lifestyle physical activity intervention for depressed alcohol dependence. ClinicalTrials.gov Web site. https://ClinicalTrials.gov/ct2/show/NCT02705898?cond=Lifestyle+Physical+Activity+Intervention+for+Depressed+Alcohol+Dependence&rank=1. Published March 11, 2016. Updated 2018. Accessed November 27, 2018.

84. Reducing non-medical opioid use: An automatically adaptive mHealth intervention. ClinicalTrials.gov Web site. https://ClinicalTrials.gov/ct2/show/NCT02990377?cond=Reducing+Non-Medical+Opioid+Use%3A+An+Automatically+Adaptive+mHealth+Intervention&rank=1. Published December 13, 2016. Updated 2018. Accessed November 27, 2018.

85. mHealth for patient self-management of opioid use disorder. ClinicalTrials.gov Web site. https://ClinicalTrials.gov/ct2/show/NCT03633929?cond=mHealth+for+Patient+Self-Management+of+Opioid+Use+Disorder&rank=1. Published August 16, 2018. Updated 2018. Accessed November 27, 2018.

86. A mobile application for post-op analgesic consumption. ClinicalTrials.gov Web site. https://ClinicalTrials.gov/ct2/show/NCT03197311?cond=A+Mobile+Application+for+Post-op+Analgesic+Consumption&rank=1. Published June 23, 2017. Updated 2018. Accessed November 27, 2018.

87. Using mHealth to aid opioid medication adherence pilot study. ClinicalTrials.gov Web site. https://ClinicalTrials.gov/ct2/show/NCT02017041?cond=Using+mHealth+to+Aid+Opioid+Medication+Adherence&rank=1. Published December 20, 2013. Updated 2015. Accessed November 27, 2018.

88. Using m-health tools to reduce the misuse of opioid pain relievers. ClinicalTrials.gov Web site. https://ClinicalTrials.gov/ct2/show/NCT03012087?cond=Using+m-Health+Tools+to+Reduce+the+Misuse+of+Opioid+Pain+Relievers&rank=1. Published January 6, 2017. Updated 2017. Accessed November 27, 2018.

89. Mobile intervention for young opioid users. ClinicalTrials.gov Web site. https://ClinicalTrials.gov/ct2/show/NCT03610672?cond=Mobile+Intervention+for+Young+Opioid+Users&rank=1. Published August 1, 2018. Updated 2018. Accessed November 27, 2018.

90. Clinical effect size of an educational intervention in the home and compliance on people who suffer from stroke. ClinicalTrials.gov Web site. https://ClinicalTrials.gov/ct2/show/NCT01980641. Published November 11 2013. Updated 2017. Accessed December 31 2018, 2018.

91. The adherence and knowledge exchange heart and stroke medicines study (TAKEmeds). ClinicalTrials.gov Web site. https://ClinicalTrials.gov/ct2/show/NCT02597205?cond=TAKEmeds&rank=1. Published November 5, 2015. Updated 2018. Accessed November 27, 2018.

92. My stroke team (MYST): Stroke app pilot study. ClinicalTrials.gov Web site. https://ClinicalTrials.gov/ct2/show/NCT02230280?cond=myst+stroke&rank=1. Published September 3, 2014. Updated 2017. Accessed November 27, 2018.

93. iADAPTS to support strategy training after stroke. ClinicalTrials.gov Web site. https://ClinicalTrials.gov/ct2/show/NCT03253601?cond=iADAPTS+to+Support+Strategy+Training+After+Stroke&rank=1. Published August 18, 2017. Updated 2018. Accessed November 27, 2018.

94. Improving medication adherence through SMS (short messaging service) in adult stroke patients: A randomised controlled behaviour intervention trial. ClinicalTrials.gov Web site. https://ClinicalTrials.gov/ct2/show/NCT01986023?cond=Improving+Medication+Adherence+Through+SMS+%28Short+Messaging+Service%29+in+Adult+Stroke+Patients%3A+a+Randomised+Controlled+Behaviour+Intervention+Trial&rank=1. Published November 18, 2013. Updated 2014. Accessed November 27, 2018.

95. TeleRehab for stroke patients using mobile technology. ClinicalTrials.gov Web site. https://ClinicalTrials.gov/ct2/show/NCT02615132?cond=telerehab+stroke&rank=1. Published November 26, 2015. Updated 2017. Accessed November 27, 2018.

96. Developing accessible mHealth programs for depression management in Bolivia. ClinicalTrials.gov Web site. https://ClinicalTrials.gov/ct2/show/NCT02765542?cond=Developing+Accessible+mHealth+Programs+for+Depression+Management+in+Bolivia&rank=1. Published May 6, 2016. Updated 2016. Accessed November 27, 2018.

97. mHealth for antenatal mental health. ClinicalTrials.gov Web site. https://ClinicalTrials.gov/ct2/show/NCT02516982?cond=mHealth+for+Antenatal+Mental+Health&rank=1. Published August 6, 2015. Updated 2018. Accessed November 27, 2018.

98. Scaling up science-based mental health interventions in Latin America (DIADA). ClinicalTrials.gov Web site. https://ClinicalTrials.gov/ct2/show/NCT03392883?term=NCT03392883&rank=1. Published January 8, 2018. Updated 2018. Accessed November 27, 2018.

99. Behavioural activation-based treatment administered through smartphone. ClinicalTrials.gov Web site. https://ClinicalTrials.gov/ct2/show/NCT01463020?cond=Behavioural+Activation-Based+Treatment+Administered+Through+Smartphone&rank=1. Published November 1, 2011. Updated 2013. Accessed November 27, 2018.

100. Mobile sensing and support for depression. ClinicalTrials.gov Web site. https://ClinicalTrials.gov/ct2/show/NCT02776839?cond=Mobile+Sensing+and+Support+for+Depression&rank=1. Published May 18, 2016. Updated 2017. Accessed November 27, 2018.

101. Mobile technology to engage and link patients and providers in antidepressant treatment (MedLink). ClinicalTrials.gov Web site. https://ClinicalTrials.gov/ct2/show/NCT02583230?term=NCT02583230&rank=1. Published October 22, 2015. Updated 2018. Accessed November 27, 2018.

102. Mobile technology to engage and link patients and providers in antidepressant treatment (medlink RCT). ClinicalTrials.gov Web site. https://ClinicalTrials.gov/ct2/show/NCT01909973?cond=Mobile+Technology+to+Engage+and+Link+Patients+and+Providers+in+Antidepressant+Treatment&rank=2. Published July 29, 2013. Updated 2017. Accessed 27, November.

103. Wellness monitoring for major depressive disorder (CBN-well). ClinicalTrials.gov Web site. https://ClinicalTrials.gov/ct2/show/NCT02934334?term=NCT02934334&rank=1. Published October 14, 2016. Updated 2018. Accessed November 27, 2018.

104. Patient management of depression through technology: A study of digitally enabled engagement. ClinicalTrials.gov Web site. https://ClinicalTrials.gov/ct2/show/NCT03242213?cond=Patient+Management+of+Depression+Through+Technology%3A+A+Study+of+Digitally+Enabled+Engagement&rank=1. Published August 8, 2017. Updated 2018. Accessed November 27, 2018.

105. Effectiveness of a mobile texting intervention for people with serious mental illness. ClinicalTrials.gov Web site. https://ClinicalTrials.gov/ct2/show/NCT03062267?cond=Effectiveness+of+a+Mobile+Texting+Intervention+for+People+With+Serious+Mental+Illness&rank=1. Published February 23, 2017. Updated 2018. Accessed November 27, 2018.

106. Psychotherapeutic text messaging for depression pilot study. ClinicalTrials.gov Web site. https://ClinicalTrials.gov/ct2/show/NCT02872454?cond=Psychotherapeutic+Text+Messaging+for+Depression+Pilot+Study&rank=1. Published August 19, 2016. Updated 2016. Accessed November 27, 2018.

107. Treating depression on a day-to-day basis: Development of a tool for physicians based on a smartphone application (SMART). ClinicalTrials.gov Web site. https://ClinicalTrials.gov/ct2/show/NCT03678194?cond=Treating+Depression+on+a+Day-to-day+Basis%3A+Development+of+a+Tool+for+Physicians+Based+on+a+Smartphone+Application&rank=1. Published September 19, 2018. Updated 2018. Accessed November 27, 2018.

108. Testing the value of smartphone assessments of people with mood disorders. ClinicalTrials.gov Web site. https://ClinicalTrials.gov/ct2/show/NCT03429361?cond=Testing+the+Value+of+Smartphone+Assessments+of+People+with+Mood+Disorders&rank=1. Published February 12, 2018. Updated 2018. Accessed November 27, 2018.

109. Evolution of dark ideas when introducing or switching an antidepressant (DEPASSE). ClinicalTrials.gov Web site. https://ClinicalTrials.gov/ct2/show/NCT03327974?term=NCT03327974&rank=1. Published November 1, 2017. Updated 2017. Accessed November 27, 2018.

110. IntelliCare: Artificial intelligence in a mobile intervention for depression and anxiety (AIM). ClinicalTrials.gov Web site. https://ClinicalTrials.gov/ct2/show/NCT02176226?cond=IntelliCare%3A+Artificial+Intelligence+in+a+Mobile+Intervention+for+Depression+and+Anxiety+%28AIM%29&rank=1. Published June 27, 2014. Updated 2018. Accessed November 27, 2018.

111. Augmenting hospitalization for serious mental illness: Cognitive bias modification. ClinicalTrials.gov Web site. https://ClinicalTrials.gov/ct2/show/NCT03509181?cond=Augmenting+Hospitalization+for+Serious+Mental+Illness%3A+Cognitive+Bias+Modification&rank=1. Published April 26, 2018. Updated 2018. Accessed November 27, 2018.

112. Evaluation of text message engagement support of mindfulness smartphone applications. ClinicalTrials.gov Web site. https://ClinicalTrials.gov/ct2/show/NCT03633682?cond=Evaluation+of+Text+Message+Engagement+Support+of+Mindfulness+Smartphone+Applications&rank=1. Published August 16, 2018. Updated 2018. Accessed November 27, 2018.

113. Enhancing delivery of problem solving therapy using SmartPhone technology. ClinicalTrials.gov Web site. https://ClinicalTrials.gov/ct2/show/NCT01891734?cond=Enhancing+Delivery+of+Problem+Solving+Therapy+Using+SmartPhone+Technology&rank=1. Published July 3, 2013. Updated 2016. Accessed November 27, 2018.

114. Mobile phone sensing and outreach as adjuncts to internet-based behavioral intervention for depression. ClinicalTrials.gov Web site. https://ClinicalTrials.gov/ct2/show/NCT01107041?term=NCT01107041&rank=1. Published April 20, 2010. Updated 2017. Accessed November 27, 2018.

115. Technology enabled mental health intervention for individuals in the criminal justice system. ClinicalTrials.gov Web site. https://ClinicalTrials.gov/ct2/show/NCT03105973?cond=Technology+Enabled+Mental+Health+Intervention+for+Individuals+in+the+Criminal+Justice+System&rank=1. Published April 10, 2017. Updated 2017. Accessed November 27, 2018.

116. Connection to care: Pilot study of a mobile health tool for patients with depression and anxiety. ClinicalTrials.gov Web site. https://ClinicalTrials.gov/ct2/show/NCT02848404?cond=Therapeutic+Efficacy+of+Categorical+Language+Fluency+Smartphone+Game+Application&rank=1. Published July 28, 2016. Updated 2018. Accessed November 27, 2018.

117. Improving medical care with electronic interventions based on automated text and phone messages. ClinicalTrials.gov Web site. https://ClinicalTrials.gov/ct2/show/NCT03002311?cond=Improving+Medical+Care+With+Electronic+Interventions+Based+on+Automated+Text+and+Phone+Messages&rank=1. Published December 23, 2016. Updated 2017. Accessed November 27, 2018.

118. Youth mayo clinic anxiety coach randomized controlled trial. ClinicalTrials.gov Web site. https://ClinicalTrials.gov/ct2/show/NCT02205203?cond=Youth+Mayo+Clinic+Anxiety+Coach+Randomized+Controlled+Trial&rank=1. Published July 31, 2014. Updated 2018. Accessed November 27, 2018.

119. mWELLCARE: An integrated mHealth system for the prevention and care of chronic disease. ClinicalTrials.gov Web site. https://ClinicalTrials.gov/ct2/show/NCT02480062?cond=mWELLCARE%3A+An+Integrated+mHealth+System+for+the+Prevention+and+Care+of+Chronic+Disease+%28mWELLCARE%29&rank=1. Published June 24, 2015. Updated 2017. Accessed November 27, 2018.

120. Smartphone based continuing care for alcohol. ClinicalTrials.gov Web site. https://ClinicalTrials.gov/ct2/show/NCT02681406?cond=Smartphone+Based+Continuing+Care+for+Alcohol&rank=1. Published February 12, 2016. Updated 2017. Accessed November 27, 2018.

121. Feasibility of a smart-phone based support system for hazardous drinkers (NZStepAway). ClinicalTrials.gov Web site. https://ClinicalTrials.gov/ct2/show/NCT03553056?term=NCT03553056&rank=1. Published June 12, 2018. Updated 2018. Accessed November 27, 2018.

122. Skills-training for reducing risky alcohol use in app form. ClinicalTrials.gov Web site. https://ClinicalTrials.gov/ct2/show/NCT03696888?cond=Skills-Training+for+Reducing+Risky+Alcohol+Use+in+App+Form&rank=1. Published October 5, 2018. Updated 2019. Accessed November 27, 2018.

123. Impact on opioid use of bundling medication-assisted treatment with mHealth (bundling). ClinicalTrials.gov Web site. https://ClinicalTrials.gov/ct2/show/NCT02712034?cond=.+Impact+on+Opioid+Use+of+Bundling+Medication-assisted+Treatment+with+mHealth+%28Bundling%29&rank=1. Published March 17, 2016. Updated 2017. Accessed November 27, 2018.

124. Needle-X: Usability testing of smartphone application. ClinicalTrials.gov Web site. https://ClinicalTrials.gov/ct2/show/NCT03665298?cond=Needle-X%3A+Usability+Testing+of+Smartphone+Application&rank=1. Published September 11, 2018. Updated 2018. Accessed November 27, 2018.

125. Using mHealth to aid opioid addicts. ClinicalTrials.gov Web site. https://ClinicalTrials.gov/ct2/show/NCT01955902?cond=Using+mHealth+to+Aid+Opioid+Addicts&rank=1. Published October 8, 2013. Updated 2013. Accessed November 27, 2018.

126. Smartphone technology to alleviate malignant pain (STAMP). ClinicalTrials.gov Web site. https://ClinicalTrials.gov/ct2/show/NCT03717402?cond=Smartphone+Technology+to+Alleviate+Malignant+Pain+%28STAMP%29&rank=1. Published October 24, 2018. Updated 2018. Accessed November 27, 2018.

127. The application of wearable technology to improve the physical activity level of people with chronic mental illness. ClinicalTrials.gov Web site. https://ClinicalTrials.gov/ct2/show/NCT03408327?cond=The+Application+of+Wearable+Technology+to+Improve+the+Physical+Activity+Level+of+People+with+Chronic+Mental+Illness&rank=1. Published January 24, 2018. Updated 2018. Accessed November 27, 2018.

128. Feasibility and acceptability of a smartphone app to assess early warning signs of psychosis relapse (ExPRESS:2). ClinicalTrials.gov Web site. https://ClinicalTrials.gov/ct2/show/NCT03558529?cond=Feasibility+and+Acceptability+of+a+Smartphone+App+to+Assess+Early+Warning+Signs+of+Psychosis+Relapse+%28ExPRESS%3A2%29&rank=1. Published June 15, 2018. Updated 2018. Accessed November 27, 2018.

129. Using mobile technology to enhance early psychosis treatment delivery (RWJFGinger). ClinicalTrials.gov Web site. https://ClinicalTrials.gov/ct2/show/NCT03303456?cond=Using+Mobile+Technology+to+Enhance+Early+Psychosis+Treatment+Delivery&rank=1. Published October 6, 2017. Updated 2017. Accessed November 27, 2018.

130. Smartphone applications youth with early psychosis in community outpatient settings. ClinicalTrials.gov Web site. https://ClinicalTrials.gov/ct2/show/NCT03286595?cond=Smartphone+Applications+Youth+with+Early+Psychosis+in+Community+Outpatient+Settings&rank=1. Published September 18, 2017. Updated 2017. Accessed November 27, 2018.

131. Mobile enhancement of motivation in schizophrenia. ClinicalTrials.gov Web site. https://ClinicalTrials.gov/ct2/show/NCT03059771?cond=Mobile+Enhancement+of+Motivation+in+Schizophrenia&rank=1. Published February 23, 2017. Updated 2018. Accessed November 27, 2018.

132. Waitlist-control trial of smartphone CBT for body dysmorphic disorder (BDD). ClinicalTrials.gov Web site. https://ClinicalTrials.gov/ct2/show/NCT03673046?cond=Waitlist-Control+Trial+of+Smartphone+CBT+for+Body+Dysmorphic+Disorder+%28BDD%29&rank=1. Published September 17, 2018. Updated 2018. Accessed November 27, 2018.

133. Preventing HIV/STI in urban adolescents via an mHealth primary care intervention. ClinicalTrials.gov Web site. https://ClinicalTrials.gov/ct2/show/NCT03368456?cond=Preventing+HIV%2FSTI+in+Urban+Adolescents+via+an+mHealth+Primary+Care+Intervention&rank=1. Published December 11, 2017. Updated 2018. Accessed November 27, 2018.

134. CopeSmart: Using mobile technology to promote positive mental health in young people ClinicalTrials.gov Web site. https://ClinicalTrials.gov/ct2/show/NCT02265978?cond=CopeSmart%3A+Using+Mobile+Technology+to+Promote+Positive+Mental+Health+In+Young+People&rank=1. Published October 16, 2014. Updated 2015. Accessed November 27, 2018.
