# Supplementary material for: Characteristics of Neuropsychiatric Mobile Health Trials: Cross-Sectional Analysis of Studies Registered on ClinicalTrials.gov
Source: JMIR Mhealth Uhealth. 2020 Aug 4;8(8):e16180. doi: 10.2196/16180 (PMC7473471; doi:10.2196/16180)
Supplement: Multimedia Appendix 2 [file mhealth_v8i8e16180_app2.doc]

**Table 1: mHealth studies of the top ten neuropsychiatric conditions registered on ClinicalTrials.gov as of November 2018**

| **Condition**  **(# of trials)** | **Study Title**  **(Year of Registration)** | **# of participants and study duration** | **Description of Intervention and Outcome Measures** | **Publication or Posting of Results** |
| --- | --- | --- | --- | --- |
| **Stroke (17)** | 1. Clinical Effect Size of an Educational Intervention in the Home and Compliance on People Who Suffer From Stroke (2013) | 80 participants estimated enrollment  40 participants enrolled | Interventional (22 weeks) | *1 Abstract Submitted |
|  | 1. The Adherence and Knowledge Exchange Heart and Stroke Medicines Guide (TAKEmeds) (2015) | 190 participants enrolled | Interventional (3 months) | N/A |
|  | 1. Impact of M-health based Intervention on Adherence to Healthy Physical Activity After Stroke (2018), Grau-Pellicer M | 58 participants estimated enrollment | Interventional (8 weeks) | N/A |
|  | 1. Hispanic Secondary Stroke Prevention Initiative (HISSPI) (2014) | 300 participants estimated enrollment | Interventional (12 months) | *1 Abstract Submitted |
|  | 1. Increasing Physical Activity in Stroke Survivors Using STARFISH (2015) | 128 participants estimated enrollment | Interventional (4 months) | +1 Paper Submitted |
|  | 1. Effects of Patient-centered Stroke Educating System: A Randomized Controlled Trial (2015) | 80 participants estimated enrollment  63 participants enrolled | Interventional (4 weeks) | Posted on ClinicalTrials.gov |
|  | 1. The Stroke and Exercise Program (2016) | 70 participants estimated enrollment | Interventional (12 weeks) | N/A |
|  | 1. mHealth Screening to Prevent Strokes (mSToPS) (2015) | 2274 participants estimated enrollment | Interventional (8 months) | N/A |
|  | 9. My Stroke Team (MYST): Stroke App Pilot Study (2014) | 30 participants estimated enrollment | Interventional (6 months) | N/A |
|  | 10. Stroke Inpatient Rehabilitation Reinforcement of ACTivity (2010) | 140 participants enrolled | Interventional (15 months) | +1 Paper Submitted |
|  | 11. iADAPTS to Support Strategy Training After Stroke (2017) | 30 participants estimated enrollment | Interventional (3 months)(3 months) | N/A |
|  | 12. Focus Group Study of Lifelong Food and Nutrition Assistance (LIFANA) in Stroke Patients and Caregivers (2018) | 8 participants estimated enrollment | Interventional (1 day/1.5-2 hour sessions but duration uncertain) | N/A |
|  | 13. VR-3D Movie-based Education (2017) | 100 participants enrolled | Interventional (1 year) | +1 Paper Submitted |
|  | 14. Improving Medication Adherence Through SMS (Short Messaging Service) in Adult Stroke Patients: a Randomised Controlled Behaviour Intervention Trial (2013) | 200 participants enrolled | Interventional (2 months) | +1 Paper Submitted  *1 Paper Submitted |
|  | 15. Phone-based Intervention Under Nurse Guidance After Stroke (2015) | 60 participants enrolled | Interventional (9 months) | +2 Papers Submitted  *1 Paper Submitted |
|  | 16. TeleRehab for Stroke Patients Using Mobile Technology (2015) | 20 participants enrolled | Interventional (8 weeks) | N/A |
|  | 17. Empowerment and Mobile Technology in the Control of Cardiovascular Risk Factors in Patients with Ischemic Stroke (CARDIOSTROKE) (2018) | 405 participants estimated enrollment | Interventional (3 weeks or 1 week monthly) | N/A |
| **Migraine (4)** | 1. Developing a Mobile Health Pain-Coping Skills Training Program for the Treatment of Chronic Migraine: AIM 4 (2018) | 144 participants estimated enrollment | Interventional  (8 weeks) | N/A |
|  | 2. A Study of the Effect of a Disease-Specific Migraine Smart Phone Application (App) on Participant Care (2018) | 200 participants estimated enrollment | Interventional  (1 year) | N/A |
|  | 3. RELAXaHEAD for Headache Patients (2017) | 90 participants estimated enrollment | Interventional  (6 months) | N/A |
|  | I4. Improving Health Outcomes of Migraine Patients Who Present to the Emergency Department (2016) | 90 participants estimated enrollment | Interventional  (3 months) | N/A |
| **Major Depressive Disorder (39)** | 1. Developing Accessible mHealth Programs for Depression Management in Bolivia (2016) | 32 participants enrolled | Interventional (12 weeks) | N/A |
|  | 2. AniMovil mHealth Support for Depression Management in Low-Income Country (AniMovil) (2018) | 114 participants estimated enrollment | Interventional (12 weeks) | N/A |
|  | 3. mHealth for Antenatal Mental Health (2015) | 880 participants enrolled | Interventional (24 hours) | N/A |
|  | 4. Scaling Up Science-based Mental Health Interventions in Latin America (2018) | 2000 participants estimated enrollment | Interventional (12 months) | N/A |
|  | 5. Text-Message-Based Depression for High-Risk Youth in the ED (2015) | 116 participants enrolled | Interventional (8 weeks) | Results Posted on ClinicalTrials.gov |
|  | 6. An Adaptive Intervention for Depression Among Latinos Living With HIV (2018) | 45 participants estimated enrollment | Interventional (4 months) | N/A |
|  | 7. Intervention to Prevent Peer Violence and Depressive Symptoms Among At-Risk Adolescents (iDOVE2) (2018) | 800 participants estimated enrollment | Interventional (8 weeks) | N/A |
|  | 8. Cognitive Behavioral Therapy Treatment of Depression With Smartphone Support (2013) | 88 participants enrolled | Interventional (8 weeks) | +1 Paper Submitted |
|  | 9. Behavioural Activation-Based Treatment Administered Through Smartphone (2011) | 81 participants enrolled | Interventional (8 weeks) | +1 Paper Submitted  *1 Paper Submitted |
|  | 10. Cognitive-behavioral Intervention via a Smartphone App for Depressive Symptoms in Caregivers (App Depression) (2017) | 174 participants estimated enrollment | Interventional (6 weeks) | *1 Paper Submitted |
|  | 11. Reducing Depressive Symptomatology With a Smartphone App (2017) | 220 participants estimated enrollment | Interventional (6 weeks) | *1 Paper Submitted |
|  | 12. Mobile Sensing and Support for Depression (2016) | 126 participants enrolled | Interventional (6 months) | Results Submitted (Not Posted on ClinicalTrials.gov) |
|  | 13. Mobile Technology to Engage and Link Patients and Providers in Antidepressant Treatment (MedLink) (2015) | 11 participants enrolled | Interventional (8 weeks) | Posted on ClinicalTrials.gov |
|  | 14. Mobile Technology to Engage and Link Patients and Providers in Antidepressant Treatment (2013) | 15 participants enrolled | Interventional (12 weeks) | Results Submitted (Not Posted on ClinicalTrials.gov) |
|  | 15. Lifestyle Intervention for Young Adults with Serious Mental Illness (2016) | 144 participants estimated enrollment | Interventional (12 months) | N/A |
|  | 16. Wellness Monitoring for Major Depressive Disorder (CBN-Well) (2015) | 100 participants enrolled | Interventional (13 months) | N/A |
|  | 17. Comparing Mobile Health (mHealth) and Clinic-Based Self-Management Interventions for Serious Mental Illness (2016) | 174 participants enrolled | Interventional (12 weeks) | N/A |
|  | 18. Patient Management of Depression Through Technology: a Study of Digitally Enabled Engagement (2017) | 40 participants estimated enrollment | Interventional (18 weeks) | N/A |
|  | 19. Effectiveness of a Mobile Texting Intervention for People With Serious Mental Illness (2017) | 52 participants estimated enrollment | Interventional (3 months) | N/A |
|  | 20. Psychotherapeutic Text Messaging for Depression Pilot Study (2016) | 190 participants enrolled | Interventional (12 weeks) | N/A |
|  | 21. Treating Depression on a Day-to-day Basis: Development of a Tool for Physicians Based on a Smartphone Application (2018) | 200 participants estimated enrollment | Interventional (6 weeks) | N/A |
|  | 22. Mental Health Telemetry for Self-Management in Major Depressive Disorder (MHTV) (2018) | 27 participants enrolled | Interventional (2 months or 4 months) | N/A |
|  | 23. Using Mental Health Telemetry to Predict Relapse and Re-hospitalization in Mood Disorders (PATH-MOD) (2013) | 33 participants enrolled | Interventional (6 months) | N/A |
|  | 24. Effectiveness of a mHealth Intervention for the Treatment of Depression in People With Diabetes or Hypertension in Peru (LATIN-MHPeru) (2017) | 432 participants enrolled | Interventional (6 weeks) | N/A |
|  | 25. Smartphone-enabled Health Coaching Intervention for Youth Diagnosed with Major Depressive Disorders (2018 | 168 participants estimated enrollment | Interventional (6 months) | N/A |
|  | 26. Testing the Value of Smartphone Assessments of People with Mood Disorders (2018) | 40 participants estimated enrollment | Interventional (6 months) | N/A |
|  | 27. Evolution of Dark Ideas When Introducing or Switching an Antidepressant (DEPASSE) (2017) | 103 participants estimated enrollment | Interventional (1 month) | N/A |
|  | 28. IntelliCare: Artificial Intelligence in a Mobile Intervention for Depression and Anxiety (AIM) (2014) | 105 participants enrolled | Interventional (8 weeks) | Posted on ClinicalTrials.gov |
|  | 29. Augmenting Hospitalization for Serious Mental Illness: Cognitive Bias Modification (2018) | 16 participants estimated enrollment | Interventional (3 months) | N/A |
|  | 30. Evaluation of Text Message Engagement Support of Mindfulness Smartphone Applications (2018) | 40 participants estimated enrollment | Intervention (4 weeks) | N/A |
|  | 31. Enhancing Delivery of Problem Solving Therapy Using SmartPhone Technology (2013) | 33 participants enrolled | Intervention (12 weeks) | Posted on ClinicalTrials.gov |
|  | 32. Technology Assisted Programs that Promote Mental Health for Teenagers (ProjectTECH) (2013) | 41 participants enrolled | Interventional (8 weeks) | N/A |
|  | 33. New Technologies for Cognitive Behavior Therapy (CBT) Treatment of Adolescent Depression (2013) | 18 participants enrolled | Interventional (12 weeks) | +1 Paper Submitted |
|  | 34. Study of Technology-assisted Treatment of Adolescent Depression (iTAD) (2012) | 45 participants enrolled | Interventional (12 weeks) | N/A |
|  | 35. Mobile Phone Sensing and Outreach as Adjuncts to Internet Based Behavior Intervention for Depression (2018) | 2010 participants enrolled | Interventional (6 weeks) | N/A |
|  | 36. Effectiveness of a Technology Assisted Behavioral Intervention in Assisting People with Major Depressive Disorder (2008) | 102 participants enrolled | Interventional (12 weeks) | N/A |
|  | 37. Technology Enabled Mental Health Intervention for Individuals in the Criminal Justice System (2017) | 65 participants estimated enrollment | Interventional (4 weeks) | N/A |
|  | 38. Training and Supervision Program for Depression Management (2014) | 256 participants enrolled | Interventional (6 months) | N/A |
|  | 39. Online Peer Networked Collaborative Learning for Managing Depressive Symptoms (MoodTech) (2016) | 47 participants enrolled | Interventional (8 weeks) | Results posted on ClinicalTrials.gov |
| **Alzheimer Disease And Other Dementias (5)** | 1. iCare-AD: A Mobile Health Application for Caregivers of Patients With Dementia (2017) | 448 participants estimated enrollment | Interventional (12 months) | N/A |
|  | 2. Diabetes as an Accelerator of Cognitive Impairment and Alzheimer’s Disease (2018) | 174 participants estimated enrollment | Interventional (18 months) | N/A |
|  | 3. Comparing Smartphone Technology and a Memory Strategy on Improving Prospective Memory in Alzheimer's Disease (2017) | 52 participants estimated enrollment | Interventional (4 weeks) | N/A |
|  | 4. Therapeutic Efficacy of Categorical Language Fluency Smartphone Game Application (2016) | 20 participants actual enrollment | Interventional (4 weeks) | N/A |
|  | 5. ArtontheBrain: An Inclusive Evidence-based Cognitive Health App for Older Adults to Promote Aging at Home (2018) | 110 participants estimated enrollment | Interventional (6 weeks) | N/A |
| **Anxiety Disorders (11)** | 1. Connection to Care: Pilot Study of a Mobile Health Tool for Patients with Depression and Anxiety (2015) | 18 participants enrolled | Interventional (16 weeks) | Results posted on ClinicalTrials.gov |
|  | 2. Improving Medical Care With Electronic Interventions Based on Automated Text and Phone Messages (2016) | 5000 participants estimated enrollment | Interventional (5 years) | N/A |
|  | 3. Impact of Preanesthetic Information and Behavioral Intervention Using Smartphone on Anxiety of Children (2014) | 84 participants enrolled | Interventional (24 hours before surgery) | Results posted on ClinicalTrials.gov  +1 Paper Submitted |
|  | 4. Evaluating the Psychophysiological Effects of a Smartphone-Based Mindfulness Task (2017) | 180 participants estimated enrollment | Interventional (12 minutes) | N/A |
|  | 5. Effect of Premedication Type on Preoperative Anxiety in Children (2018) | 138 participants estimated enrollment | Interventional (Before premedication, 20 minutes after premedication, at anesthesia induction, postoperative every 10 minutes ) | N/A |
|  | 6. Using Smartphones to Enhance the Treatment of Childhood Anxiety (2014) | 40 participants enrolled | Interventional  (10 weeks) | N/A |
|  | 7. Smartphone-Based Exposure Treatment for Dental Anxiety (2018) | 40 participants estimated enrollment | Interventional (2 weeks) | N/A |
|  | 8. Youth Mayo Clinic Anxiety Coach Pilot Study (2017) | 10 participants enrolled | Interventional (least 6 weeks and up to 12 weeks) | N/A |
|  | 9. Youth Mayo Clinic Anxiety Coach Randomized Controlled Trial (2014) | 70 participants estimated enrollment | Interventional (6 to 12 50-minute face-to-face therapy sessions) | N/A |
|  | 10.ACT-smart: Smartphone-supplemented iCBT for Social Phobia and/or Panic Disorder (2014) | 150 participants estimated enrollment | Interventional (10 weeks) | N/A |
|  | 11. Effects of Complementary Therapies Delivered Via Mobile Technologies (2014) | 105 participants enrolled | Interventional (10 days) | +1 Paper Submitted |
| **Alcohol Use Disorders (19)** | 1. The Effectiveness of a Smartphone Application in the Treatment of Alcohol Use Disorder (2018) | 50 participants estimated | Interventional (3 months) | N/A |
|  | 2. AlcoChange: An Open Label Pilot Study of Smartphone Monitoring for Alcoholic Liver Disease (2018) | 60 participants estimated | Interventional (3 months) | N/A |
|  | 3. Health Mobile Cognitive Stimulation in Alcoholics (2013) | 54 participants enrolled | Interventional (4 weeks) | N/A |
|  | 4. mWELLCARE: An Integrated mHealth System for the Prevention and Care of Chronic Disease (mWELLCARE) (2014) | 3702 participants enrolled | Interventional (12 months) | N/A |
|  | 5. Study of Mobile Phone Delivered Intervention to Reduce Alcohol Consumption (mROAD) (2015) | 100 participants estimated | Interventional (1 week) | N/A |
|  | 6. Smartphone Based Continuing Care for Alcohol | 280 participants estimated | Interventional (12 months) | N/A |
|  | 7. Project Guard: Reducing Alcohol Misuse/Abuse in the National Guard (2016) | 750 participants estimated | Interventional (12 months) | N/A |
|  | 8. A Text Message Behavioral Intervention to Reduce Alcohol Consumption in Young Adults (TRAC) (2016) | 765 participants enrolled | Interventional (12 weeks) | +1 Paper Submitted  *1 Paper Submitted |
|  | 9. A Tailored Physical Activity Smartphone App for Patients With Alcohol Dependence (2012) | 30 participants estimated | Interventional (12 weeks) | N/A |
|  | 10. Young Adult Naturalistic Alcohol Study (YANAS) Using Smartphone Technology in a Stimulated Laboratory Environment (2016) | 129 participants estimated | Observational (2 months) | N/A |
|  | 11. Feasibility of a Smart-phone Based support System for Hazardous Drinkers (NZStepAway) (2016) | 200 participants estimated | Interventional (6 months) | N/A |
|  | 12. Smartphone Technology: Young Adult Drinking (STEADY) (2018) | 109 participants estimated | Interventional (2 months) | N/A |
|  | 13. Smartphone-paired Breathalyzers and Loss- and Gain-framed Texts for Reducing Drinking and Driving (BESAFE) (2016) | 58 participants enrolled | Interventional (8 weeks) | N/A |
|  | 14. Adaptive Goal-Directed Adherence Tracking and Enhancement (AGATE) (2017) | 136 participants enrolled | Interventional (8 weeks) | +1 Paper Submitted  *1 Paper Submitted |
|  | 15. The Efficacy of A Smartphone-based Support System to Reinforce Alcohol Abstinence in Treatment-seeking Patients (2015) | 100 participants estimated | Interventional (12 weeks) | N/A |
|  | 16. Skills-Training for Reducing Risky Alcohol Use in App Form (2018) | 1000 participants estimated | Interventional (26 weeks) | N/A |
|  | 17. Usefulness of Supportive Text Messages in the Treatment of Depressed Alcoholics (2009) | 56 participants enrolled | Interventional (3 months) | N/A |
|  | 18. Text Messaging to Reduce Alcohol Relapse in Liver Transplant Patients (2018) | 15 participants enrolled | Interventional (8 weeks) | N/A |
|  | 19. Lifestyle Physical Activity Intervention for Depressed Alcohol Dependence (2016) | 70 participants estimated | Interventional (12 weeks) | N/A |
| **Opioid Use Disorders (10)** | 1. Reducing Non-Medical Opioid Use: An Automatically Adaptive mHealth Intervention (2016) | 600 participants estimated | Interventional (6 months) | N/A |
|  | 2. Impact on Opioid Use of Bundling Medication-assisted Treatment with mHealth (Bundling) (2016) | 600 participants estimated | Interventional (24 months) | N/A |
|  | 3. mHealth for Patient Self-Management of Opioid Use Disorder (2018) | 20 participants estimated | Observational (4 weeks) | N/A |
|  | 4. A Mobile Application for Post-op Analgesic Consumption (2017) | 20 participants estimated | Interventional (1 week) | N/A |
|  | 5. Using mHealth to Aid Opioid Medication Adherence (2013) | 9 participants enrolled | Interventional (5 weeks) | N/A |
|  | 6. Using m-Health Tools to Reduce the Misuse of Opioid Pain Relievers (2017) | 123 participants enrolled | Interventional (6 weeks) | *1 Paper Submitted |
|  | 7. Mobile Intervention for Young Opioid Users (2018) | 64 participants estimated | Interventional (12 weeks) | N/A |
|  | 8. Needle-X: Usability Testing of Smartphone Application (2018) | 65 participants estimated | Interventional (12 months) | N/A |
|  | 9. Using mHealth to Aid Opioid Addicts (2013) | 11 participants estimated | Interventional (8 weeks) | N/A |
|  | 10. Smartphone Technology to Alleviate Malignant Pain (STAMP) (2018) | 70 participants estimated | Interventional (8 weeks) | N/A |
| **Epilepsy (4)** | 1. Behavioral and Educational Tools to Improve Epilepsy Care (2016) | 65 participants estimated | Interventional (3 months) | N/A |
|  | 2. Stress Management Intervention for Living with Epilepsy (SMILE) (2011) | 95 participants enrolled | Interventional (12 weeks) | +1 Paper Submitted |
|  | 3. Generalized Seizure Detection and Alerting in the EMU with The Empatica Embrace Watch and Smartphone Based Alert System (2017) | 100 participants estimated | Interventional (6 months) | N/A |
|  | 4. Embrace: Seizure Characterization (2017) | 1000 participants estimated | Interventional (6 months) | N/A |
| **Schizophrenia (15)** | 1. Study of m-RESIST, an m-Health Program for Treatment-resistant Schizophrenia (2017) | 45 participants estimated | Interventional (3 months) | N/A |
|  | 2. Mobile Health Technology to Enhance Abstinence in Smokers with Schizophrenia (2015) | 36 participants estimated | Interventional (6 months) | N/A |
|  | 3. Development of a Mobile System for Self-Management of Schizophrenia (SOS) (2013) | 50 participants enrolled | Interventional (24 weeks) | Results Submitted Aug 1, 2018- Pending Quality Control Review |
|  | 4. A New Paradigm for Illness Monitoring and Relapse Prevention in Schizophrenia (2013) | 150 participants enrolled | Interventional (1 year) | N/A |
|  | 5. The Efficacy of Using a Smartphone App to Support Shared Decision Making in People with a Diagnosis of Schizophrenia (2018) | 260 participants estimated | Interventional (6 months) | N/A |
|  | 6. MedActive: A Smartphone Intervention to Improve Adherence to AntiPsychotic Medications (2013) | 31 participants enrolled | Interventional (3 months) | Posted on ClinicalTrials.gov |
|  | 7. Real-Time Mobile Cognitive Behavioral Intervention for Serious Mental Illness (2014) | 255 participants enrolled | Interventional  (12 weeks) | N/A |
|  | 8. Comparing Mobile Health (mHealth) and Clinic-Based Self-Management Intervention for Serious Mental Illness (2015) | 174 participants enrolled | Interventional (3 months) | N/A |
|  | 9. Feasibility and Outcomes of a Digital Health Support for the Schizophrenia Spectrum (2018) | 38 participants enrolled | Interventional(1 month) | N/A |
|  | 10. The Application of Wearable Technology to Improve the Physical Activity Level of People with Chronic Mental Illness (2018) | 90 participants estimated | Interventional (24 weeks) | N/A |
|  | 11. Feasibility and Acceptability of a Smartphone App to Assess Early Warning Signs of Psychosis Relapse (ExPRESS:2) (2018) | 27 participants enrolled | Interventional (6 months) | N/A |
|  | 12. Using Mobile Technology to Enhance Early Psychosis Treatment Delivery (RWJFGinger) (2017) | 79 participants enrolled | Interventional (18 months) | N/A |
|  | 13. Smartphone Applications Youth with Early Psychosis in Community Outpatient Settings (BHCOEMobi) (2017) | 60 participants enrolled | Interventional (18 months) | N/A |
|  | 14. Motivation and Skills Support (MASS) (2018) | 40 participants estimated | Interventional (2 months) | N/A |
|  | 15. Mobile Enhancement of Motivation in Schizophrenia (2017) | 59 participants enrolled | Interventional (8 weeks) | N/A |
| **Other Mental and Substance Use Disorders (11)** | 1. Mobile Health Cognitive Stimulation in Heroin Users (Re@dict) (2014) | 65 participants estimated | Interventional (1 month) | N/A |
|  | 2. Mobile Application to Improve Care Coordination Among HIV Clinic and Substance Use Providers (2016) | 78 participants estimated | Interventional (6 months) | N/A |
|  | 3. The Cedar Project: Impact of mHealth for HIV Prevention Among Young Indigenous People Who Use Illicit Drugs (2015) | 180 participants enrolled | Interventional(12 months) | N/A |
|  | 4. Mental Health Engagement Network (MHEN) (2011) | 400 participants enrolled | Interventional (18 months) | +1 Paper Submitted |
|  | 5. Augmenting Specialty Eating Disorder Clinical Treatment with a Smartphone Application (2015) | 100 participants estimated | Interventional (8 months) | N/A |
|  | 6. Waitlist-Control Trial of Smartphone CBT for Body Dysmorphic Disorder (BDD) (2018) | 50 participants estimated | Interventional (12 weeks) | N/A |
|  | 7. Smartphone Addiction Recovery Coach for Adolescents (SARC-A) Experiment (2017) | 300 participants estimated | Interventional (6 months) | N/A |
|  | 8. Automated Recovery Line for Medication Assisted Treatment (2014) | 60 participants estimated | Interventional (6 months) | +1 Paper Submitted |
|  | 9. Preventing HIV/STI in Urban Adolescents via an mHealth Primary Care Intervention (2017) | 100 participants estimated | Interventional (6 months) | +1 Paper Submitted |
|  | 10. CopeSmart: Using Mobile Technology to Promote Positive Mental Health In Young People (2014) | 387 participants enrolled | Interventional (10 weeks) | +1 Paper Submitted |
|  | 11. Homeless Care Management App (Link2Care) (2018) | 432 participants estimated | Interventional (6 months) | *1 Abstract Submitted |
